# Comprehensive cross-sectional and longitudinal comparisons of plasma glial fibrillary acidic protein and neurofilament light across FTD spectrum disorders

**DOI:** 10.1186/s13024-025-00821-4

**Published:** 2025-03-12

**Authors:** Udit Sheth, Linn Öijerstedt, Michael G. Heckman, Launia J. White, Hilary W. Heuer, Argentina Lario Lago, Leah K. Forsberg, Kelley M. Faber, Tatiana M. Foroud, Rosa Rademakers, Eliana Marisa Ramos, Brian S. Appleby, Andrea C. Bozoki, R. Ryan Darby, Bradford C. Dickerson, Kimiko Domoto-Reilly, Douglas R. Galasko, Nupur Ghoshal, Neill R. Graff-Radford, Ian M. Grant, Chadwick M. Hales, Ging-Yuek Robin Hsiung, Edward D. Huey, David Irwin, Justin Y. Kwan, Irene Litvan, Ian R. Mackenzie, Joseph C. Masdeu, Mario F. Mendez, Chiadi U. Onyike, Belen Pascual, Peter S. Pressman, Erik D. Roberson, Allison Snyder, M. Carmela Tartaglia, William W. Seeley, Dennis W. Dickson, Howard J. Rosen, Bradley F. Boeve, Adam L. Boxer, Leonard Petrucelli, Tania F. Gendron

**Affiliations:** 1 Department of Neuroscience, Mayo Clinic, 4500 San Pablo Road, Jacksonville, FL 32224 USA; 2 Mayo Clinic Graduate School of Biomedical Sciences, Mayo Clinic, 4500 San Pablo Road, Jacksonville, FL 32224 USA; 3 Division of Clinical Trials and Biostatistics, Mayo Clinic, 4500 San Pablo Road, Jacksonville, FL 32224 USA; 4 Memory and Aging Center, Department of Neurology, University of California San Francisco, 675 Nelson Rising Lane, San Francisco, CA 91358 USA; 5 Department of Neurology, Mayo Clinic, 200 First St, SW, Rochester, MN 55905 USA; 6 Department of Medical and Molecular Genetics, The National Centralized Repository for Alzheimer’s Disease and Related Dementias, 351 W. 10Th St TK-217, Indianapolis, IN 46202 USA; 7 Department of Biomedical Sciences, University of Antwerp, Universiteitsplein 1, 2610 Antwerp, Belgium; 8 VIB Center for Molecular Neurology, VIB, Universiteitsplein 1, 2610 Antwerp, Belgium; 9 Department of Neurology, David Geffen School of Medicine, University of California Los Angeles, Reed Neurological Research Center, 710 Westwood Plaza, Los Angeles, CA 90095 USA; 10 Department of Neurology, Case Western Reserve University, 11100 Euclid Ave, Cleveland, OH 44106 USA; 11 Department of Neurology, University of North Carolina, 170 Manning Dr, Chapel Hill, NC 27599 USA; 12 Department of Neurology, Vanderbilt University, 1161 21St Ave S, Nashville, TN 37212 USA; 13 Department of Neurology, Frontotemporal Disorders Unit, Massachusetts General Hospital and Harvard Medical School, 149 13th St, Boston, MA 02129 USA; 14 Department of Neurology, University of Washington, 1959 NE Pacific St, Seattle, WA 98195-6465 USA; 15 Department of Neurosciences, University of California, 9500 Gilman Drive, La Jolla, CA 92037-0948 USA; 16 Departments of Neurology and Psychiatry, Washington University School of Medicine, Washington University, 660 South Euclid, St. Louis, MO 63110 USA; 17 Department of Neurology, Mayo Clinic, 4500 San Pablo Road, Jacksonville, FL 32224 USA; 18 Department of Neurology, Mesulam Center for Cognitive Neurology and Alzheimer’s Disease, Northwestern Feinberg School of Medicine, 300 E. Superior, Tarry 8-715, Chicago, IL 60610 USA; 19 Center for Neurodegenerative Disease, Department of Neurology, Emory University School of Medicine and Emory, 12 Executive Park Drive, Atlanta, GA 30329 USA; 20 Division of Neurology, University of British Columbia, S151-2211 Wesbrook Mall, Vancouver, BC V6T 2B5 Canada; 21 Department of Psychiatry and Human Behavior, Alpert Medical School of Brown University, 345 Blackstone Boulevard, Providence, RI 02906 USA; 22 Department of Neurology and Penn Frontotemporal Degeneration Center, Perelman School of Medicine, University of Pennsylvania, 3400 Spruce St, Philadelphia, PA 19104 USA; 23 Disorders and Stroke, National Institute of Neurological, National Institutes of Health, 10 Center Drive, Bethesda, MD 20892 USA; 24 Department of Neurosciences, University of California, 9452 Medical Center Drive, La Jolla, CA 92037 USA; 25 Department of Pathology and Laboratory Medicine, University of British Columbia, 2211 Wesbrook Mall, Vancouver, BC V6T 2B5 Canada; 26 Department of Neurology, Houston Methodist Neurological Institute, Weill Cornell Medicine, 6560 Fannin St, Houston, TX 77030 USA; 27 Department of Psychiatry and Behavioral Sciences, Johns Hopkins University School of Medicine, 600 North Wolfe Street, Baltimore, MD 21287 USA; 28 Department of Neurology, University of Colorado School of Medicine, 12631 East 17Th Avenue, Aurora, CO 80045 USA; 29 Layton Aging and Alzheimer’s Disease Research Center, Oregon Health and Science University, 3181 SW Sam Jackson Park Road, Portland, OR 97239 USA; 30 Department of Neurology, University of Alabama at Birmingham, 1825 University Blvd, Birmingham, AL 35233 USA; 31 Division of Neurology, Tanz Centre for Research in Neurodegenerative Diseases, University of Toronto, 6 Queen’s Park Crescent West, Third Floor, Toronto, ON M5S 3H2 Canada; 32 Department of Pathology, University of California, San Francisco, 505 Parnassus Avenue, San Francisco, CA 94143 USA

**Keywords:** Behavioral variant frontotemporal dementia, Biofluid, Biomarker, Corticobasal syndrome, Glial fibrillary acidic protein, Neurofilament light, Plasma, Primary progressive aphasia, Presymptomatic, Progressive supranuclear palsy-Richardson’s syndrome

## Abstract

**Background:**

Therapeutic development for frontotemporal dementia (FTD) is hindered by the lack of biomarkers that inform susceptibility/risk, prognosis, and the underlying causative pathology. Blood glial fibrillary acidic protein (GFAP) has garnered attention as a FTD biomarker. However, investigations of GFAP in FTD have been hampered by symptomatic and histopathologic heterogeneity and small cohort sizes contributing to inconsistent findings. Therefore, we evaluated plasma GFAP as a FTD biomarker and compared its performance to that of neurofilament light (NfL) protein, a leading FTD biomarker.

**Methods:**

We availed ARTFL LEFFTDS Longitudinal Frontotemporal Lobar Degeneration (ALLFTD) study resources to conduct a comprehensive cross-sectional and longitudinal examination of the susceptibility/risk, prognostic, and predictive performance of GFAP and NfL in the largest series of well-characterized presymptomatic FTD mutation carriers and participants with sporadic or familial FTD syndromes. Utilizing single molecule array technology, we measured GFAP and NfL in plasma from 161 controls, 127 presymptomatic mutation carriers, 702 participants with a FTD syndrome, and 67 participants with mild behavioral and/or cognitive changes. We used multivariable linear regression and Cox proportional hazard models adjusted for co-variates to examine the biomarker utility of baseline GFAP and NfL concentrations or their rates of change.

**Results:**

Compared to controls, GFAP and NfL were elevated in each FTD syndrome but GFAP, unlike NfL, poorly discriminated controls from participants with mild symptoms. Similarly, both baseline GFAP and NfL were higher in presymptomatic mutation carriers who later phenoconverted, but NfL better distinguished non-converters from phenoconverters. We additionally observed that GFAP and NfL were associated with disease severity indicators and survival, but NfL far outperformed GFAP. Nevertheless, we validated findings that the GFAP/NfL ratio may discriminate frontotemporal lobar degeneration with tau versus TDP-43 pathology.

**Conclusions:**

Our head-to-head comparison of plasma GFAP and NfL as biomarkers for FTD indicate that NfL consistently outmatched GFAP as a prognostic and predictive biomarker for participants with a FTD syndrome, and as a susceptibility/risk biomarker for people at genetic risk of FTD. Our findings underscore the need to include leading biomarkers in investigations evaluating new biomarkers if the field is to fully ascertain their performance and clinical value.

**Supplementary Information:**

The online version contains supplementary material available at 10.1186/s13024-025-00821-4.

## Background

Frontotemporal dementia (FTD), an umbrella term for a group of clinical syndromes marked by progressive behavior, language, executive function and/or motor impairments, is a leading cause of dementia in individuals under the age of 65 years [1, 2]. While FTD syndromes share symptoms, behavioral variant FTD (bvFTD), the most common FTD syndrome, typically presents with a change in personality or behavior associated with executive dysfunction. The nonfluent/agrammatic variant of primary progressive aphasia (nfvPPA) is characterized by nonfluent speech, agrammatism and phonemic errors, while semantic variant PPA (svPPA) is characterized by single-word comprehension and naming deficits [3]. The parkinsonian disorders, corticobasal syndrome (CBS) and progressive supranuclear palsy-Richardson syndrome (PSP-RS), as well as bvFTD with amyotrophic lateral sclerosis (FTD-ALS), are also included among the FTD spectrum disorders.

Whereas FTD refers to clinical syndromes, the term frontotemporal lobar degeneration (FTLD) refers to the underlying neurodegenerative pathologies. The most common FTLD pathologic types are FTLD-TDP and FTLD-tau, which are respectively characterized by misfolded TAR DNA-binding protein 43 (TDP-43) and tau proteins. Some FTD syndromes strongly associate with FTLD-tau (i.e., PSP-RS) or FTLD-TDP (i.e., FTD-ALS) [4], but bvFTD is just as likely to be caused by either proteinopathy (with a small percentage of cases caused by rarer FTLD pathologies). Consequently, other than individuals with familial FTD caused by gene mutations giving rise to TDP-43 (*C9orf72*, *GRN* or *TARDBP*) or tau (*MAPT*) pathology, it is difficult to definitively determine, during life, the underlying neuropathology of most FTD syndromes, and presently impossible to do so for individuals with sporadic bvFTD.

Given the clinical, genetic and neuropathological heterogeneity within and among FTD syndromes, the successful development of FTD treatments hinges on identifying biomarkers that facilitate an early and accurate diagnosis, predict phenoconversion for individuals at genetic risk, inform prognosis, classify FTLD pathological subtypes, and monitor the effects of novel interventions in clinical trials. No single biomarker will fulfill all these needs. However, we previously evaluated plasma neurofilament light (NfL) protein, a marker of neuronal injury, across FTD spectrum disorders and in presymptomatic FTD mutation carriers; we found that NfL was elevated in presymptomatic mutation carriers prior to phenoconversion and in participants with FTD, and it associated with indicators of disease severity [5].

Given findings that glial fibrillary acidic protein (GFAP), an astrocytic cytoskeletal protein, shows promise as a biomarker for traumatic brain injury (TBI) [6], Alzheimer disease [7] and other diseases, we examined the potential utility of GFAP as a biomarker for FTD. Indeed, some studies reported elevated blood GFAP in participants with FTD compared to controls [8–18] but others observed no such GFAP increase in FTD [19–22]. Similarly, some investigators [8, 11, 14] but not others [9, 21–23] found that blood GFAP concentrations correlate with clinical markers of disease severity in participants with FTD. Small cohort sizes and the heterogeneity of FTD spectrum disorders may underlie these discrepancies. For instance, grouping participants with different FTD syndromes may increase statistical power but possibly at the expense of masking associations of interest or incorrectly ascribing findings to a particular FTD syndrome. Because of these limitations and the relative dearth of studies investigating GFAP in FTD, whether blood GFAP could be a useful FTD biomarker remains unknown.

Establishing the putative utility of blood GFAP as a FTD biomarker requires rigorous investigations utilizing large cohorts representing all FTD syndromes, and the comparison of GFAP to more validated biomarkers, such as NfL. We thus used ARTFL LEFFTDS Longitudinal Frontotemporal Lobar Degeneration (ALLFTD; www.allftd.org) resources and conducted a comprehensive cross-sectional and longitudinal study to evaluate and compare the prognostic, susceptibility/risk and predictive performance of plasma GFAP and NfL in the largest series of well-characterized presymptomatic FTD mutation carriers and participants with FTD syndromes.

## Methods

### Study participants

The aim of this study was to determine whether plasma GFAP is a reliable biomarker for FTD, and to compare its biomarker utility to that of plasma NfL. To do so, we used plasma from participants enrolled through either or both North American multicenter observational studies: Advancing Research and Treatment for Frontotemporal Lobar Degeneration (ARTFL, NCT02365922), and Longitudinal Evaluation of Familial Frontotemporal Dementia Subjects (LEFFTDS, NCT02372773) [24], now combined into the ARTFL-LEFFTDS Longitudinal Frontotemporal Lobar Degeneration (ALLFTD, NCT04363684) study.

Human participant characteristics, which include age at baseline, sex assigned at birth, age at symptom onset, symptom duration (time between symptom onset and plasma collection), body mass index (BMI), mutation status, years of education, age at death, and neuropathological diagnosis are provided in Table 1. Data for some characteristics, in particular BMI, were missing for some participants and are reported in Table 1. All diagnoses were made clinically using widely accepted published criteria for each disorder [25, 26]. The 1,057 participants in this study are comprised of 161 clinically normal, mutation-negative individuals from kindreds with known FTD-related gene mutations, 127 asymptomatic individuals with an FTD-causing mutation (presymptomatic mutation carriers), 308 participants with bvFTD, 76 with nfvPPA, 83 with svPPA, 92 with CBS, 143 with PSP-RS, and 67 with mild behavioral and/or cognitive impairments (MBCI). Within the MBCI group, 49 individuals were classified as having mild cognitive impairment, and 18 were classified as having mild behavioral changes. Participants clinically diagnosed with FTD-ALS were not included in this study.

**Table 1:** Participant characteristics according to phenotypic groups

	Median (minimum, maximum) or No. (%) of participants							
Variable	Controls (*N* = 161)	PreSx (*N* = 127)	bvFTD (*N* = 308)	nfvPPA (*N* = 76)	svPPA (*N* = 83)	CBS (*N* = 92)	PSP-RS (*N* = 143)	MBCI (*N* = 67)
Age at baseline (years)	53 (40, 80)	49 (40, 80)	62 (32, 85)	70 (49, 86)	66 (50, 88)	68 (40, 87)	69 (49, 82)	60 (30, 82)
Sex (Male)	55 (34.2%)	64 (50.4%)	181 (58.8%)	34 (44.7%)	42 (50.6%)	48 (52.2%)	75 (52.4%)	34 (50.7%)
Age at symptom onset (years)	N/A	N/A	58 (26, 80)	65 (44, 81)	60 (38, 81)	68 (40, 87)	69 (49, 82)	60 (30, 82)
Unknown	N/A	N/A	2	0	1	0	0	0
Symptom duration (years)	N/A	N/A	4 (0, 32)	4 (1, 12)	5 (1, 17)	4 (0, 32)	5 (1, 20)	2 (0, 54)
Unknown	N/A	N/A	2	0	1	0	14	2
BMI	27.8 (18.3, 53.2)	25.9 (16.5, 42.3)	27.5 (12.6, 58.7)	26.0 (12.4, 35.0)	25.7 (16.6, 39.7)	26.1 (17.5, 40.5)	25.8 (17.7, 41.8	27.9 (18.8, 39.2)
Unknown	36	18	47	13	12	11	29	23
Mutation status								
None	161 (100.0%)	0 (0.0%)	189 (61.4%)	69 (90.8%)	78 (94.0%)	79 (85.9%)	134 (93.7%)	33 (49.3%)
*C9orf72*	0 (0.0%)	60 (47.2%)	56 (18.2%)	0 (0.0%)	1 (1.2%)	2 (2.2%)	1 (0.7%)	15 (22.4%)
*GRN*	0 (0.0%)	34 (26.8%)	21 (6.8%)	6 (7.9%)	1 (1.2%)	6 (6.5%)	0 (0.0%)	7 (10.4%)
*MAPT*	0 (0.0%)	31 (24.4%)	32 (10.4%)	0 (0.0%)	1 (1.2%)	1 (1.1%)	1 (0.7%)	11 (16.4%)
*C9orf72* and *GRN*	0 (0.0%)	2 (1.6%)	0 (0.0%)	0 (0.0%)	0 (0.0%)	0 (0.0%)	0 (0.0%)	0 (0.0%)
Other	0 (0.0%)	0 (0.0%)	3 (1.0%)	1 (1.3%)	1 (1.2%)	1 (1.1%)	0 (0.0%)	0 (0.0%)
Unknown	0 (0.0%)	0 (0.0%)	7 (2.3%)	0 (0.0%)	1 (1.2%)	3 (3.3%)	7 (4.9%)	1 (1.5%)
Years of education	16 (12, 22)	16 (10, 26)	16 (6, 26)	16 (10, 24)	16 (12, 21)	16 (5, 26)	16 (12, 24)	16 (9, 20)
CDR + NACC-FTLD global score								
0	160 (100%)	125 (100%)	0 (0.0%)	0 (0.0%)	0 (0.0%)	2 (2.2%)	2 (1.6%)	1 (1.5%)
0.5	0 (0.0%)	0 (0.0%)	18 (5.9%)	31 (40.8%)	11 (13.4%)	24 (26.1%)	21 (16.8%)	65 (97.0%)
1	0 (0.0%)	0 (0.0%)	103 (33.8%)	31 (40.8%)	48 (58.5%)	40 (43.5%)	50 (40.0%)	0 (0.0%)
2	0 (0.0%)	0 (0.0%)	156 (51.1%)	12 (15.8%)	22 (26.8%)	22 (23.9%)	42 (33.6%)	1 (1.5%)
3	0 (0.0%)	0 (0.0%)	28 (9.2%)	2 (2.6%)	1 (1.2%)	4 (4.3%)	10 (8.0%)	0 (0.0%)
Follow-up after baseline GFAP and NfL measurement	N/A	N/A	301 (97.7%)	76 (100.0%)	82 (98.8%)	90 (97.8%)	129 (90.2%)	N/A
Death	N/A	N/A	42 (14.0%)	16 (21.1%)	13 (15.9%)	14 (15.6%)	20 (15.5%)	N/A
Age at death (median; range)	N/A	N/A	66 (41, 79)	71 (61, 80)	68 (57.0, 81)	74 (59, 88)	73 (57, 83)	N/A
Neuropathological assessment	N/A	N/A	42 (13.6%)	16 (21.1%)	13 (15.7%)	14 (15.2%)	22 (15.4%)	N/A
Tau pathology	N/A	N/A	20 (6.5%)	15 (19.7%)	3 (3.6%)	11 (12.0%)	22 (15.4%)	N/A
TDP-43 pathology	N/A	N/A	22 (7.1%)	1 (1.3%)	10 (12.0%)	3 (3.3%)	0 (0.0%)	N/A

*BMI* Body mass index, *bvFTD* behavioral variant frontotemporal dementia, *CBS* Corticobasal syndrome, *CDR® + NACC-FTLD global score* CDR® Dementia Staging Instrument plus behavior and language domains from the National Alzheimer’s Disease Coordinating Center FTLD module global score, *MBCI* Mild behavioral and/or cognitive impairments, *nfvPPA* nonfluent/agrammatic variant of primary progressive aphasia, *PreSx* Presymptomatic, *PSP-RS* Progressive supranuclear palsy-Richardson syndrome, *svPPA* semantic variant primary progressive aphasia

### Genetic testing

Genetic testing for study participants was performed at the University of California, Los Angeles as previously described [27]. In brief, using targeted sequencing or whole-genome sequencing, DNA samples were screened for genes implicated in neurodegenerative diseases (e.g., *APP*, *ATNX2*, *CHMP2B, FUS*, *GRN*, *MAPT*, *SQSTM1*, *TARDBP, TBK1*, *TIA1*, *UBQLN1*, *VCP*). Hexanucleotide repeat expansions in *C9orf72* were detected using both fluorescent and repeat-primed PCR. Asymptomatic participants who completed clinical genetic testing and who had no mutations in the screened genes were designated as controls.

### Participant relatedness

Genome-wide single nucleotide polymorphism genotyping data were used to infer familial relatedness, as previously described [28]. Participant relatedness within each phenotype group, and also in relation to controls and presymptomatic mutation carriers, is summarized in Tables S1, S2 and S3. Relatedness within phenotype groups occurred most commonly for controls (32.9% participants from families), presymptomatic mutation carriers (36.2% participants from families), and participants with MBCI (11.9% participants from families), with a maximum of 3.9% subjects from families in the remaining phenotype groups (Table S1). When examining relatedness to the control group, this occurred most frequently in presymptomatic mutation carriers (61.4% participants related to controls), participants with MBCI (43.2% participants related to controls), and participants with bvFTD (13.0% participants related to controls), with a maximum of 3.3% participants related to controls in other phenotype groups (Table S2). Relatedness to presymptomatic mutation carriers occurred almost exclusively in participants with MBCI (28.3%) or bvFTD (11.7%) (Table S3).

### Clinical procedures

Study participants recruited through ALLFTD underwent annual standardized evaluations including participant and caregiver interviews, neurological assessments, and neuropsychological testing. Clinical testing included measures of clinical severity, such as the CDR® Dementia Staging Instrument plus behavior and language domains from the National Alzheimer’s Disease Coordinating Center Frontotemporal Lobar Degeneration module (CDR® + NACC-FTLD) [29], and the following neuropsychological tests:

#### Montreal Cognitive Assessment (MoCA)

This 30-point cognitive screening tool evaluates visuospatial, semantic, phonemic and fluent language, working memory, recall, attention, and orientation [30].

#### Northwestern Anagram Test (NAT)

For this measure of sentence production, individuals are asked to organize words into ten grammatically correct sentences accurately describing a picture stimulus, thus allowing the detection of grammatic deficits independently of speech production, word-finding impairments and working memory capacity [31].

#### The Multilingual Naming Test (MINT)

This test detects naming deficits by asking participants to name 32 black and white line-drawn items. The total score includes items named correctly with semantic, but not phonemic, cues [32].

#### Verbal semantic/category test

In two 60-s trials, this test requires participants to produce as many words as possible belonging to “animal” or “vegetable” categories. The number of correct words across both trials represents the final score.

#### Verbal fluency phonemic test

In two 60-s trials, this test requires participants to produce as many words as possible that begin with the letter “F” or “L”. The number of correct words across both trials represents the final score.

#### Digit span backward

Participants are read a sequence of numbers that become increasingly longer and must repeat the sequence in reverse order.

#### Trail Making Test Part B (Trails B)

This executive function test consists of 24 circles on a piece of paper; 12 circles having the numbers 1 to 12, 12 circles with the letters A to L. Participants are tasked with drawing a line from one circle to the next in ascending order, alternating between numbers and letters [33].

#### Plasma GFAP and NfL concentration determination

Participant blood was collected in ethylene diamine tetra-acetic acid (EDTA) tubes and centrifuged at 1,500 g at 4 °C for 15 min. The resulting plasma was aliquoted and stored at -80 °C at the National Centralized Repository for Alzheimer’s Disease and Related Dementias (NCRAD), and aliquots were shipped to the Mayo Clinic in Jacksonville, FL. For the above-mentioned 1,057 participants in our study (Table 1), baseline and longitudinal plasma GFAP concentrations were measured using the GFAP discovery digital immunoassay (Quanterix, Cat# 102,336, Lot #503,909). Baseline and longitudinal NfL concentrations from study participants were measured using the NF-Light digital immunoassay (Quanterix, Cat#103,186) using two separate kit lots; NfL was measured in 1,061 plasma samples using Lot #501,992, and 630 plasma samples using Lot #503,729. We also measured NfL in additional distinct plasma samples using both Lot #501,992 and Lot #503,729 kits, which served as calibrators for inter-assay normalization. Overall, across the 1,057 study participants, matching baseline and longitudinal GFAP and NfL measures were available for 1,691 samples. GFAP and NfL were measured in a blinded manner using the same HD-X Analyzer per the manufacturer’s protocol. In brief, samples were thawed on ice, mixed thoroughly by low-speed vortexing and centrifuged at 4 °C at 10,000 g for five min before transferring samples to 96-well plates. Samples were diluted 1:4 by the instrument and tested in duplicate. In addition to participant plasma samples, each run included eight calibrators and two quality control samples provided with the kits, as well as a pooled reference sample provided by NCRAD. When the concentration of GFAP or NfL in a sample exceeded the upper limit of the calibration curve, the sample was retested following an at-bench dilution. Concentrations were interpolated from the standard curve using a 4-parameter logistic curve fit (1/y^2^ weighted).

### Statistical analysis

#### General information

Statistical analyses were performed using SAS (version 9.4; SAS Institute, Inc., Cary, North Carolina) and all statistical tests were two-sided. Continuous variables were summarized with sample median and range. Categorical variables were summarized with participant number and percentage. Baseline GFAP and NfL concentrations, and the GFAP/NfL ratio were examined on the base-2 logarithm scale in all regression analyses due to their skewed distributions. All co-variates adjusted for in multivariable regression models were pre-defined. A Bonferroni correction for multiple testing was applied separately for each biomarker for each group of similar statistical tests (see specific statistical analysis sections below for details on statistical significance levels for a given analysis).

#### Correlations of baseline plasma GFAP and NfL

Correlations between baseline GFAP and NfL were assessed using Spearman’s test of correlation separately for ten different groups, where *p* < 0.005 was considered statistically significant.

#### Associations of plasma biomarkers with age, sex, symptom duration, and BMI

Separately for each phenotype group, associations of baseline GFAP and NfL with age, sex, symptom duration (for symptomatic participants only), and BMI were assessed using unadjusted and multivariable linear regression models. Multivariable models were adjusted for age and sex, and also for symptom duration for analysis of symptomatic participants only. We did not adjust for BMI in this or other subsequently mentioned multivariable analysis due to the aforementioned missing BMI data. β coefficients and 95% confidence intervals (CIs) were estimated and are interpreted as the change in mean GFAP or NfL concentration corresponding to a specified increase (age, symptom duration, BMI) or for females in comparison to males. *p* < 0.0167 (controls and presymptomatic mutation carriers) or *p* < 0.0125 (all symptomatic groups) are considered statistically significant after correcting for multiple testing separately for each phenotype group.

#### Comparisons of baseline biomarker concentrations among phenotype groups

Comparisons of baseline GFAP and NfL concentration between controls and seven phenotype groups, and between presymptomatic mutation carriers and six phenotype groups were made using unadjusted and age/sex-adjusted linear regression models. These linear regression models were used when making these same comparisons of baseline GFAP and NfL between controls or presymptomatic mutation carriers and phenotypes groups for individuals with a CDR® + NACC-FTLD global score of 0 or 0.5, and when stratifying by symptom duration. β coefficients and 95% CIs were estimated and are interpreted as the difference in mean GFAP or NfL concentration (on the base-2 logarithm scale) between the two groups of interest. *p* < 0.0071 (controls vs. phenotype groups) and *p* < 0.0083 (presymptomatic mutation carriers vs. phenotype groups and controls vs. phenotype groups when stratifying by disease duration) were considered statistically significant after correcting for multiple comparisons. Linear regression models adjusted for age, sex and symptom duration were used to compare GFAP and NfL concentration among the six symptomatic groups where *p* < 0.0033 was statistically significant after correcting for the 15 pair-wise comparisons.

Linear regression models adjusted for age and sex were used to compare baseline GFAP and NfL between: 1) controls and presymptomatic mutation carriers who did or did not phenoconvert; and 2) non-converters and phenoconverters; *p* < 0.0167 was considered statistically significant after correcting for these three comparisons. These same linear regression models were used to compare baseline GFAP and NfL between controls and presymptomatic individuals with either a *C9orf72*, *GRN* or *MAPT* mutation where *p* < 0.0167 was considered as significant following three comparisons vs. controls. β coefficients and 95% CIs were estimated and are interpreted as previously described.

#### Determination of the discriminatory power of baseline biomarkers

To assess the ability of baseline GFAP and NfL to discriminate between controls and phenotype groups, and between presymptomatic mutation carriers and phenotype groups, we estimated the area under receiver operating characteristic curve (AUC) values along with 95% CIs, where an AUC equal to 0.5 represents no discriminatory ability and an AUC equal to 1.0 represents perfect discrimination. Unadjusted AUCs were initially estimated, however, since age and sex differed among groups and were also associated with baseline GFAP and NfL concentrations, these AUC estimates were influenced by age and sex and were consequently biased. Therefore, for participants in a given comparison, we calculated age and sex-adjusted AUC estimates by first extracting the residuals from a linear regression model where baseline GFAP or NfL was the dependent variable and both age and sex were independent variables, thereby essentially normalizing by age and sex. We then compared these model residuals between the two groups of interest to estimate AUC values for a given pair-wise comparison in our adjusted analyses. When assessing the combined ability of both GFAP and NfL to discriminate between groups, age and sex-adjusted residuals were first extracted as previously described, and AUC values were estimated from a logistic regression model were these residuals for both GFAP and NfL were included as covariates and the outcome was the given dichotomous phenotype variable.

#### Determination of rates of change in biomarker concentrations and in disease severity indicators

For participants having longitudinal GFAP and NfL measurements and for whom the baseline and last measurements were at least one year apart, we estimated, separately for each participant, the rate of change in GFAP and NfL per year by extracting the β coefficient from a linear regression model where GFAP or NfL was the dependent variable and time since initial GFAP or NfL measure was the independent variable (logarithm transformations of GFAP and NfL were not used in these analyses). This same strategy was applied when calculating rate of change in disease indicators per year. These rates of change were then utilized in subsequently described statistical analyses.

#### Comparisons of rates of change in GFAP and NfL concentrations among phenotype groups

Due to the combination of: 1) skewness and presence of outliers in rates of GFAP and NfL change per year, and 2) small sample sizes, we utilized a rank transformation of rates of change per year in GFAP and NfL concentrations in all regression analyses to minimize outlier impact on the results [34].

Using linear regression models adjusted for age and sex, we compared rate of change per year in GFAP and NfL concentrations between: 1) controls and phenoconverters or non-converters, and 2) phenoconverters and non-converters, where *p* < 0.0167 is considered statistically significant after correcting for three pair-wise comparisons. Age and sex-adjusted linear regression models were also used to compare rate of change per year in GFAP and NfL concentrations between controls and presymptomatic individuals with either a *C9orf72*, *GRN* or *MAPT* mutation; *p* < 0.0167 was again considered as significant following the three comparisons vs. controls. β coefficients and 95% confidence CIs were estimated and are interpreted as the difference in the mean rank of rate of change in GFAP or NfL concentration per year between the two groups being compared.

We used linear regression models adjusted for age, sex and symptom duration to compare rate of change per year in GFAP and NfL concentrations between controls and other phenotype groups. *p* < 0.0071 is considered statistically significant after correcting for six comparisons vs. controls.

#### Associations of baseline biomarkers with indicators of disease severity

Associations of baseline GFAP and NfL with baseline disease indicators were examined using linear regression models, separately in each phenotype group and in the combined FTD group. Unadjusted models and multivariable models adjusted for age, sex, symptom duration and years of education were evaluated. β coefficients and 95% CIs were estimated and interpreted as the change in the mean outcome measure for each doubling in GFAP or NfL concentration. *p* < 0.00625 was considered statistically significant after correcting for the eight tests of association that were performed in each phenotype group.

Similarly, linear regression models were employed to evaluate associations of baseline biomarkers with rate of change per year in disease indicators for the combined group of participants with bvFTD, nfvPPA, svPPA, CBS or PSP-RS. *p* < 0.0083 was considered statistically significant after correcting for multiple comparisons.

#### Associations of baseline biomarkers with survival after symptom onset

Associations of baseline GFAP and NfL with survival after symptom onset were examined using Cox proportional hazards regression models. Censoring occurred at the age of the last known follow-up. Unadjusted models were examined separately for each FTD syndrome and for all FTD syndromes combined. Multivariable models adjusted for age at symptom onset, sex, and symptom duration were assessed only for the bvFTD group and the combined group of FTD syndromes owing to the much smaller number of deaths in the other phenotype groups. Hazard ratios (HRs) and 95% CIs were estimated and correspond to each doubling in GFAP or NfL concentration. To further assess the ability of baseline GFAP and NfL to predict survival after symptom onset, c-index was estimated both with and without GFAP or NfL in the given multivariable model, where a c-index of 1.0 indicates perfect predictive ability and a value of 0.5 indicates predictive ability equal to chance. *p* < 0.05 was considered as statistically significant given that only one test of association was performed in each phenotype group for each biomarker.

#### Associations of baseline GFAP, NfL and GFAP/NfL according to mutation status in the combined group of participants with FTD syndromes

Unadjusted linear regression models and models adjusted for age, sex and symptom duration were used to compare, in the combined group of participants with an FTD syndrome, baseline GFAP, NfL or the GFAP/NfL ratio between: 1) non-mutation carriers and *C9orf72*, *GRN* or *MAPT* mutation carriers; and 2) *C9orf72*, *GRN* or *MAPT* mutation carriers. *p* < 0.0083 is considered as statistically significant after correcting for six pair-wise comparisons.

#### Associations of baseline GFAP and NfL with underlying pathology

Of the participants clinically diagnosed with an FTD syndrome (bvFTD, nfvPPA, svPPA, CBS or PSP-RS), 71 were neuropathologically diagnosed with FTLD-tau (18 with Pick’s disease, 26 with PSP, and 27 with corticobasal degeneration), and 41 were neuropathologically diagnosed with FTLD-TDP. Of these 41 individuals with FTLD-TDP, 17 had FTLD-TDP type A, 9 had FTLD-TDP type C, and 15 had FTLD-TDP of unknown subtype. Individuals with a FTLD-TDP type B neuropathological diagnosis were not included on the basis that TDP-43 type B is associated with FTD-ALS phenotypes [35], and that participants with ALS have significantly higher plasma NfL than participants with FTD, which may confound our analyses [5].

We compared baseline GFAP, NfL and the GFAP/NfL ratio between participants with tau or TDP-43 pathology using linear regression models adjusted for age at biomarker measurement, sex, symptom duration, and age of death. β coefficients and 95% CIs were estimated and are interpreted as the difference in the mean GFAP, NfL, or GFAP/NfL concentration (on the base-2 logarithm scale) between the FTLD-tau and FTLD-TDP-43 groups.

#### Sensitivity analyses

We performed two different sensitivity analyses. First, although it can be reasonably argued that participants within a given family can be treated as independent in the context of this study (as we have done for our primary analysis), it would also be reasonable to directly address any possible within-family correlation in our statistical analysis. Therefore, in secondary analyses, aforementioned regression analyses were redone using either generalized estimating equations (GEE) (for linear regression models) [36], or using a marginal models approach (for Cox regression models) [37] to account for familial relatedness. Second, although we did not adjust our primary multivariable models for BMI due to the non-negligible amount of missing BMI data, in secondary analysis we did assess our previously described multivariable models with additional adjustment for BMI.

## Results

### Participant characteristics

Using plasma obtained at baseline and follow-up visits, we measured GFAP and NfL in plasma from clinically normal, mutation-negative individuals from kindreds with an FTD-causing mutation (controls, *N* = 161), presymptomatic individuals with a *C9orf72* repeat expansion or a *GRN* or *MAPT* mutation (*N* = 127), and participants with sporadic or genetic bvFTD (*N* = 308), nfvPPA (*N* = 76), svPPA (*N* = 83), CBS (*N* = 92) or PSP-RS (*N* = 143). Participants with MBCI (*N* = 67) were included for comparison in some analyses [38]. Demographic and clinical data are presented in Table 1. Table S4 indicates the number of controls, presymptomatic phenoconverters, non-converters and other phenotype groups with baseline and longitudinal GFAP and NfL measures, and for whom rates of change of these biomarkers could be determined.

First, we evaluated correlations of baseline GFAP and NfL concentrations, finding a moderate to strong, and highly statistically significant correlation in all participants combined (Spearman’s r: 0.61, *p* < 2.2E-16) (Table S5). When examining each phenotype group separately, correlations were weakest for PSP-RS (Spearman’s r: 0.28, *p* = 6.6E-04), CBS (Spearman’s r: 0.31, *p* = 0.003), and svPPA (Spearman’s r: 0.34, *p* = 0.002), and strongest for MBCI (Spearman’s r: 0.62, *p* = 6.5E-08), controls (Spearman’s r: 0.59, *p* < 2.2E-16), and presymptomatic mutation carriers (Spearman’s r: 0.52, *p* < 3.6E-10).

**Fig. 1:**
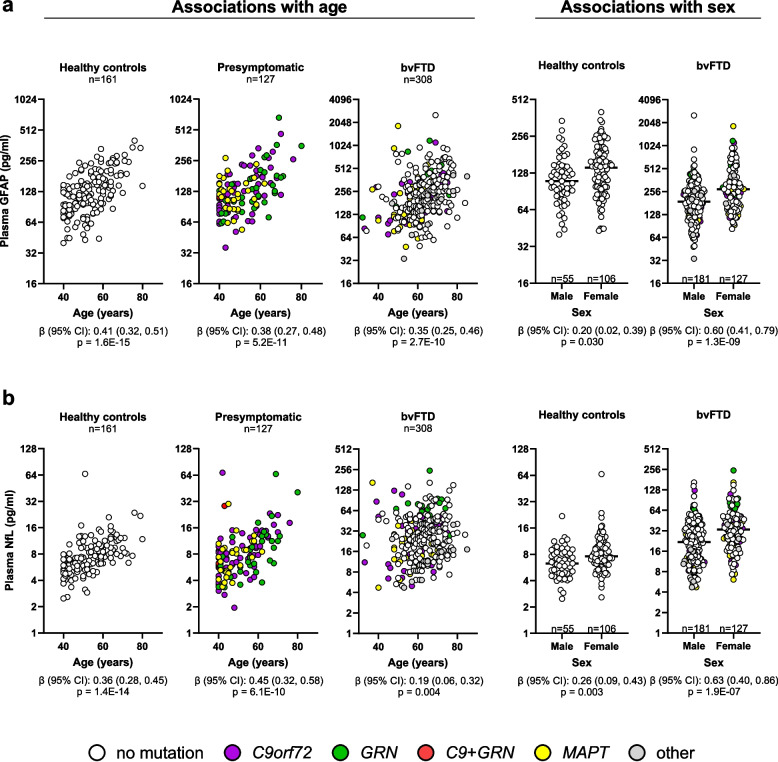
Associations of plasma GFAP and NfL concentrations with age and sex in some phenotype groups. Associations of baseline concentrations of GFAP (**a**) or NfL (**b**) with age and sex assessed using linear regression models adjusted for age, sex and, for bvFTD, also for symptom duration. The number of participants (n), β coefficients (β), 95% confidence intervals (95% CI) and p values are shown. *p* < 0.025 (controls and presymptomatic mutation carriers) or *p* < 0.017 (bvFTD) are considered statistically significant. Grey circles represent seven bvFTD participants with an unknown mutation status, two bvFTD participants with a *TARDBP* mutation, and one bvFTD participant with a likely *VCP* pathogenic variant. GFAP and NfL concentrations are shown on the base 2 logarithm scale. Black horizontal bars represent median GFAP or NfL concentrations. See also Tables S6 and S7 for associations of baseline GFAP and NfL with age, sex and disease duration in other disease groups

To identify potential confounding variables that influence GFAP or NfL concentrations in each phenotype group, we examined associations of baseline biomarkers with age, sex, symptom duration (time between symptom onset and plasma collection), and BMI in unadjusted analyses (Table S6), and analyses adjusting for age, sex and, when only symptomatic groups were included, symptom duration (Table S7). In adjusted analysis, increased GFAP concentration was associated with older age in controls (*p* = 1.6E-15), presymptomatic mutation carriers (*p* = 5.2E-11) and participants with bvFTD (*p* = 2.7E-10), svPPA (*p* = 3.0E-05), PSP-RS (*p* = 0.005) or MBCI (*p* = 1.5E-06), with female sex in participants with bvFTD (*p* = 1.3E-09) or nfvPPA (*p* = 0.005), and with a higher BMI in participants with PSP-RS only (*p* = 0.0012) (Fig. 1a, Table S7). GFAP was not associated with symptom duration in any symptomatic group. In adjusted analyses, higher NfL was significantly associated with older age in controls (*p* = 1.4E-14), presymptomatic mutation carriers (*p* = 6.1E-10) and participants with bvFTD (*p* = 0.004), CBS (*p* = 0.004) or MBCI (*p* = 8.2E-04), with female sex in controls (*p* = 0.003) and participants with bvFTD (*p* = 1.9E-07), with shorter symptom duration in participants with bvFTD (*p* = 0.004), and with higher BMI in participants with bvFTD only (*p* = 0.0018) (Fig. 1b, Table S7). Accordingly, below we discuss results from analyses adjusted for age and sex and, when appropriate, other potential confounders (e.g., symptom duration, education, age at death). Data from both unadjusted and adjusted analyses are provided in supplemental tables.

### Plasma GFAP is elevated in FTD syndromes

**Fig. 2:**
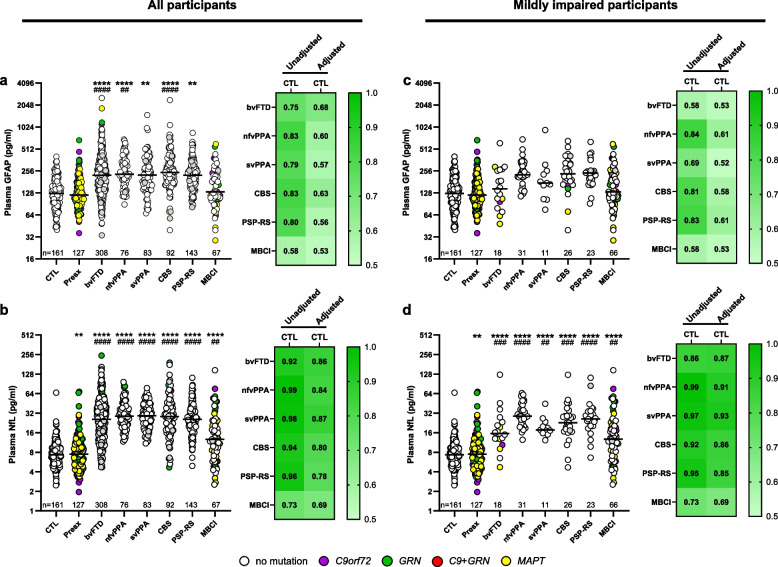
Baseline plasma GFAP and NfL are elevated in FTD syndromes. **a, b** Comparison of baseline plasma GFAP (**a**) and NfL (**b**) between healthy controls or presymptomatic mutation carriers and all participants for a given symptomatic group. **c, d** Comparison of baseline GFAP (**c**) and NfL (**d**) between controls or presymptomatic carriers and participants in symptomatic groups with a CDR® + NACC-FTLD global score of 0 or 0.5. **a-d** Heat maps show AUCs comparing controls to the indicated groups that either include all individuals (All participants) or only those with an CDR® + NACC-FTLD global score of 0 or 0.5 (Mildly impaired participants) from unadjusted or age and sex-adjusted analyses. The number of participants (n) is shown. p values are from analysis adjusted for age and sex; *p* < 0.0071 (comparisons to controls) or *p* < 0.0083 (comparisons to presymptomatics) were considered statistically significant. ****p < 0.0001 and **p < 0.01 (comparison to controls); ^####^*p* < 0.0001, ^###^*p* < 0.001 and ^##^*p* < 0.01 (comparison to presymptomatic mutation carriers). Horizontal bars represent median GFAP or NfL concentrations, which are shown on the base 2 logarithm scale. See Tables S8 and S9 relating to panels a and b, and Tables S14 and S15 relating to panels c and d

We compared baseline plasma GFAP concentrations between controls or presymptomatic mutation carriers with all other groups (Table S8 and S9). GFAP was elevated in all groups except presymptomatic mutation carriers and participants with MBCI when compared to controls (all *p* ≤ 0.001), and in participants with bvFTD (*p* = 1.9E-06), nfvPPA (*p* = 0.006) or CBS (*p* = 5.1E-05) when compared to presymptomatic mutation carriers (Fig. 2a, Table S9). In contrast, NfL was significantly higher in all groups, including presymptomatic mutation carriers and participants with MBCI, when compared to controls (all *p* ≤ 0.004), and in all groups when compared to presymptomatic mutation carriers (*p* ≤ 0.002) (Fig. 2b, Table S9). Results were largely similar when stratifying by symptom duration (≤ 5 years vs. > 5 years; Tables S10 and S11).

When performing baseline GFAP pair-wise comparisons between FTD syndromes, GFAP did not differ among them (Table S12). However, it was lower in individuals with MBCI compared to those with bvFTD (*p* = 7.7E-05) or CBS (*p* = 6.8E-04). Similarly, NfL did not differ among FTD syndromes, but it was lower in the MBCI group compared to all other groups (all *p* < 0.001) (Table S12).

### GFAP poorly discriminates controls from participants with mild behavioral and/or cognitive impairment

The data above suggest that, compared to GFAP, plasma NfL better discriminates controls from individuals with MBCI or an FTD syndrome, and this was further corroborated by estimating AUC values (Figs. 2a and 2b, Tables S8 and S9). Baseline GFAP distinguished controls from symptomatic groups with an age and sex adjusted AUC value of 0.53 for participants with MBCI, and AUCs ranging from 0.56 to 0.68 for all other symptomatic groups, the latter indicating poor to moderate discriminatory ability (Fig. 2a, Table S9). Conversely, baseline NfL discriminated controls from symptomatic groups with an age and sex adjusted AUC value of 0.69 for participants with MBCI, and AUCs ranging from 0.78 to 0.87 for all other symptomatic groups, the latter indicating good to excellent discriminatory ability (Fig. 2b, Table S9). Similar findings for baseline GFAP or NfL were observed when distinguishing presymptomatic mutation carriers from symptomatic groups (Tables S8 and S9) and when stratifying by symptom duration (≤ 5 years vs. > 5 years; Tables S10 and S11). Including both GFAP and NfL when comparing controls or presymptomatic mutation carriers with each phenotype group did not markedly increase AUC values when compared to NfL alone (Table S13).

Further underscoring differences between baseline biomarkers in discriminating asymptomatic individuals from symptomatic individuals, we observed that, compared to controls, NfL (p ≤ 0.004) but not GFAP (p = 0.013 to 0.74), was significantly higher in participants with a CDR® + NACC-FTLD global score of 0 or 0.5 after correcting for multiple comparisons (Figs. 2c and 2d, Tables S14 and S15). Indeed, when considering only these participants with questionable or minimal impairment, the accuracy of baseline GFAP in discriminating controls from each symptomatic group was poor; age and sex adjusted AUC values ranged from 0.52 to 0.61 (Fig. 2c, Table S15). In contrast, baseline NfL distinguished controls from symptomatic groups with an age and sex adjusted AUC value of 0.69 for participants with MBCI, and AUC values ranging from 0.85 to 0.93 for all other symptomatic groups (Fig. 2d, Table S15). Findings were similar when comparing presymptomatic mutation carriers to symptomatic groups (Tables S14 and S15).

### GFAP demonstrates less utility as a susceptibility/risk biomarker compared to NfL

On the basis of our findings above that baseline NfL concentrations may facilitate the discrimination of asymptomatic individuals from those with early stage disease, and reports that blood NfL may facilitate the identification of presymptomatic mutation carriers at risk of phenoconversion [5, 39–41], we examined whether baseline plasma GFAP can similarly distinguish the 74 presymptomatic mutation carriers who did not develop symptoms within one year from baseline from the 29 presymptomatic mutation carriers who did phenoconvert. The 24 presymptomatic individuals for whom conversion status could not be determined due to limited follow-up data were excluded from these analyses (Table S4). For phenoconverters, the median time from baseline to conversion was 2.2 years (Range: 1.0–7.7 years), whereas the median time from baseline to last follow-up in non-converters was 3.9 years (Range: 1.0–8.1 years).

**Fig. 3:**
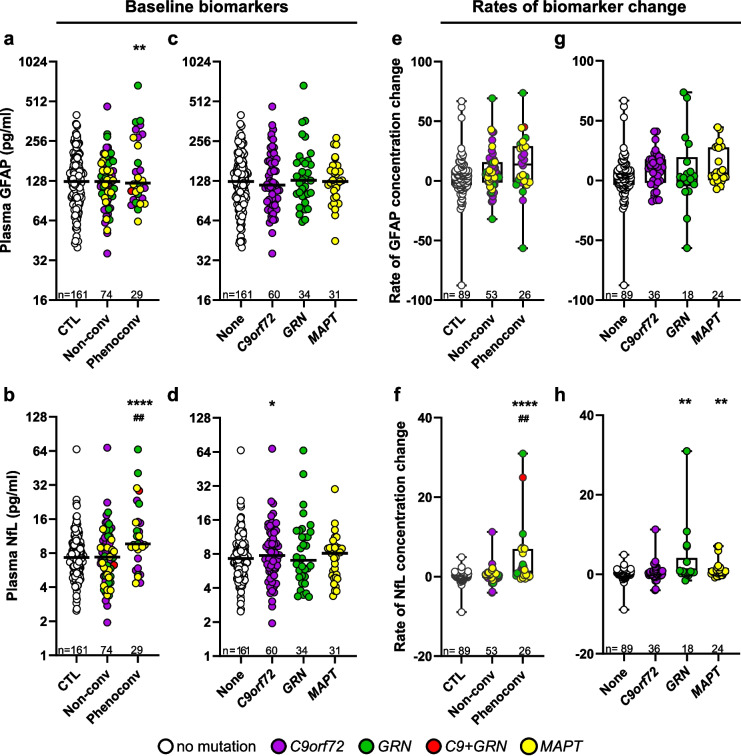
GFAP demonstrates less utility as a susceptibility/risk biomarker compared to NfL. **a, b** Comparison of baseline GFAP (**a**) or NfL (**b**) concentrations in presymptomatic carriers who phenoconverted to controls or to presymptomatic carriers who remained asymptomatic for at least one year. **c, d** Comparison of baseline GFAP (**c**) or NfL (**d**) concentrations in controls to presymptomatic individuals with either a *C9orf72*, *GRN* or *MAPT* mutation. **e, f** Comparison of GFAP (**e**) or NfL (**f**) rates of change in presymptomatic carriers who phenoconverted to controls or to presymptomatic carriers who remained asymptomatic for at least one year. **g**, **h** Comparison of GFAP (**g**) or NfL (**h**) rates of change in controls to presymptomatic individuals with either a *C9orf72*, *GRN* or *MAPT* mutation. The number of participants (n) is shown. *p* values are from analysis adjusted for age and sex. *p* < 0.0167 is considered statistically significant. ****p < 0.0001, **p < 0.01 and *p = 0.013 (comparison to controls); ^##^*p* < 0.01 (comparison of phenoconverters to non-converters). In panels **a-d**, horizontal bars represent median GFAP or NfL concentrations, which are shown on the base 2 logarithm scale. In panels **e–h**, rates of GFAP or NfL change are shown with box and whiskers plots representing minimum, first quartile, median, third quartile, and maximum values. See Table S16 relating to panels **a**, **b**, **e** and **f**, and Table S18 relating to panels **c**, **d**, **g** and **h**

Compared to controls, both baseline GFAP (*p* = 0.006) and NfL (*p* = 3.4E-07) were significantly higher in presymptomatic phenoconverters, but not in non-converters (Fig. 3a and b, Table S16). However, when comparing baseline biomarkers between presymptomatic mutation carriers who did or did not phenoconvert, NfL (*p* = 0.005) but not GFAP (*p* = 0.19) was significantly elevated in phenoconverters compared to non-converters. In assessing the ability of each biomarker to distinguish presymptomatic phenoconverters from non-converters or controls (Table S16), we found that GFAP differentiated controls from converters with an age and sex adjusted AUC value of 0.61, and between non-converters and converters with an adjusted AUC of 0.54. In comparison, NfL differentiated controls from converters with an age and sex adjusted AUC of 0.77, and between non-converters and converters with an adjusted AUC of 0.68. Including both GFAP and NfL in the model comparing controls or non-converters to converters did not increase AUC values when compared to NfL alone (Table S17).

Although the limited number of phenoconverters precluded their analyses by mutation status, we compared baseline GFAP or NfL in controls to presymptomatic mutation carriers with either a *C9orf72*, *GRN* or *MAPT* mutation. When compared to the control group, GFAP did not differ between any mutation group, while NfL was higher in the *C9orf72* group (*p* = 0.013) but not the *GRN* or *MAPT* groups (Fig. 3c and d, Table S18).

Next, assuming a more rapid increase in GFAP or NfL over time may foretell the imminence of phenoconversion, we compared rates of biomarker concentration change among controls, presymptomatic phenoconverters and non-converters for individuals with longitudinal biomarker measurements at least one year from baseline (Fig. 3e and f, Table S16). Compared to controls, the rate of change of NfL (*p* = 2.6E-07), but not GFAP (*p* = 0.017), was significantly higher in presymptomatic phenoconverters but not non-converters. When comparing rates of biomarker change between presymptomatic mutation carriers who did or did not phenoconvert, NfL (*p* = 0.002) but not GFAP (*p* = 0.27) rate of change was significantly elevated in phenoconverters compared to non-converters after correcting for multiple comparisons. The rate of GFAP change distinguished controls from phenoconverters with an adjusted AUC of 0.65, and between non-converters and converters with an adjusted AUC of 0.58. In comparison, the rate of NfL change distinguished controls from converters with an adjusted AUC of 0.78, and between non-converters and converters with an adjusted AUC of 0.70. Combined, these data indicate that NfL better discriminates phenoconverters from controls and presymptomatic non-converters.

When examining rates of GFAP or NfL change between controls and presymptomatic mutation carriers with a *C9orf72*, *GRN* or *MAPT* mutation, no difference in rates of GFAP change were noted. However, compared to controls, NfL rates of change were higher in *GRN* (*p* = 0.006) and *MAPT* (*p* = 0.006) mutation carriers but not *C9orf72* mutation carriers (*p* = 0.05) (Fig. 3g and h, Table S18).

### GFAP and NfL trajectories across prodromal and symptomatic phases

**Fig. 4:**
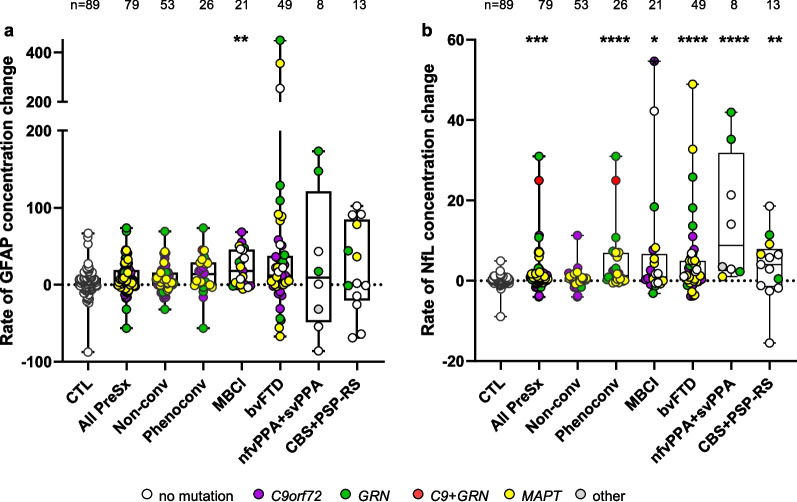
Rates of GFAP and NfL change across prodromal and symptomatic phases. **a**,**b** For individuals with one or more serial GFAP (**a**) or NfL (**b**) measurements at least one year from baseline, we show comparisons of rate of change in biomarker concentrations per year for controls, all presymptomatic mutation carriers combined (All PreSx), presymptomatic carriers who did not convert (Non-conv), those that did phenoconvert (Phenoconv) and participants with MBCI, bvFTD, PPA or parkinsonian disorders. The number of participants (n) is shown. p values are from analysis adjusted for age and sex when comparing rates of GFAP or NfL change between controls and the indicated groups. *p* < 0.0071 is considered statistically significant; ****p < 0.0001, *** *p* < 0.001 and **p < 0.01. See also Table S19

To further probe and compare changes in GFAP and NfL across disease stages, we evaluated rates of GFAP or NfL change among phenotype groups compared to controls (Fig. 4, Table S19). The rate of GFAP change was greater for participants with MBCI (*p* = 0.002) but not for other phenotype groups when compared to controls. However, the rate of NfL change was greater in the presymptomatic group (*p* = 1.9E-04), the aforementioned phenoconverters (*p* = 2.6E-07), and in participants with MBCI (*p* = 0.003), bvFTD (*p* = 1.1E-06), a PPA (*p* = 5.8E-08) or a Parkinsonian disorder (*p* = 0.003) when compared to controls (Fig. 4, Table S19).

### Plasma GFAP and NfL associate with disease severity indicators

We compared the prognostic potential of plasma GFAP and NfL. For each FTD spectrum disorder separately and for all FTD groups combined, we analyzed associations of baseline biomarkers with baseline indicators of clinical severity (CDR® + NACC-FTLD sum of boxes, CDR® + NACC-FTLDsb), global cognitive function (Montreal Cognitive Assessment, MoCA), language deficits (Northwestern Anagram Test, NAT; Multilingual Naming Test, MINT; category fluency; phonemic fluency) and executive function (Digit Span Backward; Trail Making Test Part B, Trails B). Disease severity indicator characteristics and scores according to phenotype groups are shown in Table S20, and data from unadjusted and adjusted analyses are provided for baseline GFAP (Tables S21 and S22) and baseline NfL (Tables S23 and S24).

For all FTD groups combined and for bvFTD alone, higher GFAP associated with worse performance on all tests except for the MINT and Trails B (Table S22). When assessing each FTD syndrome separately, significant (*p* < 0.0062) or nominally significant (*p* < 0.05) associations with GFAP were seen for nfvPPA (phonemic fluency), svPPA (Digit span backward and Trails B), CBS (CDR® + NACC-FTLDsb, MoCA and Digit Span Backward), PSP-RS (CDR® + NACC-FTLDsb, MoCA and MINT), and MBCI (category fluency).

In adjusted analysis, increased NfL associated with worse performance on all tests for all FTD groups combined and for bvFTD alone (Tables S24). Significant or nominally significant associations with NfL were seen for svPPA (CDR® + NACC-FTLDsb, MoCA, NAT, MINT, category fluency and Digit Span Backward), CBS (CDR® + NACC-FTLDsb, phonemic fluency, and Digit Span Backward), PSP-RS (MoCA, category fluency, phonemic fluency, Digit Span Backward and Trails B), and MBCI (MoCA, and phonemic fluency). For associations of GFAP or NfL with disease severity indicators in the smaller phenotype groups, where power to detect associations was lower, estimated β coefficients were often similar to those observed for the larger bvFTD group; nevertheless, GFAP showed inferior prognostic utility compared to NfL.

For all FTD syndromes combined, we also evaluated whether baseline biomarkers were associated with rates of change in disease severity indicators for individuals with longitudinal clinical data spanning at least one year from baseline. Given the small sample size, this allowed the study of six tests (CDR® + NACC-FTLDsb, MoCA, MINT, category fluency, phonemic fluency and Digit Span Backward) (Table S25). Baseline GFAP failed to associate with rates of change for any of the disease severity indicators. In contrast, increased baseline NfL associated with a faster longitudinal decline in performance for the CDR® + NACC-FTLDsb, MoCA and MINT (all *p* ≤ 0.005).

### Baseline plasma GFAP and NfL predict survival after symptom onset

Given our findings that baseline GFAP and NfL associate with markers of disease severity, we postulated that higher concentrations of these biomarkers would associate with shorter survival in participants with FTD. We tested this hypothesis for the 678 individuals having post-baseline information. The median length of follow-up after symptom onset was five years (range: 1 to 35 years), and 105 participants (15.5%) died.

**Fig. 5:**
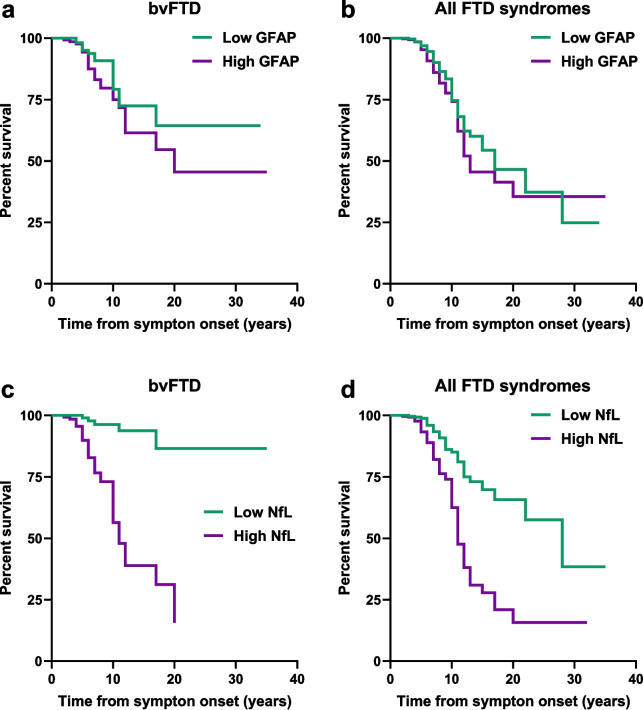
Plasma NfL better predicts survival after symptom onset than plasma GFAP. Comparisons of baseline GFAP (**a, b**) or NfL (**c, d**) concentrations in predicting survival after symptom onset in participants with bvFTD or in the combined group of participants with a FTD syndrome. For ease of presentation, GFAP and NfL were divided into a 2-level categorical variable based on sample medians. bvFTD, *n* = 301 (participants who died, n = 42); all FTD syndromes, *n* = 678 (participants who died, *n* = 105). In (**a**): Low ≤ 247.00 pg/ml, High > 247.00 pg/ml. In (**b**): Low ≤ 237.00 pg/ml, High > 237.00 pg/ml. In (**c**): Low ≤ 42.00 pg/ml, High > 42.00 pg/ml. In (**d**) Low ≤ 32.00 pg/ml, High > 32.00 pg/ml. See also Table S26

Higher GFAP concentrations associated with an increased risk of death after symptom onset in participants with bvFTD (HR = 2.01, *p* = 0.001) and the combined group of FTD syndromes (HR = 1.36, *p* = 0.006). Higher NfL concentrations more strongly associated with a greater risk of death after symptom onset in participants with bvFTD (HR = 2.97, *p* = 4.7E-10), and in all FTD syndromes combined (HR = 2.39, 4.2E-13) (Fig. 5 and Table S26).

We also examined the ability of either biomarker to independently predict survival from symptom onset by estimating the c-index of multivariable Cox regression models that either included or excluded GFAP or NfL (Table S26). Our analyses demonstrated that, except for the bvFTD group, c-indexes only marginally increased when adding GFAP to the multivariable models; for example, the c-index in all FTD syndromes combined increased only by 0.008 (from 0.888 to 0.896 when including GFAP). In contrast, adding NfL to multivariable models yielded greater increases in the c-index; in all FTD syndromes combined, including NfL increased the c-index by 0.03 (from 0.888 to 0.917).

### Associations of GFAP, NfL and the GFAP/NfL ratio with underlying FTLD pathology

**Fig. 6:**
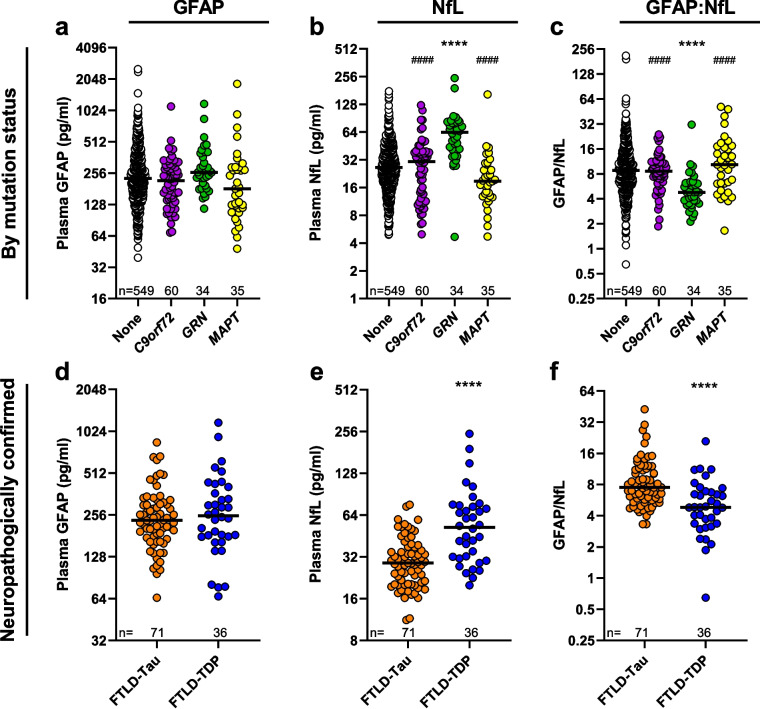
Baseline NfL and GFAP/NfL ratio can discern FTLD-tau from FTLD-TDP pathology. **a-c** Comparisons of baseline GFAP (**a**), NfL (**b**), or GFAP/NfL (**c**) in the combined group of participants with a FTD syndrome with no mutation, or either a *C9orf72, GRN,* or *MAPT* mutation. *p* values are from analysis adjusted for age, sex and symptom duration. *p* < 0.0083 is considered statistically significant; ****p < 0.0001 (comparison to no mutation participants, “None”); ^####^*p* < 0.0001 (comparison to *GRN* mutation carriers). **d-f** Comparisons of GFAP (**d**), NfL (**e**), or GFAP/NfL (**f**) between neuropathologically confirmed participants with FTLD-tau or with FTLD-TDP. *p* values are from analysis adjusted for age at biomarker measurement, sex, symptom duration, and age at death. *p* < 0.025 is considered statistically significant; ****p < 0.0001. In all panels, the number of participants (n) is shown, and horizontal bars represent median GFAP, NfL or GFAP/NfL concentrations, which are shown on the base 2 logarithm scale. See Tables S27 and S28 for panels (**a-c**), and Table S29 for panels (**d-f**)

Given reported evidence that the ratio of GFAP/NfL concentrations discriminates tau vs. TDP-43 pathology [42], we sought to validate these findings. Towards this end, we examined baseline GFAP, NfL and GFAP/NfL in individuals clinically diagnosed with an FTD syndrome and who carry a pathogenic variant associated with FTLD-Tau (*MAPT*) or FTLD-TDP (*C9orf72* or *GRN*) (Fig. 6a-c, Tables S27 and S28). GFAP concentrations in participants with a *C9orf72*, *GRN* or *MAPT* mutation did not differ from FTD participants with no mutation, nor did GFAP differ among the three mutation groups when performing pair-wise comparisons (Fig. 6a, Tables S27 and S28). Baseline NfL was significantly higher in FTD participants with a *GRN* mutation compared to participants with no mutation (*p* = 1.6E-12), and higher in *GRN* mutation carriers when compared to *C9orf72* (p = 2.7E-06) or *MAPT* (*p* = 9.5E-08) mutation carriers (Fig. 6b, Tables S27 and S28). Conversely, the GFAP/NfL ratio was significantly lower in *GRN* mutation carriers compared to mutation-free counterparts (*p* = 4.8E-06), and when compared to *C9orf72* (*p* = 1.4E-05) or *MAPT* (*p* = 3.7E-05) mutation carriers (Fig. 6c, Tables S27 and S28).

To specifically determine the performance of baseline GFAP, NfL or the GFAP/NfL ratio in distinguishing tau versus TDP-43 pathology, we evaluated whether these markers differ between individuals with neuropathologically confirmed FTLD-tau or FTLD-TDP. GFAP did not differ between FTLD-tau or FTLD-TDP cases (*p* = 0.47), whereas NfL was significantly higher in FTLD-TDP than in FTLD-tau (*p* = 1.8E-07), and GFAP/NfL was significantly lower in FTLD-TDP than FTLD-tau (*p* = 7.6E-05) (Fig. 6d-f, Table S29). NfL and GFAP/NfL respectively discriminated these two groups with an adjusted AUC value of 0.78 and 0.73 while GFAP showed virtually no discriminatory power (AUC = 0.55) (Table S29). While the data suggest that NfL and the GFAP/NfL ratio can discriminate between these two groups, the findings appear to be largely driven by NfL.

### Sensitivity analyses

When accounting for possible correlation in measures between members of the same family, results from our primary analysis were very similar to those using a GEE or marginal model approach (comprehensive data not shown). For example, compared to controls, baseline plasma GFAP concentrations where higher in all groups except presymptomatic mutation carriers and MBCI (all *p* ≤ 6.0E-07), and baseline NfL was significantly higher in all phenotype groups (including presymptomatic mutation carriers and MBCI participants) compared to controls (all *p* ≤ 0.001). As another example, in line with our primary analysis, higher GFAP concentrations were associated with an increased risk of death after symptom onset in participants with bvFTD and the combined group of all FTD syndromes (HR = 2.11, *p* = 6.0E-08 and HR = 1.40, *p* = 1.2E-06, respectively). Likewise, higher NfL associated with an increased risk of death in participants with bvFTD and the combined group of all FTD syndromes (HR = 3.28, *p* = 2.0E-15 and HR = 2.63, *p* = 1.1E-11, respectively).

In a second sensitivity analysis, we compared results from the primary multivariable analysis (where BMI was not adjusted for) to results when also adjusting multivariable models for BMI. For these analyses, we included only participants with BMI data to allow for a fair comparison of findings with and without BMI in the given model. Additional model adjustment for BMI had minimal impact on any findings (data not shown).

## Discussion

Findings from several studies indicate that blood GFAP is elevated in FTD and/or has potential as a prognostic biomarker for FTD [8–14, 17, 18, 23]. Nevertheless, few studies compared the performance of GFAP to NfL, which is the most investigated fluid biomarker in FTD, and one that shows promise as a prognostic biomarker and potentially as a susceptibility/risk marker. By leveraging large cohorts of participants with sporadic or genetic bvFTD, nfvPPA, svPPA, CBS or PSP-RS, and clinically normal individuals with or without FTD-causing mutations, we conducted a head-to-head comparison of plasma GFAP and NfL to elucidate their respective biomarker capabilities. We found that both plasma GFAP and NfL increase in presymptomatic mutation carriers prior to phenoconversion, are significantly elevated in each FTD spectrum disorder, and are associated with clinical markers of disease severity. However, despite our observation that plasma GFAP and NfL concentrations correlate in each phenotype group, which has also been shown by others [8, 9, 16, 21, 23], our findings demonstrate that NfL considerably outperforms GFAP.

Consistent with prior studies [8–16, 43–47], we show that both baseline plasma GFAP and NfL are elevated in each FTD spectrum disorder compared to controls. However, plasma NfL surpassed GFAP in distinguishing controls from individuals with FTD. Combining both plasma GFAP and NfL in the same model did not appreciably increase the discriminatory power owing to the already excellent ability of NfL to distinguish healthy controls from participants, which is in line with a prior report [8]. We also show that neither biomarker differs between nor discriminates among FTD syndromes. Although few studies compared blood GFAP among syndromes, those that did perform group comparisons either showed no difference in blood GFAP between bvFTD, nfvPPA or svPPA [8], or noted higher serum GFAP in nfvPPA compared to CBS and PSP-RS [11]. Similarly, NfL was comparable between nfvPPA and svPPA [48, 49], and between bvFTD, nfvPPA, and svPPA [8, 43]. Finally, in evaluating rates of change of biomarker concentrations, we found that GFAP rates of change in participants with bvFTD, a PPA or a Parkinsonian disorder do not differ from that of controls, whereas NfL rates of change are higher in participants with bvFTD, a PPA or a Parkinsonian disorder. In aggregate, our data suggest that baseline NfL concentrations and their rates of change could potentially serve as a response marker in interventional trials with the expectation that lowering NfL levels would indicate slowing of disease progression.

Given that plasma GFAP and NfL are elevated not only in FTD but also in other neurological diseases such as Alzheimer disease [7], stroke [50, 51], and TBI [6], these biomarkers are not anticipated to serve as FTD-specific diagnostic markers. However, measuring GFAP and NfL could potentially provide a means to respectively confirm or rule out astrogliosis or neuronal injury for individuals in the prodromal stage of FTD. Upon investigating this, we noted that plasma NfL, but not plasma GFAP, is elevated in participants with MBCI and in FTD participants presenting with only mild symptoms. Indeed, NfL showed an excellent ability in discriminating these participants from healthy controls, suggesting that NfL, in tandem with traditional diagnostic testing, may inform the diagnosis of questionable cases and facilitate a more rapid diagnosis.

We and others have shown that blood NfL could prove useful in detecting impending symptom onset in presymptomatic mutation carriers [5, 39–41, 52]. In agreement with our prior studies [5, 40], which included a subset of individuals in the present study, we found that both baseline NfL and rates of NfL change were significantly higher in presymptomatic phenoconverters compared to non-converters, and showed a similar ability to discriminate between these two groups. In contrast, neither baseline GFAP nor its rate of change significantly differed between presymptomatic phenoconverters and non-converters, and the addition of GFAP to models comparing baseline NfL between non-converters and converters did not increase discriminatory power. While GFAP appears an unlikely susceptibility/risk biomarker, we must nonetheless consider that grouping *C9orf72*, *GRN* and *MAPT* mutation carriers may mask its potential utility as a susceptibility/risk biomarker within a given mutation group.

To elucidate the prognostic power of both biomarkers, we examined associations of baseline plasma GFAP or NfL with indicators of global cognitive function, executive function and language ability in each FTD syndrome. In the largest group of bvFTD participants, higher GFAP significantly associated with worse performance on all baseline assessments except for MINT and Trails B. Conversely, GFAP failed to associate with longitudinal changes in any of the assessments. In contrast, higher NfL in participants with bvFTD associated with worse performance at baseline on all assessments, and with a faster longitudinal decline in performance on the CDR® + NACC-FTLDsb, the MoCA, and MINT; overall, NfL showed greater prognostic potential. For the smaller phenotype groups of nfvPPA, svPPA, CBS and PSP-RS, GFAP and NfL associated with some assessment scores but not all; however, this is to be anticipated since each syndrome is initially characterized by a predominant clinical trait and because cohort sizes were smaller. Comparing our observations on the prognostic potential of GFAP or NfL with prior findings is complicated by differences in the FTD syndromes examined, the choice of psychometric tests, and cohort size. Even so, in participants with FTD, GFAP associated with poorer scores on the CDR® + NACC-FTLDsb, phonemic and semantic fluency tests, Trails B, and the Mini-Mental State Exam (MMSE; an alternate cognitive test to the MoCA) [8, 11], which aligns with our results, although other studies showed no such associations [9, 21–23]. Similarly, NfL was found to associate with worse scores on the CDR® + NACC-FTLDsb, MMSE and semantic tests [9, 46, 47], while another study found no association of blood NfL with measures of executive function [53].

To further compare the prognostic performance of GFAP and NfL, in adjusted analysis, we evaluated the ability of baseline GFAP and NfL to predict survival after disease onset in participants with bvFTD or all FTD syndromes combined, finding that both higher GFAP and NfL concentrations associated with an increased risk of death in these groups. Consistent with our findings, some studies found higher blood GFAP [12] and NfL [54, 55] to predict shorter survival in participants with FTD, whereas another study found no association of GFAP with risk of death [11].

In addition to biomarkers that predict phenoconversion and inform prognosis, biomarkers that can classify participants with tau or TDP-43 pathology may also increase the likelihood of successfully developing effective treatments for FTD. For example, biomarkers capable of identifying individuals with tau or TDP-43 pathology in life would allow the enrichment of appropriate participants in clinical trials testing potential treatments targeting tau or TDP-43. This would be especially useful for participants with sporadic bvFTD, who are almost just as likely to have TDP-43 or tau pathology. Recently, the Irwin group reported excellent discrimination of FTLD-tau from FTLD-TDP using the ratio of GFAP and NfL in plasma, which was greater than the discriminatory power of either plasma GFAP or NfL alone [42]. In a similar fashion, we evaluated the performance of baseline GFAP, NfL or the GFAP/NfL ratio in distinguishing tau versus TDP-43 pathology for participants clinically diagnosed with bvFTD, nfvPPA, svPPA, CBS or PSP-RS, and having a neuropathological diagnosis of FTLD-tau or FTLD-TDP. Although GFAP showed virtually no discriminatory power, NfL and GFAP/NfL did discriminate between FTLD-tau and FTLD-TDP groups, but the latter was largely driven by NfL. Discrepancies between our findings and those from the Irwin group, may result from differences in inclusion criteria; whereas participants with ALS or FTLD-TDP type B were included in the Irwin study [42], they were excluded from our study given that TDP-43 type B is associated with FTD-ALS phenotypes [35], and participants with ALS have significantly higher plasma NfL than participants with FTD, which may confound our analyses [5]. While GFAP/NfL may show utility in informing underlying pathologies in participants with FTD, additional antemortem methods to identify tau or TDP-43 pathology are needed. It thus warrants noting that exciting new avenues in the pursuit of tau or TDP-43-related biomarkers are underway, including measures of cryptic peptides as surrogate markers of TDP-43 pathology [56–58], and emerging methods to measure tau and TDP-43 in plasma extracellular vesicles [59], or to detect tau or TDP-43 aggregates in cerebrospinal fluid [60, 61].

Along with the above-mentioned biomarker utilities of NfL, it could also serve as a response biomarker. For example, in a phase 2 clinical trial (NCT04993768) evaluating TPN-101 (a retrotransposon) as a treatment for PSP-RS, it was found to reduce NfL concentrations and to stabilize clinical symptoms [62]. Moreover, clinical trials led by Biogen (NCT04288856) or Wave Life Sciences (NCT04931862), each testing distinct investigational antisense oligonucleotides that target GGGGCC repeat transcripts as a treatment for *C9orf72*-associated ALS/FTD, failed to show clinical benefit in line with increased NfL concentrations [63, 64]. Due to its prognostic utility, NfL measures may additionally improve clinical trial design by enabling the well-balanced stratification of slow and fast progressors across treatment arms, thereby decreasing treatment outcome variability [65, 66].

Strengths of our study include measuring, in a blinded manner and at one site, both plasma GFAP and NfL in a large series of well-characterized individuals diagnosed with bvFTD, nfvPPA, svPPA, PSP and CBS along with presymptomatic mutation carriers and controls; performing head-to-head comparisons of GFAP and NfL; including cross-sectional and longitudinal assessments of both biomarkers; examining the prognostic potential of each biomarker in each syndrome separately; and evaluating the combined use of GFAP and NfL to inform pathology in participants with FTD. Also of importance, the GFAP and NfL data, along with extensive clinical data, are accessible to the scientific community through ALLFTD.

Our study also has limitations. Participants enrolled in the study may not represent the general FTD population, and diagnoses were made using clinical rather than neuropathologic assessments. Also, despite a number of studies demonstrating associations of lower BMI with increased blood NfL concentrations in healthy controls, individuals with mild cognitive impairment, or patients with neurological diseases [67–72], we did not adjust our primary multivariable models for BMI as this would have excluded the 18% of participants for whom BMI data were not available. Nonetheless, we performed a secondary analysis adjusting for BMI for individuals with BMI data and results were largely consistent between our primary and secondary analysis. Our analysis also did not account for the varied medications or supplements taken by participants; however, findings from a recent study suggest that microglia play a role in clearing NfL and that certain drugs, like minocycline, inhibit this clearance and consequently may increase NfL concentrations in the absence of neurodegeneration [73]. Accordingly, the use of minocycline and other medications could confound the interpretation of NfL measures. It also bears mentioning that some study participants were family members of other participants both within and between phenotype groups. On one hand, this could be viewed as a strength as comparisons between groups are less likely to be confounded by unmeasured environmental and socioeconomic factors when family members are among the groups being compared. On the other hand, the inclusion of family members within and between groups has the potential to introduce more correlation between measures (e.g., NfL and GFAP) than what would be observed between unrelated individuals. We have thus accounted for this possibility by performing sensitivity analyses and observed similar findings between our primary analyses and analyses in which we used GEE or marginal model approaches. Finally, due to the smaller sample size of some groups, the possibility of a false-negative finding is important to consider. We cannot conclude that a true difference or association does not exist simply due to the occurrence of a non-significant p-value, accordingly, emphasis should be placed on 95% confidence limits when interpreting results.

## Conclusions

The present study provides a roadmap for the rigorous evaluation of novel FTD biomarkers. In aggregate, our thorough cross-sectional and longitudinal analyses from the largest series of participants with an FTD syndrome and asymptomatic individuals with a FTD-causing mutation show that plasma NfL consistently outperforms plasma GFAP as a prognostic, susceptibility/risk, and predictive biomarker. Findings from our study also underscore the need to include leading biomarkers in investigations evaluating new biomarkers if we are to fully ascertain their performance and clinical value – an approach that is adopted too infrequently.

## Supplementary Information

Supplementary Material 1: Table S1: Summary of relatedness when considering each phenotype group separately. Table S2: Summary of relatedness in each phenotype group in comparison to controls. Table S3: Summary of relatedness in each phenotype group in comparison to the presymptomatic group. Table S4: Breakdown of baseline and longitudinal biomarker measures and rates of their change in phenotype groups. Table S5: Correlations between baseline GFAP and baseline NfL. Table S6: Associations of baseline biomarkers with age, sex, symptom duration, and BMI in phenotype groups – unadjusted analysis. Table S7: Associations of baseline biomarkers with age, sex, symptom duration, and BMI in phenotype groups – adjusted analysis. Table S8: Comparisons of baseline biomarkers between controls or presymptomatic mutation carriers and phenotype groups – unadjusted analysis. Table S9: Comparisons of baseline biomarkers between controls or presymptomatic mutation carriers and phenotype groups – adjusted analysis. Table S10: Baseline biomarker comparisons between asymptomatic groups and symptomatic groups stratified by symptom duration – unadjusted analysis. Table S11: Baseline biomarker comparisons between asymptomatic groups and symptomatic groups stratified by symptom duration – adjusted analysis. Table S12: Comparisons of baseline GFAP and NfL concentration between disease groups. Table S13: AUC values from models including both biomarkers when comparing asymptomatic groups to phenotype groups. Table S14: Comparisons of baseline biomarkers between asymptomatic groups and mildly impaired phenotype groups – unadjusted analysis. Table S15: Comparisons of baseline biomarkers between asymptomatic groups and mildly impaired phenotype groups – adjusted analysis. Table S16: Comparison of baseline biomarkers or their rates of change between controls and phenoconverters or non-converters. Table S17: AUC values from models including both biomarkers when comparing controls to phenoconverters or non-converters. Table S18: Comparison of baseline biomarkers or their rates of change by mutation status in asymptomatic groups. Table S19: Comparison of rate of change in GFAP and NfL concentrations between controls and phenotype groups. Table S20: Characteristics of disease severity indicators according to disease group. Table S21: Associations of baseline GFAP with baseline disease indicators in FTD syndromes and MBCI – unadjusted analysis. Table S22: Associations of baseline GFAP with baseline disease indicators in FTD syndromes and MBCI – adjusted analysis. Table S23: Associations of baseline NfL with baseline disease indicators in FTD syndromes and MBCI – unadjusted analysis. Table S24: Associations of baseline NfL with baseline disease indicators in FTD syndromes and MBCI – adjusted analysis. Table S25: Associations of baseline biomarkers with rates of disease indicator change in all FTD syndromes combined. Table S26: Associations of baseline GFAP and NfL with survival after symptom onset. Table S27: Comparisons of baseline biomarkers or their ratio by mutation status in all FTD syndromes combined. Table S28: Comparisons of baseline biomarkers or their ratio among mutation groups in all FTD syndromes combined. Table S29: Differences in baseline biomarkers or their ratio between FTLD-tau or FTLD-TDP pathology in FTD syndromes

## Data Availability

De-identified participant clinical, demographic and biomarker data are available from ALLFTD upon request. Investigators are required to complete the Request Clinical Data form on the request portal (https://www.allftd.org/data) and to review the data sharing and publication policy. Data that could identify a participant are not provided. Data requests are reviewed quarterly and generally fulfilled approximately four weeks after they are approved depending on the complexity of the request. Any additional information required to reanalyze the data reported in this paper is available from the corresponding author and ALLFTD.

## Abbreviations

ALLFTD: ARTFL-LEFFTDS Longitudinal Frontotemporal Lobar Degeneration
ALS: Amyotrophic lateral sclerosis
ARTFL: Advancing Research and Treatment for Frontotemporal Lobar Degeneration
AUC: Area under the receiver operating characteristic curve
β: Beta coefficient
bvFTD: Behavioral variant frontotemporal dementia
BMI: Body mass index
CBS: Corticobasal syndrome
CDR® + NACC-FTLD: CDR® Dementia Staging Instrument plus behavior and language domains from the National Alzheimer’s Disease Coordinating Center Frontotemporal Lobar Degeneration module
CDR® + NACC-FTLDsb: CDR® + NACC-FTLD sum of boxes
CI: Confidence interval
FTD: Frontotemporal dementia
FTD-ALS: bvFTD with ALS
FTLD: Frontotemporal lobar degeneration
FTLD-tau: Frontotemporal lobar degeneration with tau pathology
FTLD-TDP: Frontotemporal lobar degeneration with TDP-43 pathology
GEE: Generalized estimating equations
GFAP: Glial fibrillary acidic protein
HR: Hazard ratio
LEFFTDS: Longitudinal Evaluation of Familial Frontotemporal Dementia Subjects
MBCI: Mild behavioral and/or cognitive impairments
MINT: Multilingual naming test
MMSE: Mini mental state examination
MoCA: Montreal cognitive assessment
NAT: Northwestern anagram test
NCRAD: National Centralized Repository of Alzheimer's Disease and Related Dementias
NfL: Neurofilament light
nfvPPA: Nonfluent/agrammatic primary progressive aphasia
PSP-RS: Progressive supranuclear palsy-Richardson syndrome
svPPA: Semantic variant primary progressive aphasia
TDP-43: TAR-DNA binding protein 43
Trails B: Trail making test part B
TBI: Traumatic brain injury
