# Supplementary material for: Comprehensive cross-sectional and longitudinal comparisons of plasma glial fibrillary acidic protein and neurofilament light across FTD spectrum disorders
Source: Mol Neurodegener. 2025 Mar 12;20:30. doi: 10.1186/s13024-025-00821-4 (PMC11905702; doi:10.1186/s13024-025-00821-4)
Supplement: Supplementary file 1 — Supplementary Material 1: Table S1: Summary of relatedness when considering each phenotype group separately. Table S2: Summary of relatedness in each phenotype group in comparison to controls. Table S3: Summary of relatedness in each phenotype group in comparison to the presymptomatic group. Table S4: Breakdown of baseline and longitudinal biomarker measures and rates of their change in phenotype groups. Table S5: Correlations between baseline GFAP and baseline NfL. Table S6: Associations of baseline biomarkers with age, sex, symptom duration, and BMI in phenotype groups – unadjusted analysis. Table S7: Associations of baseline biomarkers with age, sex, symptom duration, and BMI in phenotype groups – adjusted analysis. Table S8: Comparisons of baseline biomarkers between controls or presymptomatic mutation carriers and phenotype groups – unadjusted analysis. Table S9: Comparisons of baseline biomarkers between controls or presymptomatic mutation carriers and phenotype groups – adjusted analysis. Table S10: Baseline biomarker comparisons between asymptomatic groups and symptomatic groups stratified by symptom duration – unadjusted analysis. Table S11: Baseline biomarker comparisons between asymptomatic groups and symptomatic groups stratified by symptom duration – adjusted analysis. Table S12: Comparisons of baseline GFAP and NfL concentration between disease groups. Table S13: AUC values from models including both biomarkers when comparing asymptomatic groups to phenotype groups. Table S14: Comparisons of baseline biomarkers between asymptomatic groups and mildly impaired phenotype groups – unadjusted analysis. Table S15: Comparisons of baseline biomarkers between asymptomatic groups and mildly impaired phenotype groups – adjusted analysis. Table S16: Comparison of baseline biomarkers or their rates of change between controls and phenoconverters or non-converters. Table S17: AUC values from models including both biomarkers when comparing controls to phenoconverters [file 13024_2025_821_MOESM1_ESM.docx]

**Supplementary Information**

**Comprehensive cross-sectional and longitudinal comparisons of plasma glial fibrillary acidic protein and neurofilament light across FTD spectrum disorders**

**Table S1: Summary of relatedness when considering each phenotype group separately**

| Phenotype group | N | Number of unrelated participants | Number of related participants | Number of different families in the related participants | Family details |
| --- | --- | --- | --- | --- | --- |
| Controls | 161 | 108 | 53 | 21 | 15 families with 2 members, 4 families with 3 members, 1 family with 5 members, 1 family with 6 members |
| Presymptomatic | 127 | 81 | 46 | 21 | 17 families with 2 members, 4 families with 3 members |
| bvFTD | 308 | 296 | 12 | 6 | 6 families with 2 members |
| nfvPPA | 76 | 76 | 0 | 0 | N/A |
| svPPA | 83 | 83 | 0 | 0 | N/A |
| CBS | 92 | 92 | 0 | 0 | N/A |
| PSP-RS | 143 | 143 | 0 | 0 | N/A |
| MBCI | 67 | 59 | 8 | 4 | 4 families with 2 members |

**Table S2: Summary of relatedness in each phenotype group in comparison to controls**

| Phenotype group | N | Number of participants unrelated to controls | Number participants related to controls | Number of different families in participants related to controls |
| --- | --- | --- | --- | --- |
| Presymptomatic | 127 | 49 | 78 | 28 |
| bvFTD | 308 | 268 | 40 | 23 |
| nfvPPA | 76 | 74 | 2 | 1 |
| svPPA | 83 | 83 | 0 | N/A |
| CBS | 92 | 89 | 3 | 2 |
| PSP-RS | 143 | 140 | 3 | 2 |
| MBCI | 67 | 38 | 29 | 16 |
| Shown are the number of participants in the given group that are related to participants in the control group. | | | | |

**Table S3: Summary of relatedness in each phenotype group in comparison to the presymptomatic group**

| Phenotype group | N | Number of participants unrelated to presymptomatics | Number of participants related to presymptomatics | Number of different families in participants related to presymptomatics |
| --- | --- | --- | --- | --- |
| bvFTD | 308 | 272 | 36 | 17 |
| nfvPPA | 76 | 74 | 2 | 1 |
| svPPA | 83 | 82 | 1 | 1 |
| CBS | 92 | 91 | 1 | 1 |
| PSP-RS | 143 | 143 | 0 | N/A |
| MBCI | 67 | 48 | 19 | 13 |
| Shown are the number of participants in the given group that are related to participants in the presymptomatic group. | | | | |

**Table S4: Breakdown of baseline and longitudinal biomarker measures and rates of their change in phenotype groups**

| Phenotype group | Number of participants | Number of plasma samples per participant:  Median (range) | Total number of baseline and longitudinal samples | Number of participants for whom rates of GFAP and NfL change could be calculated^4^ |
| --- | --- | --- | --- | --- |
| Controls | 161 | 2 (1-7) | 373 | 89 |
| All presymptomatic mutation carriers | 127 | 3 (1-7) | 344 | 79 |
| Non-converters^1^ | 74 | 3 (1-6) | 211 | 53 |
| Phenoconverters^2^ | 29 | 3 (1-7) | 100 | 26 |
| Unknown conversion status^3^ | 24 | 1 (1-4) | 33 | 0 |
| bvFTD | 308 | 1 (1-6) | 398 | 49 |
| nfvPPA | 76 | 1 (1-4) | 94 | 7 |
| svPPA | 83 | 1 (1-2) | 84 | 1 |
| CBS | 92 | 1 (1-4) | 119 | 8 |
| PSP-RS | 143 | 1 (1-4) | 160 | 5 |
| MBCI | 67 | 1 (1-7) | 119 | 21 |
| ^1^Non-converters were presymptomatic at baseline and known to have remained asymptomatic for at least one year from baseline. ^2^Phenoconverters were presymptomatic at baseline but subsequently diagnosed as symptomatic at a later visit. ^3^The 24 presymptomatic individuals with an unknown conversion status had insufficient follow-up data to be categorized as a non-converter or a phenoconverter. ^4^Rates of GFAP or NfL change per year were calculated only for individuals with one or more GFAP and NfL measurements at least one year from baseline. | | | | |

**Table S5: Correlations between baseline GFAP and baseline NfL**

| Phenotype group/association | Spearman’s *r* (95% CI) | p value |
| --- | --- | --- |
| Controls (N=161) | 0.59 (0.48, 0.68) | <2.2E-16 |
| Presymptomatic mutation carriers (N=127) | 0.52 (0.38, 0.64) | 3.57E-10 |
| bvFTD (N=308) | 0.51 (0.42, 0.59) | <2.2E-16 |
| nfvPPA (N=76) | 0.46 (0.26, 0.62) | 4.39E-05 |
| svPPA (N=83) | 0.34 (0.13, 0.52) | 0.002 |
| CBS (N=92) | 0.31 (0.11, 0.48) | 0.003 |
| PSP-RS (N=143) | 0.28 (0.12, 0.43) | 6.57E-04 |
| MBCI (N=67) | 0.62 (0.44, 0.75) | 6.51E-08 |
| All participants (N=1057) | 0.61 (0.57, 0.65) | <2.2E-16 |
| Combined group of bvFTD, nfvPPA, svPPA, CBS, and PSP-RS (N=702) | 0.43 (0.37, 0.49) | <2.2E-16 |
| CI=confidence interval. p values result from Spearman’s test of correlation. p values < 0.005 are considered statistically significant after applying a Bonferroni correction for multiple testing. | | |

**Table S6: Associations of baseline biomarkers with age, sex, symptom duration, and BMI in phenotype groups – unadjusted analysis**

|  | Association with GFAP | | Association with NfL | |
| --- | --- | --- | --- | --- |
| Phenotype group/association | β (95% CI) | p value | β (95% CI) | p value |
| Controls (N=161) |  |  |  |  |
| Association with age (per 10-year increase) | 0.42 (0.33, 0.51) | 7.89E-16 | 0.37 (0.29, 0.46) | 9.00E-15 |
| Association with sex (females vs. males) | 0.27 (0.05, 0.49) | 0.017 | 0.32 (0.12, 0.52) | 0.002 |
| Association with BMI (per 5-unit increase) | -0.07 (-0.17, 0.03) | 0.19 | -0.03 (-0.12, 0.07) | 0.59 |
| Presymptomatic mutation carriers (N=127) |  |  |  |  |
| Association with age (per 10-year increase) | 0.38 (0.28, 0.49) | 6.05E-11 | 0.45 (0.32, 0.58) | 6.64E-10 |
| Association with sex (females vs. males) | 0.19 (-0.05, 0.44) | 0.12 | 0.23 (-0.08, 0.53) | 0.14 |
| Association with BMI (per 5-unit increase) | 0.04 (-0.08, 0.16) | 0.51 | -0.07 (-0.22, 0.08) | 0.39 |
| bvFTD (N=308) |  |  |  |  |
| Association with age (per 10-year increase) | 0.35 (0.24, 0.46) | 1.33E-09 | 0.15 (0.01, 0.28) | 0.033 |
| Association with sex (females vs. males) | 0.59 (0.39, 0.79) | 1.66E-08 | 0.65 (0.41, 0.88) | 1.36E-07 |
| Association with symptom duration (per 5-year increase) | 0.07 (-0.03, 0.18) | 0.18 | -0.15 (-0.28, -0.03) | 0.016 |
| Association with BMI (per 5-unit increase) | -0.11 (-0.20, -0.03) | 0.010 | -0.17 (-0.27, -0.08) | 0.0005 |
| nfvPPA (N=76) |  |  |  |  |
| Association with age (per 10-year increase) | 0.30 (0.10, 0.49) | 0.004 | 0.03 (-0.17, 0.23) | 0.75 |
| Association with sex (females vs. males) | 0.48 (0.16, 0.79) | 0.003 | 0.38 (0.08, 0.68) | 0.015 |
| Association with symptom duration (per 5-year increase) | 0.34 (-0.01, 0.68) | 0.056 | -0.27 (-0.60, 0.06) | 0.10 |
| Association with BMI (per 5-unit increase) | -0.15 (-0.38, 0.08) | 0.19 | -0.15 (-0.35, 0.05) | 0.13 |
| svPPA (N=83) |  |  |  |  |
| Association with age (per 10-year increase) | 0.56 (0.32, 0.81) | 1.81E-05 | 0.15 (-0.06, 0.36) | 0.15 |
| Association with sex (females vs. males) | 0.11 (-0.27, 0.49) | 0.57 | 0.15 (-0.14, 0.43) | 0.31 |
| Association with symptom duration (per 5-year increase) | 0.08 (-0.24, 0.40) | 0.62 | 0.04 (-0.21, 0.28) | 0.77 |
| Association with BMI (per 5-unit increase) | -0.14 (-0.39, 0.10) | 0.25 | -0.03 (-0.21, 0.15) | 0.76 |
| CBS (N=92) |  |  |  |  |
| Association with age (per 10-year increase) | 0.22 (0.02, 0.41) | 0.029 | 0.36 (0.14, 0.58) | 0.002 |
| Association with sex (females vs. males) | 0.15 (-0.23, 0.52) | 0.44 | 0.22 (-0.22, 0.65) | 0.32 |
| Association with symptom duration (per 5-year increase) | -0.02 (-0.22, 0.18) | 0.84 | 0.12 (-0.11, 0.35) | 0.31 |
| Association with BMI (per 5-unit increase) | -0.22 (-0.41, -0.03) | 0.024 | -0.17 (-0.40, 0.06) | 0.14 |
| PSP-RS (N=143) |  |  |  |  |
| Association with age (per 10-year increase) | 0.22 (0.07, 0.37) | 0.004 | -0.04 (-0.23, 0.14) | 0.64 |
| Association with sex (females vs. males) | 0.08 (-0.13, 0.29) | 0.47 | 0.26 (0.01, 0.51) | 0.043 |
| Association with symptom duration (per 5-year increase) | 0.13 (-0.05, 0.30) | 0.15 | -0.08 (-0.29, 0.12) | 0.42 |
| Association with BMI (per 5-unit increase) | -0.22 (-0.33, -0.10) | 0.0003 | -0.15 (-0.28, -0.01) | 0.039 |
| MBCI (N=67) |  |  |  |  |
| Association with age (per 10-year increase) | 0.41 (0.26, 0.56) | 9.77E-07 | 0.43 (0.20, 0.66) | 3.75E-04 |
| Association with sex (females vs. males) | 0.04 (-0.39, 0.47) | 0.85 | -0.01 (-0.60, 0.59) | 0.99 |
| Association with symptom duration (per 5-year increase) | 0.08 (-0.07, 0.23) | 0.27 | -0.02 (-0.23, 0.18) | 0.82 |
| Association with BMI (per 5-unit increase) | -0.21 (-0.44, 0.03) | 0.082 | -0.35 (-0.64, -0.07) | 0.017 |
| β=regression coefficient; CI=confidence interval. β values, 95% CIs, and p values result from unadjusted linear regression models. β values are interpreted as the change in mean GFAP or NfL concentration (on the base-2 logarithm scale) corresponding to the increase given in parenthesis (for age, BMI, and symptom duration) or for females in comparison to males (for sex). p values < 0.0167 (controls and presymptomatic mutation carriers) or p values <0.0125 (symptomatic groups) are considered statistically significant after applying a Bonferroni correction for multiple testing. | | | | |

**Table S7: Associations of baseline biomarkers with age, sex, symptom duration, and BMI in phenotype groups – adjusted analysis**

|  | Association with GFAP | | Association with NfL | |
| --- | --- | --- | --- | --- |
| Phenotype group/association | β (95% CI) | p value | β (95% CI) | p value |
| Controls (N=161) |  |  |  |  |
| Association with age (per 10-year increase) | 0.41 (0.32, 0.51) | 1.55E-15 | 0.36 (0.28, 0.45) | 1.35E-14 |
| Association with sex (females vs. males) | 0.20 (0.02, 0.39) | 0.030 | 0.26 (0.09, 0.43) | 0.003 |
| Association with BMI (per 5-unit increase) | -0.09 (-0.17, -0.01) | 0.030 | -0.04 (-0.11, 0.04) | 0.34 |
| Presymptomatic mutation carriers (N=127) |  |  |  |  |
| Association with age (per 10-year increase) | 0.38 (0.27, 0.48) | 5.20E-11 | 0.45 (0.32, 0.58) | 6.13E-10 |
| Association with sex (females vs. males) | 0.18 (-0.02, 0.39) | 0.080 | 0.21 (-0.05, 0.47) | 0.11 |
| Association with BMI (per 5-unit increase) | -0.02 (-0.13, 0.08) | 0.65 | -0.15 (-0.28, -0.01) | 0.035 |
| bvFTD (N=308) |  |  |  |  |
| Association with age (per 10-year increase) | 0.35 (0.25, 0.46) | 2.71E-10 | 0.19 (0.06, 0.32) | 0.004 |
| Association with sex (females vs. males) | 0.60 (0.41, 0.79) | 1.31E-09 | 0.63 (0.40, 0.86) | 1.91E-07 |
| Association with symptom duration (per 5-year increase) | 0.01 (-0.08, 0.11) | 0.78 | -0.18 (-0.30, -0.06) | 0.004 |
| Association with BMI (per 5-unit increase) | 0.25 (-0.04, 0.54) | 0.086 | -0.15 (-0.24, -0.06) | 0.0018 |
| nfvPPA (N=76) |  |  |  |  |
| Association with age (per 10-year increase) | 0.19 (-0.01, 0.38) | 0.066 | -0.00 (-0.20, 0.20) | 0.99 |
| Association with sex (females vs. males) | 0.46 (0.15, 0.78) | 0.005 | 0.35 (0.02, 0.67) | 0.036 |
| Association with symptom duration (per 5-year increase) | 0.36 (0.03, 0.69) | 0.032 | -0.21 (-0.55, 0.13) | 0.22 |
| Association with BMI (per 5-unit increase) | -0.08 (-0.29, 0.14) | 0.47 | -0.13 (-0.33, 0.08) | 0.22 |
| svPPA (N=83) |  |  |  |  |
| Association with age (per 10-year increase) | 0.56 (0.31, 0.82) | 3.00E-05 | 0.14 (-0.07, 0.36) | 0.19 |
| Association with sex (females vs. males) | 0.10 (-0.24, 0.45) | 0.56 | 0.14 (-0.15, 0.43) | 0.35 |
| Association with symptom duration (per 5-year increase) | 0.01 (-0.28, 0.31) | 0.93 | 0.03 (-0.22, 0.27) | 0.83 |
| Association with BMI (per 5-unit increase) | -0.12 (-0.36, 0.13) | 0.35 | 0.05 (-0.16, 0.25) | 0.63 |
| CBS (N=92) |  |  |  |  |
| Association with age (per 10-year increase) | 0.22 (0.02, 0.42) | 0.031 | 0.34 (0.11, 0.56) | 0.004 |
| Association with sex (females vs. males) | 0.10 (-0.28, 0.48) | 0.60 | 0.20 (-0.23, 0.63) | 0.35 |
| Association with symptom duration (per 5-year increase) | -0.05 (-0.25, 0.16) | 0.66 | 0.08 (-0.15, 0.31) | 0.47 |
| Association with BMI (per 5-unit increase) | -0.22 (-0.42, -0.03) | 0.023 | -0.18 (-0.40, 0.04) | 0.10 |
| PSP-RS (N=143) |  |  |  |  |
| Association with age (per 10-year increase) | 0.24 (0.07, 0.40) | 0.005 | -0.01 (-0.20, 0.19) | 0.94 |
| Association with sex (females vs. males) | 0.15 (-0.08, 0.38) | 0.21 | 0.27 (-0.00, 0.54) | 0.053 |
| Association with symptom duration (per 5-year increase) | 0.11 (-0.07, 0.28) | 0.22 | -0.04 (-0.25, 0.16) | 0.68 |
| Association with BMI (per 5-unit increase) | -0.21 (-0.33, -0.08) | 0.0012 | -0.12 (-0.27, 0.02) | 0.099 |
| MBCI (N=67) |  |  |  |  |
| Association with age (per 10-year increase) | 0.42 (0.26, 0.58) | 1.49E-06 | 0.42 (0.18, 0.66) | 8.23E-04 |
| Association with sex (females vs. males) | 0.19 (-0.18, 0.57) | 0.30 | 0.16 (-0.40, 0.73) | 0.57 |
| Association with symptom duration (per 5-year increase) | 0.05 (-0.07, 0.18) | 0.38 | -0.05 (-0.24, 0.14) | 0.59 |
| Association with BMI (per 5-unit increase) | -0.10 (-0.32, 0.11) | 0.34 | -0.23 (-0.54, 0.07) | 0.13 |
| β=regression coefficient; CI=confidence interval. β values, 95% CIs, and p values result from linear regression models that were adjusted for age, sex, and symptom duration (the latter in MBCI and FTD syndromes). β values are interpreted as the change in mean GFAP or NfL concentration (on the base-2 logarithm scale) corresponding to the increase given in parenthesis (for age, BMI, and symptom duration) or for females in comparison to males (for sex). p values < 0.0167 (controls and presymptomatic mutation carriers) or p values <0.0125 are considered statistically significant after applying a Bonferroni correction for multiple testing. | | | | |

**Table S8: Comparisons of baseline biomarkers between controls or presymptomatic mutation carriers and phenotype groups – unadjusted analysis**

|  | Comparisons of GFAP | | | | | Comparisons of NfL | | | | |
| --- | --- | --- | --- | --- | --- | --- | --- | --- | --- | --- |
| Phenotype group | N | Median (min, max) GFAP concentration (pg/ml) | β (95% CI) | p value | AUC (95% CI) | N | Median (min, max) NfL concentration (pg/ml) | β (95% CI) | p value | AUC (95% CI) |
| Controls | 161 | 126.3 (40.0, 402.7) | 0.00 (reference) | N/A | N/A | 161 | 7.3 (2.5, 66.1) | 0.00 (reference) | N/A | N/A |
| Presymptomatic | 127 | 119.9 (36.0, 674.0) | 0.02 (-0.15, 0.18) | 0.85 | 0.51 (0.44, 0.58) | 127 | 7.5 (1.9, 68.2) | 0.10 (-0.08, 0.27) | 0.27 | 0.52 (0.45, 0.59) |
| bvFTD | 308 | 222.8 (33.6, 2544.5) | 0.81 (0.65, 0.98) | 9.84E-21 | 0.75 (0.72, 0.80) | 308 | 25.7 (4.7, 246.4) | 1.75 (1.57, 1.93) | 1.05E-59 | 0.92 (0.89, 0.94) |
| nfvPPA | 76 | 230.9 (88.9, 691.0) | 0.97 (0.77, 1.16) | 1.09E-19 | 0.83 (0.77, 0.88) | 76 | 28.7 (11.7, 96.5) | 2.03 (1.86, 2.21) | 7.90E-61 | 0.99 (0.97, 1.00) |
| svPPA | 83 | 224.4 (67.2, 1492.4) | 0.88 (0.68, 1.08) | 7.21E-16 | 0.79 (0.73, 0.85) | 83 | 29.2 (10.9, 77.7) | 1.95 (1.78, 2.12) | 1.09E-61 | 0.98 (0.97, 1.00) |
| CBS | 92 | 244.5 (39.5, 2388.4) | 1.02 (0.82, 1.22) | 2.69E-20 | 0.83 (0.78, 0.89) | 92 | 28.3 (4.7, 191.8) | 1.91 (1.70, 2.11) | 3.78E-47 | 0.94 (0.90, 0.97) |
| PSP-RS | 143 | 223.9 (81.5, 850.7) | 0.81 (0.66, 0.96) | 1.22E-22 | 0.80 (0.76, 0.85) | 143 | 25.7 (4.9, 112.0) | 1.80 (1.64, 1.96) | 6.75E-67 | 0.96 (0.94, 0.98) |
| MBCI | 67 | 131.9 (28.7, 595.9) | 0.23 (0.01, 0.44) | 0.038 | 0.58 (0.49, 0.66) | 67 | 12.6 (2.5, 146.8) | 0.84 (0.60, 1.08) | 7.93E-11 | 0.73 (0.64, 0.81) |
|  |  |  |  |  |  |  |  |  |  |  |
| Presymptomatic | 127 | 119.9 (36.0, 674.0) | 0.00 (reference) | N/A | N/A | 127 | 7.5 (1.9, 68.2) | 0.00 (reference) | N/A | N/A |
| bvFTD | 308 | 222.8 (33.6, 2544.5) | 0.80 (0.62, 0.98) | 5.88E-17 | 0.76 (0.71, 0.81) | 308 | 25.7 (4.7, 246.4) | 1.65 (1.44, 1.87) | 1.19E-42 | 0.88 (0.85, 0.92) |
| nfvPPA | 76 | 230.9 (88.9, 691.0) | 0.95 (0.75, 1.15) | 2.45E-17 | 0.83 (0.78, 0.89) | 76 | 28.7 (11.7, 96.5) | 1.94 (1.71, 2.17) | 1.00E-39 | 0.96 (0.93, 0.98) |
| svPPA | 83 | 224.4 (67.2, 1492.4) | 0.86 (0.65, 1.07) | 9.70E-14 | 0.79 (0.72, 0.85) | 83 | 29.2 (10.9, 77.7) | 1.85 (1.64, 2.07) | 3.60E-40 | 0.95 (0.92, 0.98) |
| CBS | 92 | 244.5 (39.5, 2388.4) | 1.00 (0.79, 1.22) | 1.88E-17 | 0.83 (0.77, 0.89) | 92 | 28.3 (4.7, 191.8) | 1.81 (1.55, 2.06) | 6.29E-32 | 0.91 (0.87, 0.95) |
| PSP-RS | 143 | 223.9 (81.5, 850.7 | 0.80 (0.64, 0.96) | 1.35E-19 | 0.81 (0.75, 0.86) | 143 | 25.7 (4.9, 112.0) | 1.70 (1.51, 1.90) | 2.46E-45 | 0.93 (0.89, 0.96) |
| MBCI | 67 | 131.9 (28.7, 595.9) | 0.21 (-0.02, 0.44) | 0.069 | 0.58 (0.50, 0.67) | 67 | 12.6 (2.5, 146.8) | 0.74 (0.44, 1.04) | 1.98E-06 | 0.69 (0.61, 0.78) |
| β=regression coefficient; CI=confidence interval; AUC=area under the receiver operating curve. β values, 95% CIs, and p values result from unadjusted linear regression models. β values are interpreted as the difference in the mean GFAP or NfL concentration (on the base-2 logarithm scale) for the given phenotype group in comparison to controls. p values <0.0071 (comparisons vs. controls) and <0.0083 (comparisons with presymptomatic mutation carriers) are considered as statistically significant after applying a Bonferroni correction for multiple testing. | | | | | | | | | | |

**Table S9: Comparisons of baseline biomarkers between controls or presymptomatic mutation carriers and phenotype groups – adjusted analysis**

|  | Comparisons of GFAP | | | | | Comparisons of NfL | | | | |
| --- | --- | --- | --- | --- | --- | --- | --- | --- | --- | --- |
| Phenotype group | N | Median (min, max) GFAP concentration (pg/ml) | β (95% CI) | p value | AUC (95% CI) | N | Median (min, max) NfL concentration (pg/ml) | β (95% CI) | p value | AUC (95% CI) |
| Controls | 161 | 126.3 (40.0, 402.7) | 0.00 (reference) | N/A | N/A | 161 | 7.3 (2.5, 66.1) | 0.00 (reference) | N/A | N/A |
| Presymptomatic | 127 | 119.9 (36.0, 674.0) | 0.13 (-0.00, 0.27) | 0.057 | 0.55 (0.48, 0.62) | 127 | 7.5 (1.9, 68.2) | 0.22 (0.07, 0.37) | 0.004 | 0.60 (0.53, 0.66) |
| bvFTD | 308 | 222.8 (33.6, 2544.5) | 0.60 (0.44, 0.77) | 7.69E-13 | 0.68 (0.63, 0.72) | 308 | 25.7 (4.7, 246.4) | 1.68 (1.49, 1.88) | 1.01E-51 | 0.86 (0.83, 0.89) |
| nfvPPA | 76 | 230.9 (88.9, 691.0) | 0.42 (0.21, 0.63) | 9.43E-05 | 0.60 (0.52, 0.68) | 76 | 28.7 (11.7, 96.5) | 1.65 (1.45, 1.86) | 8.94E-39 | 0.84 (0.78, 0.90) |
| svPPA | 83 | 224.4 (67.2, 1492.4) | 0.35 (0.14, 0.56) | 0.001 | 0.57 (0.50, 0.65) | 83 | 29.2 (10.9, 77.7) | 1.60 (1.41, 1.78) | 2.44E-43 | 0.87 (0.82, 0.93) |
| CBS | 92 | 244.5 (39.5, 2388.4) | 0.60 (0.38, 0.82) | 2.42E-07 | 0.63 (0.55, 0.70) | 92 | 28.3 (4.7, 191.8) | 1.47 (1.24, 1.70) | 2.27E-28 | 0.80 (0.74, 0.86) |
| PSP-RS | 143 | 223.9 (81.5, 850.7 | 0.31 (0.13, 0.49) | 0.001 | 0.56 (0.50, 0.63) | 143 | 25.7 (4.9, 112.0) | 1.49 (1.29, 1.69) | 4.25E-37 | 0.78 (0.73, 0.83) |
| MBCI | 67 | 131.9 (28.7, 595.9) | 0.07 (-0.11, 0.25) | 0.47 | 0.53 (0.45, 0.62) | 67 | 12.6 (2.5, 146.8) | 0.69 (0.47, 0.91) | 2.17E-09 | 0.69 (0.60, 0.78) |
|  |  |  |  |  |  |  |  |  |  |  |
| Presymptomatic | 127 | 119.9 (36.0, 674.0) | 0.00 (reference) | N/A | N/A | 127 | 7.5 (1.9, 68.2) | 0.00 (reference) | N/A | N/A |
| bvFTD | 308 | 222.8 (33.6, 2544.5) | 0.44 (0.26, 0.63) | 1.91E-06 | 0.63 (0.58, 0.68) | 308 | 25.7 (4.7, 246.4) | 1.43 (1.20, 1.65) | 4.52E-30 | 0.80 (0.76, 0.84) |
| nfvPPA | 76 | 230.9 (88.9, 691.0) | 0.34 (0.10, 0.58) | 0.006 | 0.57 (0.49, 0.65) | 76 | 28.7 (11.7, 96.5) | 1.37 (1.08, 1.65) | 1.01E-17 | 0.76 (0.69, 0.83) |
| svPPA | 83 | 224.4 (67.2, 1492.4) | 0.25 (0.01, 0.49) | 0.039 | 0.55 (0.46, 0.63) | 83 | 29.2 (10.9, 77.7) | 1.31 (1.06, 1.57) | 6.13E-20 | 0.79 (0.73, 0.86) |
| CBS | 92 | 244.5 (39.5, 2388.4) | 0.53 (0.28, 0.78) | 5.10E-05 | 0.61 (0.53, 0.68) | 92 | 28.3 (4.7, 191.8) | 1.18 (0.88, 1.47) | 1.58E-13 | 0.72 (0.65, 0.79) |
| PSP-RS | 143 | 223.9 (81.5, 850.7) | 0.24 (0.03, 0.44) | 0.026 | 0.55 (0.48, 0.62) | 143 | 25.7 (4.9, 112.0) | 1.22 (0.96, 1.49) | 1.62E-17 | 0.71 (0.65, 0.77) |
| MBCI | 67 | 131.9 (28.7, 595.9) | -0.06 (-0.25, 0.14) | 0.58 | 0.51 (0.42, 0.60) | 67 | 12.6 (2.5, 146.8) | 0.44 (0.17, 0.72) | 0.002 | 0.61 (0.52, 0.70) |
| β=regression coefficient; CI=confidence interval; AUC=area under the receiver operating curve. β values, 95% CIs, and p values result from linear regression models that were adjusted for age and sex. β values are interpreted as the difference in the mean GFAP or NfL concentration (on the base-2 logarithm scale) for the given phenotype group in comparison to controls. p values <0.0071 (comparisons vs. controls) and <0.0083 (comparisons with presymptomatic mutation carriers) are considered as statistically significant after applying a Bonferroni correction for multiple testing. | | | | | | | | | | |

**Table S10: Baseline biomarker comparisons between asymptomatic groups and symptomatic groups stratified by symptom duration – unadjusted analysis**

|  | Comparisons of GFAP | | | | | Comparisons of NfL | | | | |
| --- | --- | --- | --- | --- | --- | --- | --- | --- | --- | --- |
| Phenotype group/Symptom duration | N | Median (min, max) GFAP concentration (pg/ml) | β (95% CI) | p value | AUC (95% CI) | N | Median (min, max) NfL concentration (pg/ml) | β (95% CI) | p value | AUC (95% CI) |
| Controls | 161 | 126.3 (40.0, 402.7) | 0.00 (reference) | N/A | N/A | 161 | 7.3 (2.5, 66.1) | 0.00 (reference) | N/A | N/A |
| bvFTD |  |  |  |  |  |  |  |  |  |  |
| ≤ 5-years | 201 | 226.0 (48.2, 2544.5) | 0.82 (0.65, 0.99) | 5.75E-19 | 0.76 (0.71, 0.81) | 201 | 28.9 (4.7, 246.4) | 1.87 (1.68, 2.06) | 3.44E-58 | 0.93 (0.90, 0.96) |
| > 5-years | 107 | 213.8 (33.6, 1840.0) | 0.80 (0.60, 0.99) | 4.33E-14 | 0.76 (0.70, 0.82) | 107 | 20.8 (4.7, 163.9) | 1.53 (1.33, 1.73) | 1.01E-36 | 0.90 (0.86, 0.94) |
| nfvPPA |  |  |  |  |  |  |  |  |  |  |
| ≤ 5-years | 57 | 228.1 (88.9, 691.0) | 0.90 (0.69, 1.10) | 4.86E-15 | 0.81 (0.75, 0.88) | 57 | 30.6 (11.7, 96.5) | 2.07 (1.87, 2.27) | 5.70E-52 | 0.98 (0.97, 1.00) |
| > 5-years | 19 | 303.0 (114.5, 665.5) | 1.17 (0.84, 1.51) | 6.10E-11 | 0.86 (0.77, 0.96) | 19 | 28.4 (13.5, 43.9) | 1.93 (1.64, 2.22) | 1.73E-27 | 0.99 (0.97, 1.00) |
| svPPA |  |  |  |  |  |  |  |  |  |  |
| ≤ 5-years | 43 | 207.9 (67.2, 1492.4) | 0.78 (0.53, 1.03) | 4.39E-09 | 0.76 (0.67, 0.84) | 43 | 27.9 (11.6, 60.6) | 1.91 (1.70, 2.12) | 1.26E-43 | 0.98 (0.97, 1.00) |
| > 5-years | 40 | 230.9 (75.6, 988.0) | 0.98 (0.73, 1.23) | 2.90E-13 | 0.82 (0.75, 0.89) | 40 | 31.4 (10.9, 77.7) | 2.00 (1.77, 2.22) | 7.79E-42 | 0.98 (0.97, 1.00) |
| CBS |  |  |  |  |  |  |  |  |  |  |
| ≤ 5-years | 63 | 234.5 (39.5, 2388.4) | 0.97 (0.74, 1.19) | 8.08E-15 | 0.81 (0.74, 0.88) | 63 | 27.3 (4.7, 124.7) | 1.87 (1.65, 2.09) | 3.05E-40 | 0.93 (0.88, 0.98) |
| > 5-years | 29 | 249.5 (111.1, 1084.1) | 1.14 (0.86, 1.41) | 5.56E-14 | 0.88 (0.82, 0.94) | 29 | 28.5 (6.4, 191.8) | 1.99 (1.70, 2.27) | 5.65E-30 | 0.95 (0.90, 1.00) |
| PSP-RS |  |  |  |  |  |  |  |  |  |  |
| ≤ 5-years | 95 | 215.6 (81.5, 637.7) | 0.76 (0.59, 0.93) | 1.41E-16 | 0.79 (0.74, 0.85) | 95 | 26.3 (4.9, 112.0) | 1.86 (1.68, 2.04) | 8.19E-56 | 0.96 (0.93, 0.99) |
| > 5-years | 48 | 232.7 (85.3, 850.7) | 0.92 (0.70, 1.14) | 2.86E-14 | 0.83 (0.77, 0.90) | 48 | 23.9 (8.8, 59.2) | 1.69 (1.48, 1.89) | 1.23E-38 | 0.97 (0.94, 0.99) |
| MBCI |  |  |  |  |  |  |  |  |  |  |
| ≤ 5-years | 55 | 131.9 (28.7, 595.9) | 0.21 (-0.02, 0.44) | 0.074 | 0.57 (0.48, 0.66) | 55 | 13.1 (2.7, 146.8) | 0.88 (0.63, 1.14) | 1.04E-10 | 0.72 (0.63, 0.82) |
| > 5-years | 12 | 155.3 (62.3, 384.0) | 0.31 (-0.10, 0.72) | 0.14 | 0.60 (0.41, 0.80) | 12 | 11.8 (2.5, 53.3) | 0.65 (0.25, 1.04) | 0.002 | 0.74 (0.55, 0.93) |
|  |  |  |  |  |  |  |  |  |  |  |
| Presymptomatic | 127 | 119.9 (36.0, 674.0) | 0.00 (reference) | N/A | N/A | 127 | 7.5 (1.9, 68.2) | 0.00 (reference) | N/A | N/A |
| bvFTD |  |  |  |  |  |  |  |  |  |  |
| ≤ 5-years | 201 | 226.0 (48.2, 2544.5) | 0.81 (0.62, 0.99) | 9.32E-16 | 0.76 (0.71, 0.81) | 201 | 28.9 (4.7, 246.4) | 1.77 (1.55, 1.99) | 2.34E-41 | 0.90 (0.86, 0.93) |
| > 5-years | 107 | 213.8 (33.6, 1840.0) | 0.78 (0.57, 0.99) | 5.66E-12 | 0.76 (0.70, 0.82) | 107 | 20.8 (4.7, 163.9) | 1.43 (1.19, 1.68) | 3.14E-24 | 0.86 (0.81, 0.91) |
| nfvPPA |  |  |  |  |  |  |  |  |  |  |
| ≤ 5-years | 57 | 228.1 (88.9, 691.0) | 0.88 (0.66, 1.10) | 2.09E-13 | 0.82 (0.76, 0.88) | 57 | 30.6 (11.7, 96.5) | 1.97 (1.71, 2.23) | 2.28E-33 | 0.96 (0.93, 0.98) |
| > 5-years | 19 | 303.0 (114.5, 665.5) | 1.16 (0.82, 1.50) | 4.72E-10 | 0.87 (0.78, 0.95) | 19 | 28.4 (13.5, 43.9) | 1.83 (1.43, 2.23) | 9.61E-16 | 0.96 (0.93, 0.99) |
| svPPA |  |  |  |  |  |  |  |  |  |  |
| ≤ 5-years | 43 | 207.9 (67.2, 1492.4) | 0.76 (0.50, 1.03) | 4.69E-08 | 0.76 (0.67, 0.85) | 43 | 27.9 (11.6, 60.6) | 1.81 (1.53, 2.09) | 1.46E-26 | 0.95 (0.92, 0.98) |
| > 5-years | 40 | 230.9 (75.6, 988.0) | 0.96 (0.71, 1.22) | 6.88E-12 | 0.82 (0.75, 0.90) | 40 | 31.4 (10.9, 77.7) | 1.90 (1.60, 2.20) | 7.95E-26 | 0.95 (0.92, 0.98) |
| CBS |  |  |  |  |  |  |  |  |  |  |
| ≤ 5-years | 63 | 234.5 (39.5, 2388.4) | 0.95 (0.71, 1.19) | 7.38E-13 | 0.80 (0.73, 0.88) | 63 | 27.3 (4.7, 124.7) | 1.77 (1.49, 2.05) | 2.78E-26 | 0.90 (0.85, 0.95) |
| > 5-years | 29 | 249.5 (111.1, 1084.1) | 1.12 (0.84, 1.40) | 1.05E-12 | 0.88 (0.82, 0.94) | 29 | 28.5 (6.4, 191.8) | 1.89 (1.52, 2.26) | 1.37E-18 | 0.92 (0.87, 0.97) |
| PSP-RS |  |  |  |  |  |  |  |  |  |  |
| ≤ 5-years | 95 | 215.6 (81.5, 637.7) | 0.74 (0.57, 0.92) | 1.35E-14 | 0.79 (0.74, 0.85) | 95 | 26.3 (4.9, 112.0) | 1.76 (1.54, 1.99) | 5.78E-37 | 0.93 (0.89, 0.97) |
| > 5-years | 48 | 232.7 (85.3, 850.7) | 0.91 (0.67, 1.14) | 8.19E-13 | 0.83 (0.76, 0.89) | 48 | 23.9 (8.8, 59.2) | 1.59 (1.32, 1.86) | 2.82E-23 | 0.93 (0.89, 0.97) |
| MBCI |  |  |  |  |  |  |  |  |  |  |
| ≤ 5-years | 55 | 131.9 (28.7, 595.9) | 0.19 (-0.05, 0.44) | 0.12 | 0.58 (0.49, 0.67) | 55 | 13.1 (2.7, 146.8) | 0.78 (0.47, 1.10) | 2.34E-06 | 0.69 (0.60, 0.78) |
| > 5-years | 12 | 155.3 (62.3, 384.0) | 0.29 (-0.13, 0.72) | 0.17 | 0.60 (0.41, 0.80) | 12 | 11.8 (2.5, 53.3) | 0.55 (0.02, 1.08) | 0.043 | 0.69 (0.51, 0.86) |
| β=regression coefficient; CI=confidence interval; AUC=area under the receiver operating curve. β values, 95% CIs, and p values result from unadjusted linear regression models. β values are interpreted as the difference in the mean GFAP or NfL concentration (on the base-2 logarithm scale) for the given phenotype group in comparison to controls. p values <0.0083 (comparisons vs. controls or vs. presymptomatic mutation carriers) are considered as statistically significant after applying a Bonferroni correction for multiple testing. | | | | | | | | | | |

**Table S11: Baseline biomarker comparisons between asymptomatic groups and symptomatic groups stratified by symptom duration – adjusted analysis**

|  | Comparisons of GFAP | | | | | Comparisons of NfL | | | | |
| --- | --- | --- | --- | --- | --- | --- | --- | --- | --- | --- |
| Phenotype group/Symptom duration | N | Median (min, max) GFAP concentration (pg/ml) | β (95% CI) | p value | AUC (95% CI) | N | Median (min, max) NfL concentration (pg/ml) | β (95% CI) | p value | AUC (95% CI) |
| Controls | 161 | 126.3 (40.0, 402.7) | 0.00 (reference) | N/A | N/A | 161 | 7.3 (2.5, 66.1) | 0.00 (reference) | N/A | N/A |
| bvFTD |  |  |  |  |  |  |  |  |  |  |
| ≤ 5-years | 201 | 226.0 (48.2, 2544.5) | 0.61 (0.45, 0.78) | 1.92E-12 | 0.69 (0.63, 0.74) | 201 | 28.9 (4.7, 246.4) | 1.78 (1.58, 1.97) | 1.63E-52 | 0.87 (0.84, 0.91) |
| > 5-years | 107 | 213.8 (33.6, 1840.0) | 0.50 (0.30, 0.71) | 2.39E-06 | 0.62 (0.55, 0.69) | 107 | 20.8 (4.7, 163.9) | 1.32 (1.10, 1.55) | 3.80E-25 | 0.76 (0.70, 0.82) |
| nfvPPA |  |  |  |  |  |  |  |  |  |  |
| ≤ 5-years | 57 | 228.1 (88.9, 691.0) | 0.34 (0.12, 0.56) | 0.002 | 0.58 (0.49, 0.67) | 57 | 30.6 (11.7, 96.5) | 1.66 (1.44, 1.88) | 1.85E-34 | 0.85 (0.79, 0.92) |
| > 5-years | 19 | 303.0 (114.5, 665.5) | 0.60 (0.28, 0.92) | 2.57E-04 | 0.69 (0.55, 0.83) | 19 | 28.4 (13.5, 43.9) | 1.45 (1.16, 1.73) | 3.27E-19 | 0.92 (0.86, 0.99) |
| svPPA |  |  |  |  |  |  |  |  |  |  |
| ≤ 5-years | 43 | 207.9 (67.2, 1492.4) | 0.35 (0.11, 0.59) | 0.005 | 0.58 (0.48, 0.69) | 43 | 27.9 (11.6, 60.6) | 1.56 (1.36, 1.77) | 9.48E-35 | 0.92 (0.87, 0.97) |
| > 5-years | 40 | 230.9 (75.6, 988.0) | 0.40 (0.16, 0.64) | 0.001 | 0.61 (0.51, 0.70) | 40 | 31.4 (10.9, 77.7) | 1.59 (1.36, 1.82) | 2.60E-30 | 0.88 (0.81, 0.96) |
| CBS |  |  |  |  |  |  |  |  |  |  |
| ≤ 5-years | 63 | 234.5 (39.5, 2388.4) | 0.53 (0.29, 0.77) | 1.67E-05 | 0.62 (0.53, 0.71) | 63 | 27.3 (4.7, 124.7) | 1.41 (1.18, 1.63) | 1.05E-26 | 0.83 (0.76, 0.90) |
| > 5-years | 29 | 249.5 (111.1, 1084.1) | 0.63 (0.35, 0.91) | 1.43E-05 | 0.66 (0.53, 0.78) | 29 | 28.5 (6.4, 191.8) | 1.57 (1.27, 1.87) | 8.13E-20 | 0.83 (0.72, 0.93) |
| PSP-RS |  |  |  |  |  |  |  |  |  |  |
| ≤ 5-years | 95 | 215.6 (81.5, 637.7) | 0.25 (0.07, 0.44) | 0.007 | 0.56 (0.49, 0.63) | 95 | 26.3 (4.9, 112.0) | 1.48 (1.28, 1.69) | 1.53E-33 | 0.81 (0.75, 0.87) |
| > 5-years | 48 | 232.7 (85.3, 850.7) | 0.32 (0.07, 0.58) | 0.014 | 0.56 (0.46, 0.66) | 48 | 23.9 (8.8, 59.2) | 1.25 (1.01, 1.49) | 7.29E-20 | 0.78 (0.69, 0.86) |
| MBCI |  |  |  |  |  |  |  |  |  |  |
| ≤ 5-years | 55 | 131.9 (28.7, 595.9) | 0.03 (-0.16, 0.23) | 0.75 | 0.52 (0.42, 0.61) | 55 | 13.1 (2.7, 146.8) | 0.73 (0.49, 0.96) | 3.18E-09 | 0.69 (0.59, 0.79) |
| > 5-years | 12 | 155.3 (62.3, 384.0) | 0.23 (-0.10, 0.57) | 0.16 | 0.62 (0.45, 0.80) | 12 | 11.8 (2.5, 53.3) | 0.58 (0.26, 0.91) | 5.34E-04 | 0.72 (0.53, 0.91) |
|  |  |  |  |  |  |  |  |  |  |  |
| Presymptomatic | 127 | 119.9 (36.0, 674.0) | 0.00 (reference) | N/A | N/A | 127 | 7.5 (1.9, 68.2) | 0.00 (reference) | N/A | N/A |
| bvFTD |  |  |  |  |  |  |  |  |  |  |
| ≤ 5-years | 201 | 226.0 (48.2, 2544.5) | 0.46 (0.28, 0.65) | 1.55E-06 | 0.64 (0.58, 0.70) | 201 | 28.9 (4.7, 246.4) | 1.50 (1.27, 1.74) | 4.01E-30 | 0.82 (0.77, 0.86) |
| > 5-years | 107 | 213.8 (33.6, 1840.0) | 0.36 (0.14, 0.59) | 0.002 | 0.59 (0.51, 0.66) | 107 | 20.8 (4.7, 163.9) | 1.05 (0.76, 1.33) | 4.77E-12 | 0.70 (0.63, 0.76) |
| nfvPPA |  |  |  |  |  |  |  |  |  |  |
| ≤ 5-years | 57 | 228.1 (88.9, 691.0) | 0.26 (0.01, 0.50) | 0.044 | 0.56 (0.46, 0.65) | 57 | 30.6 (11.7, 96.5) | 1.36 (1.05, 1.68) | 3.47E-15 | 0.77 (0.69, 0.84) |
| > 5-years | 19 | 303.0 (114.5, 665.5) | 0.52 (0.17, 0.87) | 0.004 | 0.66 (0.52, 0.80) | 19 | 28.4 (13.5, 43.9) | 1.10 (0.68, 1.51) | 5.86E-07 | 0.82 (0.72, 0.91) |
| svPPA |  |  |  |  |  |  |  |  |  |  |
| ≤ 5-years | 43 | 207.9 (67.2, 1492.4) | 0.26 (-0.02, 0.53) | 0.065 | 0.56 (0.45, 0.66) | 43 | 27.9 (11.6, 60.6) | 1.27 (0.98, 1.57) | 5.67E-15 | 0.83 (0.76, 0.90) |
| > 5-years | 40 | 230.9 (75.6, 988.0) | 0.30 (0.03, 0.57) | 0.028 | 0.58 (0.47, 0.68) | 40 | 31.4 (10.9, 77.7) | 1.26 (0.93, 1.58) | 2.19E-12 | 0.80 (0.71, 0.88) |
| CBS |  |  |  |  |  |  |  |  |  |  |
| ≤ 5-years | 63 | 234.5 (39.5, 2388.4) | 0.46 (0.19, 0.72) | 0.001 | 0.59 (0.50, 0.69) | 63 | 27.3 (4.7, 124.7) | 1.11 (0.81, 1.41) | 7.91E-12 | 0.74 (0.66, 0.82) |
| > 5-years | 29 | 249.5 (111.1, 1084.1) | 0.56 (0.25, 0.87) | 4.28E-04 | 0.63 (0.50, 0.76) | 29 | 28.5 (6.4, 191.8) | 1.24 (0.83, 1.65) | 1.98E-08 | 0.73 (0.61, 0.84) |
| PSP-RS |  |  |  |  |  |  |  |  |  |  |
| ≤ 5-years | 95 | 215.6 (81.5, 637.7) | 0.17 (-0.04, 0.38) | 0.12 | 0.55 (0.47, 0.62) | 95 | 26.3 (4.9, 112.0) | 1.19 (0.91, 1.47) | 8.06E-15 | 0.73 (0.66, 0.79) |
| > 5-years | 48 | 232.7 (85.3, 850.7) | 0.27 (-0.01, 0.56) | 0.062 | 0.55 (0.45, 0.65) | 48 | 23.9 (8.8, 59.2) | 0.92 (0.58, 1.27) | 3.28E-07 | 0.69 (0.59, 0.78) |
| MBCI |  |  |  |  |  |  |  |  |  |  |
| ≤ 5-years | 55 | 131.9 (28.7, 595.9) | -0.09 (-0.30, 0.13) | 0.42 | 0.52 (0.43, 0.62) | 55 | 13.1 (2.7, 146.8) | 0.47 (0.17, 0.76) | 0.002 | 0.61 (0.51, 0.71) |
| > 5-years | 12 | 155.3 (62.3, 384.0) | 0.11 (-0.24, 0.46) | 0.53 | 0.57 (0.39, 0.74) | 12 | 11.8 (2.5, 53.3) | 0.33 (-0.12, 0.79) | 0.15 | 0.61 (0.43, 0.79) |
| β=regression coefficient; CI=confidence interval; AUC=area under the receiver operating curve. β values, 95% CIs, and p values result from linear regression models that were adjusted for age and sex. β values are interpreted as the difference in the mean GFAP or NfL concentration (on the base-2 logarithm scale) for the given phenotype group in comparison to controls. p values <0.0083 (comparisons vs. controls or vs. presymptomatic mutation carriers) are considered as statistically significant after applying a Bonferroni correction for multiple testing. | | | | | | | | | | |

**Table S12: Comparisons of baseline GFAP and NfL concentration between disease groups**

|  |  |  | Analysis adjusted for age, sex, and symptom duration | | | | |
| --- | --- | --- | --- | --- | --- | --- | --- |
| Marker/Disease group | N | Median (minimum, maximum) concentration | p value vs. nfvPPA | p value vs. svPPA | p value vs. CBS | p value vs. PSP-RS | p value vs. MBCI |
| GFAP |  |  |  |  |  |  |  |
| bvFTD | 308 | 222.8 (33.6, 2544.5) | 0.26 | 0.31 | 0.73 | 0.008 | 7.73E-05 |
| nfvPPA | 76 | 230.9 (88.9, 691.0) | ---- | 0.98 | 0.38 | 0.094 | 0.015 |
| svPPA | 83 | 224.4 (67.2, 1492.4) | ---- | ---- | 0.41 | 0.13 | 0.044 |
| CBS | 92 | 244.5 (39.5, 2388.4) | ---- | ---- | ---- | 0.018 | 6.84E-04 |
| PSP-RS | 143 | 223.9 (81.5, 850.7) | ---- | ---- | ---- | ---- | 0.079 |
| MBCI | 67 | 131.9 (28.7, 595.9) | ---- | ---- | ---- | ---- | ---- |
| NfL |  |  |  |  |  |  |  |
| bvFTD | 308 | 25.7 (4.7, 246.4) | 0.64 | 0.42 | 0.95 | 0.35 | 5.71E-09 |
| nfvPPA | 76 | 28.7 (11.7, 96.5) | ---- | 0.76 | 0.58 | 0.052 | 2.49E-06 |
| svPPA | 83 | 29.2 (10.9, 77.7) | ---- | ---- | 0.65 | 0.11 | 5.35E-07 |
| CBS | 92 | 28.3 (4.7, 191.8) | ---- | ---- | ---- | 0.23 | 1.42E-04 |
| PSP-RS | 143 | 25.7 (4.9, 112.0) | ---- | ---- | ---- | ---- | 2.33E-05 |
| MBCI | 67 | 12.6 (2.5, 146.8) | ---- | ---- | ---- | ---- | ---- |
| p values result from linear regression models that were adjusted for age, sex, and symptom duration. p values < 0.0033 are considered as statistically significant after applying a Bonferroni correction for multiple testing. | | | | | | | |

**Table S13: AUC values from models including both biomarkers when comparing asymptomatic groups to phenotype groups**

|  |  | AUC (95% CI) | |
| --- | --- | --- | --- |
| Phenotype group | N | Unadjusted analysis | Adjusting for age and sex |
| Controls | 161 | N/A | N/A |
| Presymptomatic | 127 | 0.52 (0.45, 0.59) | 0.60 (0.53, 0.67) |
| bvFTD | 308 | 0.92 (0.89, 0.94) | 0.86 (0.83, 0.89) |
| nfvPPA | 76 | 0.99 (0.97, 1.00) | 0.84 (0.78, 0.90) |
| svPPA | 83 | 0.98 (0.97, 1.00) | 0.87 (0.82, 0.93) |
| CBS | 92 | 0.94 (0.90, 0.98) | 0.80 (0.73, 0.86) |
| PSP-RS | 143 | 0.96 (0.94, 0.98) | 0.78 (0.73, 0.84) |
| MBCI | 67 | 0.74 (0.66, 0.82) | 0.71 (0.62, 0.80) |
|  |  |  |  |
| Presymptomatic | 127 | N/A | N/A |
| bvFTD | 308 | 0.88 (0.85, 0.92) | 0.80 (0.76, 0.84) |
| nfvPPA | 76 | 0.96 (0.93, 0.98) | 0.76 (0.69, 0.83) |
| svPPA | 83 | 0.95 (0.92, 0.98) | 0.80 (0.73, 0.86) |
| CBS | 92 | 0.91 (0.87, 0.95) | 0.72 (0.65, 0.79) |
| PSP-RS | 143 | 0.93 (0.90, 0.96) | 0.72 (0.66, 0.78) |
| MBCI | 67 | 0.70 (0.62, 0.78) | 0.64 (0.56, 0.73) |
| AUC=area under the receiver operating curve | | | |

**Table S14: Comparisons of baseline biomarkers between asymptomatic groups and mildly impaired phenotype groups – unadjusted analysis**

|  | Comparisons of GFAP | | | | | Comparisons of NfL | | | | |
| --- | --- | --- | --- | --- | --- | --- | --- | --- | --- | --- |
| Phenotype group | N | Median (min, max) GFAP concentration (pg/ml) | β (95% CI) | p value | AUC (95% CI) | N | Median (min, max) NfL concentration (pg/ml) | β (95% CI) | p value | AUC (95% CI) |
| Controls | 161 | 126.3 (40.0, 402.7) | 0.00 (reference) | N/A | N/A | 161 | 7.3 (2.5, 66.1) | 0.00 (reference) | N/A | N/A |
| Presymptomatic | 127 | 119.9 (36.0, 674.0) | 0.02 (-0.15, 0.18) | 0.85 | 0.49 (0.42, 0.56) | 127 | 7.5 (1.9, 68.2) | 0.10 (-0.08, 0.27) | 0.27 | 0.52 (0.45, 0.59) |
| bvFTD | 18 | 145.3 (48.2, 613.8) | 0.26 (-0.09, 0.61) | 0.15 | 0.58 (0.41, 0.75) | 18 | 15.5 (4.7, 125.4) | 1.21 (0.88, 1.55) | 2.56E-11 | 0.86 (0.75, 0.98) |
| nfvPPA | 31 | 226.6 (115.3, 691.0) | 0.91 (0.65, 1.17) | 8.72E-11 | 0.84 (0.77, 0.91) | 31 | 28.8 (12.4, 65.2) | 2.02 (1.78, 2.26) | 1.72E-38 | 0.99 (0.97, 1.00) |
| svPPA | 11 | 174.6 (75.6, 931.0) | 0.60 (0.17, 1.03) | 0.0071 | 0.69 (0.52, 0.87) | 11 | 17.6 (12.3, 44.6) | 1.40 (1.01, 1.78) | 1.89E-11 | 0.97 (0.94, 1.00) |
| CBS | 26 | 233.8 (39.5, 658.1) | 0.86 (0.56, 1.16) | 4.53E-08 | 0.81 (0.70, 0.91) | 26 | 22.8 (4.7, 124.7) | 1.61 (1.33, 1.90) | 1.74E-22 | 0.92 (0.83, 1.00) |
| PSP-RS | 23 | 239.8 (91.3, 637.7) | 0.90 (0.60, 1.20) | 1.40E-08 | 0.83 (0.75, 0.92) | 23 | 26.0 (6.6, 112.0) | 1.82 (1.52, 2.11) | 1.49E-25 | 0.95 (0.89, 1.01) |
| MBCI | 66 | 132.5 (28.7, 595.9) | 0.23 (0.02, 0.45) | 0.034 | 0.58 (0.49, 0.67) | 66 | 12.7 (2.5, 146.8) | 0.85 (0.60, 1.09) | 7.39E-11 | 0.73 (0.64, 0.81) |
|  |  |  |  |  |  |  |  |  |  |  |
| Presymptomatic | 127 | 119.9 (36.0, 674.0) | 0.00 (reference) | N/A | N/A | 127 | 7.5 (1.9, 68.2) | 0.00 (reference) | N/A | N/A |
| bvFTD | 18 | 145.3 (48.2, 613.8) | 0.24 (-0.12, 0.61) | 0.19 | 0.58 (0.41, 0.75) | 18 | 15.5 (4.7, 125.4) | 1.12 (0.67, 1.56) | 1.94E-06 | 0.82 (0.71, 0.92) |
| nfvPPA | 31 | 226.6 (115.3, 691.0) | 0.89 (0.63, 1.16) | 7.18E-10 | 0.84 (0.77, 0.91) | 31 | 28.8 (12.4, 65.2) | 1.92 (1.60, 2.25) | 4.11E-23 | 0.96 (0.93, 0.99) |
| svPPA | 11 | 174.6 (75.6, 931.0) | 0.58 (0.14, 1.03) | 0.011 | 0.69 (0.51, 0.86) | 11 | 17.6 (12.3, 44.6) | 1.30 (0.77, 1.82) | 2.66E-06 | 0.92 (0.87, 0.97) |
| CBS | 26 | 233.8 (39.5, 658.1) | 0.85 (0.54, 1.16) | 2.51E-07 | 0.80 (0.70, 0.91) | 26 | 22.8 (4.7, 124.7) | 1.52 (1.14, 1.89) | 3.06E-13 | 0.89 (0.81, 0.97) |
| PSP-RS | 23 | 239.8 (91.3, 637.7) | 0.89 (0.58, 1.19) | 6.52E-08 | 0.83 (0.74, 0.91) | 23 | 26.0 (6.6, 112.0) | 1.72 (1.33, 2.11) | 4.46E-15 | 0.92 (0.86, 0.98) |
| MBCI | 66 | 132.5 (28.7, 595.9) | 0.22 (-0.01, 0.45) | 0.062 | 0.59 (0.50, 0.68) | 66 | 12.7 (2.5, 146.8) | 0.75 (0.45, 1.05) | 1.85E-06 | 0.69 (0.61, 0.78) |
| β=regression coefficient; CI=confidence interval; AUC=area under the receiver operating curve. β values, 95% CIs, and p values result from unadjusted linear regression models. β values are interpreted as the difference in the mean GFAP or NfL concentration (on the base-2 logarithm scale) for the given phenotype group that includes only participants with a CDR®+NACC-FTLD global score of 0 or 0.5 in comparison to controls or to presymptomatic mutation carriers. p values <0.0071 (comparisons vs. controls) and <0.0083 (comparisons with presymptomatic mutation carriers) are considered as statistically significant after applying a Bonferroni correction for multiple testing. | | | | | | | | | | |

**Table S15: Comparisons of baseline biomarkers between asymptomatic groups and mildly impaired phenotype groups – adjusted analysis**

|  | Comparisons of GFAP | | | | | Comparisons of NfL | | | | |
| --- | --- | --- | --- | --- | --- | --- | --- | --- | --- | --- |
| Phenotype group | N | Median (min, max) GFAP concentration (pg/ml) | β (95% CI) | p value | AUC (95% CI) | N | Median (min, max) NfL concentration (pg/ml) | β (95% CI) | p value | AUC (95% CI) |
| Controls | 161 | 126.3 (40.0, 402.7) | 0.00 (reference) | N/A | N/A | 161 | 7.3 (2.5, 66.1) | 0.00 (reference) | N/A | N/A |
| Presymptomatic | 127 | 119.9 (36.0, 674.0) | 0.13 (-0.00, 0.27) | 0.057 | 0.55 (0.48, 0.62) | 127 | 7.5 (1.9, 68.2) | 0.22 (0.07, 0.37) | 0.004 | 0.60 (0.53, 0.66) |
| bvFTD | 18 | 145.3 (48.2, 613.8) | 0.05 (-0.25, 0.35) | 0.74 | 0.53 (0.35, 0.70) | 18 | 15.5 (4.7, 125.4) | 1.05 (0.76, 1.33) | 1.04E-11 | 0.87 (0.79, 0.95) |
| nfvPPA | 31 | 226.6 (115.3, 691.0) | 0.31 (0.07, 0.56) | 0.013 | 0.61 (0.50, 0.71) | 31 | 28.8 (12.4, 65.2) | 1.57 (1.32, 1.81) | 3.40E-27 | 0.91 (0.87, 0.96) |
| svPPA | 11 | 174.6 (75.6, 931.0) | 0.17 (-0.21, 0.55) | 0.38 | 0.52 (0.32, 0.71) | 11 | 17.6 (12.3, 44.6) | 1.05 (0.72, 1.38) | 3.10E-09 | 0.93 (0.87, 0.98) |
| CBS | 26 | 233.8 (39.5, 658.1) | 0.27 (0.00, 0.55) | 0.049 | 0.58 (0.45, 0.71) | 26 | 22.8 (4.7, 124.7) | 1.09 (0.83, 1.35) | 2.25E-14 | 0.86 (0.77, 0.95) |
| PSP-RS | 23 | 239.8 (91.3, 637.7) | 0.32 (0.05, 0.60) | 0.022 | 0.61 (0.49, 0.73) | 23 | 26.0 (6.6, 112.0) | 1.33 (1.05, 1.60) | 2.01E-17 | 0.85 (0.75, 0.95) |
| MBCI | 66 | 132.5 (28.7, 595.9) | 0.06 (-0.12, 0.24) | 0.54 | 0.53 (0.44, 0.61) | 66 | 12.7 (2.5, 146.8) | 0.68 (0.46, 0.90) | 4.17E-09 | 0.69 (0.60, 0.78) |
|  |  |  |  |  |  |  |  |  |  |  |
| Presymptomatic | 127 | 119.9 (36.0, 674.0) | 0.00 (reference) | N/A | N/A | 127 | 7.5 (1.9, 68.2) | 0.00 (reference) | N/A | N/A |
| bvFTD | 18 | 145.3 (48.2, 613.8) | -0.07 (-0.39, 0.25) | 0.67 | 0.51 (0.34, 0.68) | 18 | 15.5 (4.7, 125.4) | 0.75 (0.35, 1.14) | 2.58E-04 | 0.75 (0.64, 0.87) |
| nfvPPA | 31 | 226.6 (115.3, 691.0) | 0.22 (-0.05, 0.50) | 0.11 | 0.58 (0.47, 0.68) | 31 | 28.8 (12.4, 65.2) | 1.26 (0.90, 1.62) | 8.41E-11 | 0.78 (0.70, 0.87) |
| svPPA | 11 | 174.6 (75.6, 931.0) | 0.09 (-0.32, 0.51) | 0.66 | 0.51 (0.31, 0.71) | 11 | 17.6 (12.3, 44.6) | 0.73 (0.25, 1.22) | 0.003 | 0.79 (0.68, 0.90) |
| CBS | 26 | 233.8 (39.5, 658.1) | 0.18 (-0.12, 0.49) | 0.23 | 0.56 (0.43, 0.69) | 26 | 22.8 (4.7, 124.7) | 0.75 (0.38, 1.12) | 1.06E-04 | 0.73 (0.63, 0.83) |
| PSP-RS | 23 | 239.8 (91.3, 637.7) | 0.24 (-0.06, 0.54) | 0.12 | 0.58 (0.46, 0.70) | 23 | 26.0 (6.6, 112.0) | 0.99 (0.59, 1.38) | 2.58E-06 | 0.74 (0.62, 0.86) |
| MBCI | 66 | 132.5 (28.7, 595.9) | -0.07 (-0.27, 0.13) | 0.52 | 0.49 (0.40, 0.58) | 66 | 12.7 (2.5, 146.8) | 0.43 (0.15, 0.71) | 0.003 | 0.60 (0.51, 0.69) |
| β=regression coefficient; CI=confidence interval; AUC=area under the receiver operating curve. β values, 95% CIs, and p values result from linear regression models that were adjusted for age and sex. β values are interpreted as the difference in the mean GFAP or NfL concentration (on the base-2 logarithm scale) for the given phenotype group that includes only participants with a CDR®+NACC-FTLD global score of 0 or 0.5 in comparison to controls or to presymptomatic mutation carriers. p values <0.0071 (comparisons vs. controls) and <0.0083 (comparisons with presymptomatic mutation carriers) are considered as statistically significant after applying a Bonferroni correction for multiple testing. | | | | | | | | | | |

**Table S16:** **Comparison of baseline biomarkers or their rates of change between controls and phenoconverters or non-converters**

|  |  |  | Unadjusted analysis | | | Adjusting for age and sex | | |
| --- | --- | --- | --- | --- | --- | --- | --- | --- |
| Marker/Mutation Status | N | Median (minimum, maximum) GFAP or NfL concentration (pg/ml) | β (95% CI) | p value | AUC (95% CI) | β (95% CI) | p value | AUC (95% CI) |
| Baseline biomarkers |  |  |  |  |  |  |  |  |
|  |  |  |  |  |  |  |  |  |
| Baseline GFAP |  |  |  |  |  |  |  |  |
| Controls | 161 | 126.3 (40.0, 402.7) | 0.00 (reference) | N/A | N/A | 0.00 (reference) | N/A | N/A |
| Presymptomatic non-converters | 74 | 126.5 (36.0, 468.1) | -0.02 (-0.21, 0.17) | 0.86 | 0.51 (0.43, 0.59) | 0.15 (-0.01, 0.31) | 0.071 | 0.57 (0.49, 0.65) |
| Presymptomatic phenoconverters | 29 | 123.0 (62.9, 674.0) | 0.26 (-0.01, 0.54) | 0.063 | 0.56 (0.43, 0.68) | 0.32 (0.09, 0.54) | 0.006 | 0.61 (0.49, 0.73) |
|  |  |  |  |  |  |  |  |  |
| Presymptomatic non-converters | 74 | 126.5 (36.0, 468.1) | 0.00 (reference) | N/A | N/A | 0.00 (reference) | N/A | N/A |
| Presymptomatic phenoconverters | 29 | 123.0 (62.9, 674.0) | 0.28 (-0.03, 0.59) | 0.075 | 0.55 (0.42, 0.69) | 0.17 (-0.08, 0.43) | 0.19 | 0.54 (0.41, 0.68) |
|  |  |  |  |  |  |  |  |  |
| Baseline NfL |  |  |  |  |  |  |  |  |
| Controls | 161 | 7.3 (2.5, 66.1) | 0.00 (reference) | N/A | N/A | 0.00 (reference) | N/A | N/A |
| Presymptomatic non-converters | 74 | 7.4 (1.9, 68.2) | -0.00 (-0.20, 0.20) | 0.98 | 0.50 (0.42, 0.59) | 0.16 (-0.01, 0.33) | 0.069 | 0.57 (0.48, 0.65) |
| Presymptomatic phenoconverters | 29 | 9.6 (4.3, 65.9) | 0.59 (0.30, 0.87) | 7.75E-05 | 0.69 (0.57, 0.81) | 0.65 (0.40, 0.89) | 3.41E-07 | 0.77 (0.66, 0.87) |
|  |  |  |  |  |  |  |  |  |
| Presymptomatic non-converters | 74 | 7.4 (1.9, 68.2) | 0.00 (reference) | N/A | N/A | 0.00 (reference) | N/A | N/A |
| Presymptomatic phenoconverters | 29 | 9.6 (4.3, 65.9) | 0.59 (0.22, 0.96) | 0.002 | 0.68 (0.56, 0.80) | 0.48 (0.15, 0.80) | 0.005 | 0.68 (0.57, 0.80) |
|  |  |  |  |  |  |  |  |  |
| Rates of biomarker change |  |  |  |  |  |  |  |  |
|  |  |  |  |  |  |  |  |  |
| Rate of GFAP change |  |  |  |  |  |  |  |  |
| Controls | 89 | 4.0 (-87.6, 66.8) | 0.00 (reference) | N/A | N/A | 0.00 (reference) | N/A | N/A |
| Presymptomatic non-converters | 53 | 6.5 (-32.0, 69.2) | 14.07 (-7.21, 35.35) | 0.19 | 0.56 (0.46, 0.66) | 15.20 (-7.04, 37.45) | 0.18 | 0.56 (0.46, 0.66) |
| Presymptomatic phenoconverters | 26 | 13.8 (-56.5, 73.7) | 34.75 (6.41, 63.09) | 0.017 | 0.65 (0.51, 0.79) | 35.02 (6.32, 63.72) | 0.017 | 0.65 (0.51, 0.79) |
|  |  |  |  |  |  |  |  |  |
| Presymptomatic non-converters | 53 | 6.5 (-32.0, 69.2) | 0.00 (reference) | N/A | N/A | 0.00 (reference) | N/A | N/A |
| Presymptomatic phenoconverters | 26 | 13.8 (-56.5, 73.7) | 20.68 (-11.91, 53.27) | 0.21 | 0.59 (0.45, 0.74) | 18.69 (-14.97, 52.35) | 0.27 | 0.58 (0.44, 0.73) |
|  |  |  |  |  |  |  |  |  |
| Rate of NfL change |  |  |  |  |  |  |  |  |
| Controls | 89 | 0.2 (-8.9, 4.9) | 0.00 (reference) | N/A | N/A | 0.00 (reference) | N/A | N/A |
| Presymptomatic non-converters | 53 | 0.4 (-4.0, 11.3) | 14.46 (-4.64, 33.57) | 0.14 | 0.57 (0.47, 0.68) | 15.83 (-3.97, 35.64) | 0.12 | 0.58 (0.47, 0.68) |
| Presymptomatic phenoconverters | 26 | 1.3 (-0.5, 31.0) | 69.72 (44.92, 94.52) | 1.75E-07 | 0.78 (0.67, 0.90) | 68.86 (43.99, 93.74) | 2.62E-07 | 0.78 (0.66, 0.90) |
|  |  |  |  |  |  |  |  |  |
| Presymptomatic non-converters | 53 | 0.4 (-4.0, 11.3) | 0.00 (reference) | N/A | N/A | 0.00 (reference) | N/A | N/A |
| Presymptomatic phenoconverters | 26 | 1.3 (-0.5, 31.0) | 55.26 (24.87, 85.64) | 5.23E-04 | 0.74 (0.61, 0.86) | 48.37 (17.77, 78.97) | 0.002 | 0.70 (0.57, 0.82) |
| β=regression coefficient; CI=confidence interval; AUC=area under the receiver operating curve. β values, 95% CIs, and p values result from linear regression models. For baseline biomarkers, β values are interpreted as the difference in the mean GFAP or NfL concentration (on the base-2 logarithm scale) in comparison to the reference group. For rates of biomarker change, β values are interpreted as the difference in the mean rank of rate of change per year in GFAP or NfL concentration for the given group in comparison to the reference group. p values < 0.0167 are considered statistically significant after applying a Bonferroni correction for multiple testing. | | | | | | | | |

**Table S17: AUC values from models including both biomarkers when comparing controls to phenoconverters or non-converters**

|  |  | AUC (95% CI) | |
| --- | --- | --- | --- |
| Phenotype group | N | Unadjusted analysis | Adjusting for age and sex |
| Controls | 161 | N/A | N/A |
| Presymptomatic non-converters | 74 | 0.51 (0.43, 0.59) | 0.59 (0.51, 0.67) |
| Presymptomatic phenoconverters | 29 | 0.69 (0.57, 0.81) | 0.75 (0.64, 0.86) |
|  |  |  |  |
| Presymptomatic non-converters | 74 | N/A | N/A |
| Presymptomatic phenoconverters | 29 | 0.68 (0.56, 0.80) | 0.68 (0.56, 0.80) |
| AUC=area under the receiver operating curve | | | |

**Table S18: Comparison of baseline biomarkers or their rates of change by mutation status in asymptomatic groups**

|  |  |  | Unadjusted analysis | | Adjusting for age and sex | |
| --- | --- | --- | --- | --- | --- | --- |
| Marker/Disease group/Mutation Status | N | Median (minimum, maximum) concentration | β (95% CI) | p value | β (95% CI) | p value |
| Baseline biomarkers |  |  |  |  |  |  |
|  |  |  |  |  |  |  |
| Baseline GFAP |  |  |  |  |  |  |
| Controls | 161 | 126.3 (40.0, 402.7) | 0.00 (reference) | N/A | 0.00 (reference) | N/A |
| *C9orf72* mutation | 60 | 119.1 (36.0, 468.1) | 0.00 (-0.20, 0.21) | 0.98 | 0.13 (-0.05, 0.30) | 0.15 |
| *GRN* mutation | 34 | 129.2 (62.6, 674.0) | 0.14 (-0.12, 0.40) | 0.29 | 0.08 (-0.14, 0.30) | 0.49 |
| *MAPT* mutation | 31 | 119.9 (54.1, 271.7) | -0.08 (-0.35, 0.18) | 0.54 | 0.19 (-0.03, 0.42) | 0.091 |
| Baseline NfL |  |  |  |  |  |  |
| Controls | 161 | 7.3 (2.5, 66.1) | 0.00 (reference) | N/A | 0.00 (reference) | N/A |
| *C9orf72* mutation | 60 | 7.7 (1.9, 68.2) | 0.11 (-0.11, 0.33) | 0.35 | 0.23 (0.05, 0.42) | 0.013 |
| *GRN* mutation | 34 | 7.0 (3.3, 65.9) | 0.14 (-0.13, 0.42) | 0.30 | 0.08 (-0.16, 0.32) | 0.50 |
| *MAPT* mutation | 31 | 8.1 (3.4, 30.0) | -0.02 (-0.30, 0.27) | 0.91 | 0.27 (0.03, 0.51) | 0.026 |
|  |  |  |  |  |  |  |
| Rates of biomarker change |  |  |  |  |  |  |
|  |  |  |  |  |  |  |
| Rate of GFAP change |  |  |  |  |  |  |
| Controls | 89 | 4.0 (-87.6, 66.8) | 0.00 (reference) | N/A | 0.00 (reference) | N/A |
| *C9orf72* mutation | 36 | 10.5 (-17.5, 41.0) | 22.32 (-2.17, 46.81) | 0.074 | 23.48 (-1.94, 48.90) | 0.070 |
| *GRN* mutation | 18 | 2.7 (-56.5, 73.7) | 4.65 (-28.01, 37.31) | 0.78 | 0.80 (-34.07, 35.67) | 0.96 |
| *MAPT* mutation | 24 | 6.0 (-7.3, 44.5) | 26.64 (-1.02, 54.29) | 0.059 | 23.56 (-6.38, 53.50) | 0.12 |
|  |  |  |  |  |  |  |
| Rate of NfL change |  |  |  |  |  |  |
| Controls | 89 | 0.2 (-8.9, 4.9) | 0.00 (reference) | N/A | 0.00 (reference) | N/A |
| *C9orf72* mutation | 36 | 0.4 (-4.0, 11.3) | 16.37 (-6.03, 38.78) | 0.15 | 22.70 (0.02, 45.38) | 0.050 |
| *GRN* mutation | 18 | 0.5 (-1.6, 31.0) | 48.90 (20.71, 77.10) | 8.39E-04 | 41.98 (12.10, 71.86) | 0.006 |
| *MAPT* mutation | 24 | 0.6 (-0.8, 7.1) | 39.85 (14.96, 64.73) | 0.002 | 38.06 (11.26, 64.85) | 0.006 |
| β=regression coefficient; CI=confidence interval. β values, 95% CIs, and p values result from linear regression models. For baseline biomarkers, β values are interpreted as the difference in the mean GFAP or NfL concentration (on the base-2 logarithm scale) in comparison to the reference group. For rates of change in biomarkers, β values are interpreted as the difference in the mean rank of rate of change per year in GFAP or NfL concentration for the given group in comparison to the reference group. p values < 0.0167 are considered statistically significant after applying a Bonferroni correction for multiple testing. | | | | | | |

**Table S19: Comparison of rate of change in GFAP and NfL concentrations between controls and phenotype groups**

|  |  |  | Unadjusted analysis | | | Adjusting for age and sex | | |
| --- | --- | --- | --- | --- | --- | --- | --- | --- |
| Disease group/Mutation Status | N | Median (minimum, maximum) rate of change per year in GFAP or NfL concentration (pg/ml) | β (95% CI) | p value | AUC (95% CI) | β (95% CI) | p value | AUC (95% CI) |
| Rate of change in GFAP |  |  |  |  |  |  |  |  |
| Controls | 89 | 4.0 (-87.6, 66.8) | 0.00 (reference) | N/A | N/A | 0.00 (reference) | N/A | N/A |
| All pre-symptomatic mutation carriers | 79 | 6.9 (-56.5, 73.7) | 20.87 (1.21, 40.54) | 0.038 | 0.59 (0.50, 0.68) | 22.05 (1.92, 42.18) | 0.032 | 0.59 (0.51, 0.68) |
| Non-converters | 53 | 6.5 (-32.0, 69.2) | 14.07 (-7.21, 35.35) | 0.19 | 0.56 (0.46, 0.66) | 15.20 (-7.04, 37.45) | 0.18 | 0.56 (0.46, 0.66) |
| Phenoconverters | 26 | 13.8 (-56.5, 73.7) | 34.75 (6.41, 63.09) | 0.017 | 0.65 (0.51, 0.79) | 35.02 (6.32, 63.72) | 0.017 | 0.65 (0.51, 0.79) |
| MBCI | 21 | 18.0 (-5.6, 68.3) | 48.69 (18.17, 79.21) | 0.002 | 0.69 (0.56, 0.84) | 50.45 (18.85, 82.04) | 0.002 | 0.69 (0.55, 0.84) |
| bvFTD | 49 | 11.6 (-67.2, 449.4) | 44.64 (20.22, 69.05) | 4.21E-04 | 0.67 (0.56, 0.78) | 22.05 (1.92, 42.18) | 0.032 | 0.68 (0.57, 0.78) |
| nfvPPA and svPPA | 8 | 9.0 (-85.9, 173.3) | 19.85 (-28.48, 68.17) | 0.42 | 0.53 (0.20, 0.86) | 26.27 (-29.56, 82.09) | 0.35 | 0.54 (0.21, 0.87) |
| CBS and PSP-RS | 13 | 1.8 (-69.0, 102.1) | 24.36 (-15.65, 64.37) | 0.23 | 0.55 (0.31, 0.79) | 37.79 (-5.34, 80.92) | 0.085 | 0.58 (0.36, 0.80) |
|  |  |  |  |  |  |  |  |  |
| Rate of change in NfL |  |  |  |  |  |  |  |  |
| Controls | 89 | 0.2 (-8.9, 4.9) | 0.00 (reference) | N/A | N/A | 0.00 (reference) | N/A | N/A |
| All pre-symptomatic mutation carriers | 79 | 0.5 (-4.0, 31.0) | 32.65 (14.22, 51.07) | 6.00E-04 | 0.64 (0.56, 0.73) | 35.94 (17.37, 54.51) | 1.89E-04 | 0.66 (0.57, 0.74) |
| Non-converters | 53 | 0.4 (-4.0, 11.3) | 14.46 (-4.64, 33.57) | 0.14 | 0.57 (0.47, 0.68) | 15.83 (-3.97, 35.64) | 0.12 | 0.58 (0.47, 0.68) |
| Phenoconverters | 26 | 1.3 (-0.5, 31.0) | 69.72 (44.92, 94.52) | 1.75E-07 | 0.78 (0.67, 0.90) | 68.86 (43.99, 93.74) | 2.62E-07 | 0.78 (0.66, 0.90) |
| MBCI | 21 | 1.0 (-3.1, 54.6) | 47.80 (18.29, 77.31) | 0.002 | 0.66 (0.48, 0.83) | 46.78 (16.35, 77.20) | 0.003 | 0.65 (0.47, 0.83) |
| bvFTD | 49 | 1.3 (-3.8, 48.9) | 60.96 (38.43, 83.48) | 3.59E-07 | 0.73 (0.63, 0.84) | 61.71 (37.81, 85.60) | 1.10E-06 | 0.72 (0.61, 0.83) |
| nfvPPA and svPPA | 8 | 8.8 (1.0, 41.9) | 127.30 (90.04, 164.57) | 1.01E-09 | 0.98 (0.94, 1.01) | 125.46 (83.25, 167.66) | 5.78E-08 | 0.95 (0.89, 1.00) |
| CBS and PSP-RS | 13 | 4.0 (-15.5, 18.6) | 57.41 (21.56, 93.26) | 0.002 | 0.68 (0.43, 0.93) | 59.79 (20.72, 98.86) | 0.003 | 0.67 (0.41, 0.92) |
| β=regression coefficient; CI=confidence interval; AUC=area under the receiver operating curve. β values, 95% CIs, and p values result from linear regression models. β values are interpreted as the difference in the mean rank of rate of change per year in GFAP or NfL concentration for the given group in comparison to controls. p values < 0.0071 are considered statistically significant after applying a Bonferroni correction for multiple testing. | | | | | | | | |

**Table S20: Characteristics of disease severity indicators according to disease group**

|  | Median (minimum, maximum)  or number of participants | | | | | |
| --- | --- | --- | --- | --- | --- | --- |
| Variable | bvFTD (N=308) | nfvPPA (N=76) | svPPA (N=83) | CBS (N=92) | PSP-RS (N=143) | MBCI (N=67) |
| CDR®+NACC-FTLDsb | 8.5 (0.5, 24.0) | 3.5 (0.5, 24.0) | 6.3 (1.5, 20.0) | 5.0 (0.0, 24.0) | 6.5 (0.0, 21.0) | 1.5 (0.0, 8.5) |
| Unknown | 3 | 0 | 1 | 0 | 18 | 0 |
| MoCA | 20 (1, 30) | 22 (1, 30) | 16.5 (0, 27) | 23 (1, 30) | 22 (2, 30) | 24 (14, 30) |
| Unknown | 31 | 13 | 5 | 19 | 22 | 0 |
| NAT | 7 (0, 10) | 7 (0, 10) | 9 (1, 10) | 9 (0, 10) | 9 (0, 10) | 10 (2, 10) |
| Unknown | 83 | 18 | 15 | 33 | 60 | 10 |
| MINT | 27 (0, 32) | 29 (0, 32) | 5 (0, 29) | 30 (17, 32) | 29 (5, 32) | 29 (1, 32) |
| Unknown | 49 | 16 | 18 | 15 | 25 | 1 |
| Category fluency | 19 (0, 53) | 19 (0, 46) | 10 (0, 38) | 22 (0, 48) | 17 (0, 40) | 32 (4, 48) |
| Unknown | 46 | 16 | 10 | 12 | 20 | 1 |
| Phonemic fluency | 13 (0, 45) | 11 (0, 28) | 15 (0, 37) | 17 (3, 39) | 12 (0, 36) | 23 (5, 41) |
| Unknown | 44 | 18 | 6 | 14 | 23 | 1 |
| Digit span backward | 4 (0, 12) | 4 (0, 10) | 6 (0, 12) | 4 (0, 11) | 4 (0, 10) | 6 (2, 12) |
| Unknown | 42 | 15 | 11 | 14 | 26 | 0 |
| Trails B | 111 (29, 300) | 138 (25, 300) | 101 (37, 300) | 167 (32, 300) | 220 (37, 300) | 89 (31, 300) |
| Unknown | 125 | 22 | 11 | 35 | 58 | 2 |
| CDR®+NACC-FTLDsb = CDR® Dementia Staging + National Alzheimer’s Disease Coordinating Center Frontotemporal Lobar Degeneration sum of boxes; MoCA = Montreal Cognitive Assessment; NAT = Northwestern Anagram Test; MINT = Multilingual Naming Test; Trails B = Trail Marking Test Part B. | | | | | | |

**Table S21: Associations of baseline GFAP with baseline disease indicators in FTD syndromes and MBCI – unadjusted analysis**

|  | Association between GFAP and: | | | | | | | |
| --- | --- | --- | --- | --- | --- | --- | --- | --- |
| Phenotype group | CDR®+NACC-FTLDsb | MoCA | NAT | MINT | Category fluency | Phonemic fluency | Digit span backward | Trails B |
| All FTD groups | N=680 | N=612 | N=493 | N=579 | N=598 | N=597 | N=594 | N=451 |
| β (95% CI) | 1.26 (0.84, 1.68) | -1.99 (-2.60, -1.37) | -0.38 (-0.67, -0.09) | -0.97 (-1.85, -0.08) | -2.36 (-3.36, -1.36) | -1.64 (-2.51, -0.78) | -0.73 (-0.97, -0.49) | 22.82 (12.82, 32.82) |
| p value | **8.45E-09** | **4.56E-10** | 0.011 | 0.032 | **4.66E-06** | **2.17E-04** | **4.22E-09** | **9.33E-06** |
|  |  |  |  |  |  |  |  |  |
| bvFTD | N=305 | N=277 | N=225 | N=259 | N=262 | N=264 | N=266 | N=183 |
| β (95% CI) | 1.73 (1.16, 2.30) | -2.54 (-3.42, -1.67) | -0.58 (-1.00, -0.17) | -1.67 (-2.77, -0.57) | -4.06 (-5.50, -2.61) | -2.74 (-4.08, -1.41) | -0.90 (-1.28, -0.52) | 13.05 (-1.87, 27.97) |
| p value | **6.58E-09** | **3.13E-08** | **0.0061** | **0.003** | **8.24E-08** | **6.85E-05** | **3.98E-06** | 0.086 |
|  |  |  |  |  |  |  |  |  |
| nfvPPA | N=76 | N=63 | N=58 | N=60 | N=60 | N=58 | N=61 | N=54 |
| β (95% CI) | 1.19 (-0.15, 2.52) | -1.58 (-4.17, 1.01) | -0.21 (-1.45, 1.04) | -1.02 (-3.77, 1.73) | -0.82 (-4.89, 3.26) | -2.21 (-4.98, 0.56) | -0.49 (-1.34, 0.35) | 30.15 (-4.10, 64.40) |
| p value | 0.081 | 0.23 | 0.74 | 0.46 | 0.69 | 0.12 | 0.25 | 0.083 |
|  |  |  |  |  |  |  |  |  |
| svPPA | N=82 | N=78 | N=68 | N=65 | N=73 | N=77 | N=72 | N=72 |
| β (95% CI) | 0.30 (-0.60, 1.20) | -1.02 (-2.58, 0.54) | -0.16 (-0.86, 0.54) | 0.56 (-1.83, 2.96) | -0.69 (-2.92, 1.53) | 0.22 (-1.97, 2.42) | -0.84 (-1.50, -0.18) | 42.54 (23.48, 61.59) |
| p value | 0.51 | 0.20 | 0.65 | 0.64 | 0.54 | 0.84 | 0.013 | **3.15E-05** |
|  |  |  |  |  |  |  |  |  |
| CBS | N=92 | N=73 | N=59 | N=77 | N=80 | N=78 | N=78 | N=57 |
| β (95% CI) | 1.28 (0.16, 2.40) | -1.57 (-3.14, 0.00) | -0.15 (-0.88, 0.59) | -0.32 (-1.12, 0.48) | -0.83 (-3.42, 1.76) | -1.17 (-3.36, 1.01) | -0.64 (-1.18, -0.11) | 30.63 (7.92, 53.35) |
| p value | 0.025 | 0.051 | 0.69 | 0.43 | 0.53 | 0.29 | 0.019 | 0.009 |
|  |  |  |  |  |  |  |  |  |
| PSP-RS | N=125 | N=121 | N=83 | N=118 | N=123 | N=120 | N=117 | N=85 |
| β (95% CI) | 1.49 (0.29, 2.68) | -2.43 (-3.81, -1.05) | -0.50 (-1.31, 0.31) | -1.55 (-2.90, -0.20) | -1.36 (-3.51, 0.80) | -0.12 (-2.21, 1.98) | -0.20 (-0.72, 0.33) | -0.03 (-31.86, 31.80) |
| p value | 0.015 | **6.66E-04** | 0.22 | 0.025 | 0.21 | 0.91 | 0.46 | 1.00 |
|  |  |  |  |  |  |  |  |  |
| MBCI | N=67 | N=67 | N=57 | N=66 | N=66 | N=66 | N=67 | N=65 |
| β (95% CI) | 0.18 (-0.19, 0.56) | -0.59 (-1.59, 0.41) | -0.08 (-0.70, 0.54) | -0.70 (-1.95, 0.55) | -2.90 (-5.36, -0.44) | -0.75 (-2.84, 1.33) | -0.00 (-0.59, 0.58) | 20.12 (4.15, 36.10) |
| p value | 0.33 | 0.24 | 0.80 | 0.27 | 0.022 | 0.47 | 0.99 | 0.014 |
|  |  |  |  |  |  |  |  |  |
| β=regression coefficient; CI=confidence interval. β values, 95% CIs, and p values result from unadjusted linear regression models. β values are interpreted as the change in the mean value of the given disease indicator for each doubling of GFAP concentration. p values < 0.00625 are considered as statistically significant after applying a Bonferroni correction for multiple testing and are shown in bold. The All FTD group includes patients with bvFTD, nfvPPA, svPPA, CBS or PSP-RS. | | | | | | | | |

**Table S22: Associations of baseline GFAP with baseline disease indicators in FTD syndromes and MBCI – adjusted analysis**

|  | Association between GFAP and: | | | | | | | |
| --- | --- | --- | --- | --- | --- | --- | --- | --- |
| Phenotype group | CDR®+NACC-FTLDsb | MoCA | NAT | MINT | Category fluency | Phonemic fluency | Digit span backward | Trails B |
| All FTD groups | N=680 | N=612 | N=493 | N=579 | N=598 | N=597 | N=594 | N=451 |
| β (95% CI) | 1.44 (1.00, 1.88) | -2.21 (-2.87, -1.55) | -0.52 (-0.83, -0.20) | -1.00 (-1.95, -0.05) | -2.34 (-3.44, -1.24) | -1.81 (-2.75, -0.87) | -0.73 (-0.99, -0.46) | 12.70 (2.03, 23.36) |
| p value | **2.27E-10** | **1.09E-10** | **0.001** | 0.040 | **3.25E-05** | **1.68E-04** | **7.80E-08** | 0.020 |
|  |  |  |  |  |  |  |  |  |
| bvFTD | N=305 | N=277 | N=225 | N=259 | N=262 | N=264 | N=266 | N=183 |
| β (95% CI) | 1.43 (0.81, 2.04) | -2.25 (-3.22, -1.27) | -0.74 (-1.21, -0.28) | -0.92 (-2.14, 0.30) | -3.84 (-5.50, -2.18) | -3.10 (-4.56, -1.64) | -0.85 (-1.27, -0.43) | 7.52 (-9.04, 24.09) |
| p value | **6.49E-06** | **8.58E-06** | **0.002** | 0.14 | **8.39E-06** | **4.08E-05** | **9.59E-05** | 0.37 |
|  |  |  |  |  |  |  |  |  |
| nfvPPA | N=76 | N=63 | N=58 | N=60 | N=60 | N=58 | N=61 | N=54 |
| β (95% CI) | 1.41 (-0.13, 2.96) | -2.18 (-5.00, 0.64) | -0.72 (-2.33, 0.88) | 0.32 (-2.80, 3.44) | -1.44 (-6.23, 3.36) | -4.31 (-7.44, -1.19) | -0.68 (-1.63, 0.28) | 26.47 (-18.27, 71.20) |
| p value | 0.072 | 0.13 | 0.37 | 0.84 | 0.55 | 0.008 | 0.16 | 0.24 |
|  |  |  |  |  |  |  |  |  |
| svPPA | N=82 | N=78 | N=68 | N=65 | N=73 | N=77 | N=72 | N=72 |
| β (95% CI) | 0.43 (-0.61, 1.47) | -1.44 (-3.19, 0.30) | -0.30 (-1.11, 0.51) | -0.49 (-3.22, 2.24) | -1.14 (-3.76, 1.47) | -0.48 (-2.93, 1.98) | -0.85 (-1.61, -0.10) | 42.62 (20.77, 64.46) |
| p value | 0.42 | 0.10 | 0.46 | 0.72 | 0.39 | 0.70 | 0.027 | **2.32E-04** |
|  |  |  |  |  |  |  |  |  |
| CBS | N=92 | N=73 | N=59 | N=77 | N=80 | N=78 | N=78 | N=57 |
| β (95% CI) | 1.32 (0.18, 2.46) | -1.59 (-3.17, -0.01) | -0.08 (-0.76, 0.60) | -0.27 (-1.09, 0.54) | -0.71 (-3.32, 1.89) | -0.66 (-2.83, 1.51) | -0.66 (-1.22, -0.09) | 23.28 (-0.07, 46.63) |
| p value | 0.024 | 0.049 | 0.82 | 0.51 | 0.59 | 0.54 | 0.025 | 0.051 |
|  |  |  |  |  |  |  |  |  |
| PSP-RS | N=125 | N=121 | N=83 | N=118 | N=123 | N=120 | N=117 | N=85 |
| β (95% CI) | 1.64 (0.39, 2.88) | -2.44 (-3.91, -0.98) | -0.46 (-1.31, 0.38) | -1.90 (-3.33, -0.47) | -1.30 (-3.54, 0.95) | -0.56 (-2.78, 1.66) | -0.17 (-0.73, 0.39) | -16.41 (-47.97, 15.14) |
| p value | 0.011 | **0.001** | 0.28 | 0.010 | 0.25 | 0.62 | 0.56 | 0.30 |
|  |  |  |  |  |  |  |  |  |
| MBCI | N=67 | N=67 | N=57 | N=66 | N=66 | N=66 | N=67 | N=65 |
| β (95% CI) | 0.17 (-0.27, 0.60) | -0.46 (-1.68, 0.76) | 0.04 (-0.70, 0.79) | -0.92 (-2.47, 0.62) | -3.88 (-6.99, -0.77) | -1.19 (-3.70, 1.33) | -0.24 (-0.98, 0.50) | 6.99 (-11.74, 25.71) |
| p value | 0.45 | 0.46 | 0.91 | 0.24 | 0.015 | 0.35 | 0.51 | 0.46 |
| β=regression coefficient; CI=confidence interval. β values, 95% CIs, and p values result from linear regression models that were adjusted for age, sex, symptom duration, and years of education. β values are interpreted as the change in the mean value of the given disease indicator for each doubling of GFAP concentration. p values < 0.00625 are considered as statistically significant after applying a Bonferroni correction for multiple testing and are shown in bold. The All FTD group includes patients with bvFTD, nfvPPA, svPPA, CBS or PSP-RS. | | | | | | | | |

**Table S23: Associations of baseline NfL with baseline disease indicators in FTD syndromes and MBCI – unadjusted analysis**

|  | Association between NfL and: | | | | | | | |
| --- | --- | --- | --- | --- | --- | --- | --- | --- |
| Phenotype group | CDR®+NACC-FTLDsb | MoCA | NAT | MINT | Category fluency | Phonemic fluency | Digit span backward | Trails B |
| All FTD groups | N=680 | N=612 | N=493 | N=579 | N=598 | N=597 | N=594 | N=451 |
| β (95% CI) | 1.15 (0.76, 1.54) | -2.07 (-2.62, -1.53) | -0.75 (-1.01, -0.49) | -2.05 (-2.82, -1.27) | -3.91 (-4.78, -3.04) | -3.93 (-4.65, -3.21) | -0.82 (-1.03, -0.61) | 22.28 (13.35, 31.20) |
| p value | **8.64E-09** | **3.58E-13** | **1.63E-08** | **3.11E-07** | **1.07E-17** | **1.94E-24** | **8.28E-14** | **1.31E-06** |
|  |  |  |  |  |  |  |  |  |
| bvFTD | N=305 | N=277 | N=225 | N=259 | N=262 | N=264 | N=266 | N=183 |
| β (95% CI) | 1.39 (0.89, 1.89) | -2.46 (-3.20, -1.72) | -0.89 (-1.23, -0.55) | -2.48 (-3.37, -1.59) | -5.22 (-6.37, -4.08) | -4.81 (-5.80, -3.82) | -0.97 (-1.28, -0.66) | 18.17 (6.33, 30.01) |
| p value | **8.48E-08** | **3.00E-10** | **5.53E-07** | **9.36E-08** | **6.46E-17** | **8.53E-19** | **1.77E-09** | **0.003** |
|  |  |  |  |  |  |  |  |  |
| nfvPPA | N=76 | N=63 | N=58 | N=60 | N=60 | N=58 | N=61 | N=54 |
| β (95% CI) | 1.02 (-0.39, 2.44) | -1.73 (-4.23, 0.77) | -0.59 (-1.74, 0.57) | -0.47 (-3.03, 2.09) | -0.92 (-4.78, 2.93) | -2.02 (-4.66, 0.63) | -0.32 (-1.16, 0.52) | 34.68 (-1.27, 70.62) |
| p value | 0.15 | 0.17 | 0.31 | 0.72 | 0.63 | 0.13 | 0.45 | 0.058 |
|  |  |  |  |  |  |  |  |  |
| svPPA | N=82 | N=78 | N=68 | N=65 | N=73 | N=77 | N=72 | N=72 |
| β (95% CI) | 1.51 (0.38, 2.65) | -2.82 (-4.82, -0.83) | -1.65 (-2.56, -0.73) | -4.71 (-7.75, -1.66) | -4.90 (-7.80, -2.00) | -2.31 (-5.28, 0.66) | -1.32 (-2.22, -0.43) | 18.35 (-9.44, 46.15) |
| p value | 0.010 | **0.0060** | **6.05E-04** | **0.003** | **0.001** | 0.13 | **0.004** | 0.19 |
|  |  |  |  |  |  |  |  |  |
| CBS | N=92 | N=73 | N=59 | N=77 | N=80 | N=78 | N=78 | N=57 |
| β (95% CI) | 1.56 (0.63, 2.50) | -1.52 (-2.87, -0.16) | -0.60 (-1.25, 0.04) | -0.32 (-1.04, 0.40) | -0.82 (-3.06, 1.42) | -3.04 (-4.90, -1.17) | -0.61 (-1.07, -0.14) | 19.37 (-3.08, 41.82) |
| p value | **0.001** | 0.029 | 0.065 | 0.38 | 0.47 | **0.002** | 0.011 | 0.089 |
|  |  |  |  |  |  |  |  |  |
| PSP-RS | N=125 | N=121 | N=83 | N=118 | N=123 | N=120 | N=117 | N=85 |
| β (95% CI) | 0.92 (-0.15, 2.00) | -1.54 (-2.77, -0.30) | -0.20 (-0.92, 0.52) | -1.07 (-2.27, 0.12) | -2.79 (-4.64, -0.95) | -2.49 (-4.27, -0.72) | -0.64 (-1.09, -0.20) | 37.81 (13.28, 62.35) |
| p value | 0.093 | 0.015 | 0.58 | 0.077 | **0.003** | 0.006 | 0.005 | **0.003** |
|  |  |  |  |  |  |  |  |  |
| MBCI | N=67 | N=67 | N=57 | N=66 | N=66 | N=66 | N=67 | N=65 |
| β (95% CI) | 0.11 (-0.16, 0.38) | -0.96 (-1.65, -0.28) | -0.32 (-0.74, 0.09) | -0.58 (-1.48, 0.32) | -1.25 (-3.08, 0.57) | -1.24 (-2.73, 0.24) | -0.13 (-0.55, 0.30) | 14.95 (3.38, 26.51) |
| p value | 0.40 | 0.007 | 0.12 | 0.20 | 0.18 | 0.099 | 0.55 | 0.012 |
| β=regression coefficient; CI=confidence interval. β values, 95% CIs, and p values result from unadjusted linear regression models. β values are interpreted as the change in the mean value of the given disease indicator for each doubling of NfL concentration. p values < 0.00625 are considered as statistically significant after applying a Bonferroni correction for multiple testing and are shown in bold. The All FTD group includes patients with bvFTD, nfvPPA, svPPA, CBS or PSP-RS. | | | | | | | | |

**Table S24: Associations of baseline NfL with baseline disease indicators in FTD syndromes and MBCI – adjusted analysis**

|  | Association between NfL and: | | | | | | | |
| --- | --- | --- | --- | --- | --- | --- | --- | --- |
| Phenotype group | CDR®+NACC-FTLDsb | MoCA | NAT | MINT | Category fluency | Phonemic fluency | Digit span backward | Trails B |
| All FTD groups | N=680 | N=612 | N=493 | N=579 | N=598 | N=597 | N=594 | N=451 |
| β (95% CI) | 1.35 (0.97, 1.74) | -2.25 (-2.82, -1.68) | -0.87 (-1.14, -0.60) | -2.12 (-2.94, -1.31) | -4.10 (-5.01, -3.18) | -4.19 (-4.95, -3.43) | -0.81 (-1.04, -0.59) | 15.37 (6.06, 24.68) |
| p value | **1.30E-11** | **3.88E-14** | **4.97E-10** | **4.12E-07** | **1.98E-17** | **3.38E-25** | **2.81E-12** | **0.001** |
|  |  |  |  |  |  |  |  |  |
| bvFTD | N=305 | N=277 | N=225 | N=259 | N=262 | N=264 | N=266 | N=183 |
| β (95% CI) | 1.40 (0.90, 1.90) | -2.41 (-3.19, -1.64) | -1.01 (-1.37, -0.64) | -2.20 (-3.15, -1.25) | -5.48 (-6.73, -4.23) | -5.22 (-6.26, -4.18) | -0.98 (-1.31, -0.65) | 19.30 (6.59, 32.00) |
| p value | **7.60E-08** | **3.19E-09** | **1.72E-07** | **7.36E-06** | **6.23E-16** | **7.25E-20** | **1.48E-08** | **0.003** |
|  |  |  |  |  |  |  |  |  |
| nfvPPA | N=76 | N=63 | N=58 | N=60 | N=60 | N=58 | N=61 | N=54 |
| β (95% CI) | 1.35 (-0.14, 2.85) | -2.17 (-4.71, 0.38) | -0.75 (-1.99, 0.49) | -0.23 (-2.85, 2.40) | -1.84 (-5.98, 2.30) | -2.55 (-5.29, 0.20) | -0.57 (-1.42, 0.29) | 33.17 (-6.18, 72.51) |
| p value | 0.075 | 0.093 | 0.23 | 0.86 | 0.38 | 0.068 | 0.19 | 0.097 |
|  |  |  |  |  |  |  |  |  |
| svPPA | N=82 | N=78 | N=68 | N=65 | N=73 | N=77 | N=72 | N=72 |
| β (95% CI) | 1.61 (0.42, 2.80) | -2.80 (-4.84, -0.76) | -1.62 (-2.54, -0.70) | -4.88 (-7.91, -1.85) | -5.08 (-8.06, -2.11) | -2.53 (-5.50, 0.44) | -1.27 (-2.19, -0.34) | 16.08 (-12.23, 44.38) |
| p value | 0.009 | 0.008 | **8.07E-04** | **0.002** | **0.001** | 0.094 | **0.008** | 0.26 |
|  |  |  |  |  |  |  |  |  |
| CBS | N=92 | N=73 | N=59 | N=77 | N=80 | N=78 | N=78 | N=57 |
| β (95% CI) | 1.49 (0.45, 2.53) | -1.36 (-2.86, 0.13) | -0.44 (-1.12, 0.24) | -0.27 (-1.07, 0.53) | -0.36 (-2.80, 2.07) | -2.37 (-4.40, -0.33) | -0.69 (-1.22, -0.16) | 6.77 (-18.58, 32.12) |
| p value | **0.005** | 0.074 | 0.20 | 0.50 | 0.77 | 0.023 | 0.012 | 0.59 |
|  |  |  |  |  |  |  |  |  |
| PSP-RS | N=125 | N=121 | N=83 | N=118 | N=123 | N=120 | N=117 | N=85 |
| β (95% CI) | 1.08 (-0.04, 2.19) | -1.54 (-2.83, -0.24) | -0.16 (-0.89, 0.58) | -1.01 (-2.26, 0.23) | -2.88 (-4.74, -1.02) | -2.73 (-4.53, -0.93) | -0.66 (-1.12, -0.19) | 35.11 (11.74, 58.48) |
| p value | 0.059 | 0.020 | 0.67 | 0.11 | **0.003** | **0.003** | 0.0064 | **0.004** |
|  |  |  |  |  |  |  |  |  |
| MBCI | N=67 | N=67 | N=57 | N=66 | N=66 | N=66 | N=67 | N=65 |
| β (95% CI) | 0.14 (-0.15, 0.42) | -1.05 (-1.80, -0.30) | -0.24 (-0.71, 0.22) | -0.70 (-1.70, 0.31) | -1.35 (-3.48, 0.77) | -1.69 (-3.30, -0.09) | -0.22 (-0.70, 0.26) | 10.55 (-1.51, 22.61) |
| p value | 0.35 | 0.007 | 0.30 | 0.17 | 0.21 | 0.039 | 0.37 | 0.085 |
| β=regression coefficient; CI=confidence interval. β values, 95% CIs, and p values result from linear regression models that were adjusted for age, sex, symptom duration, and years of education. β values are interpreted as the change in the mean value of the given disease indicator for each doubling of NfL concentration. p values < 0.00625 are considered as statistically significant after applying a Bonferroni correction for multiple testing and are shown in bold. The All FTD group includes patients with bvFTD, nfvPPA, svPPA, CBS or PSP-RS. | | | | | | | | |

**Table S25: Associations of baseline biomarkers with rates of disease indicator change in all FTD syndromes combined**

|  | Association between baseline GFAP or NfL and rate of change over time of the given disease indicator | | | | |
| --- | --- | --- | --- | --- | --- |
|  |  | Unadjusted analysis | | Adjusting for age, sex, symptom duration, and years of education | |
| Disease indicator | N | β (95% CI) | p value | β (95% CI) | p value |
| Association with GFAP: |  |  |  |  |  |
|  |  |  |  |  |  |
| CDR®+NACC-FTLDsb | 55 | 1.16 (0.29, 2.02) | 0.010 | 1.16 (0.19, 2.13) | 0.020 |
| MoCa | 38 | -1.32 (-2.87, 0.24) | 0.094 | -1.57 (-3.55, 0.41) | 0.12 |
| MINT | 40 | -0.84 (-1.73, 0.05) | 0.063 | -1.07 (-2.24, 0.10) | 0.071 |
| Category fluency | 37 | -1.08 (-3.07, 0.92) | 0.28 | -0.82 (-3.31, 1.66) | 0.50 |
| Phonemic fluency | 39 | -0.81 (-2.07, 0.46) | 0.21 | -0.17 (-1.75, 1.42) | 0.83 |
| Digit span backward | 38 | 0.37 (-0.37, 1.11) | 0.32 | 0.35 (-0.61, 1.32) | 0.46 |
|  |  |  |  |  |  |
| Association with NfL: |  |  |  |  |  |
|  |  |  |  |  |  |
| CDR®+NACC-FTLDsb | 55 | 1.26 (0.60, 1.92) | 3.59E-04 | 1.25 (0.46, 2.04) | 0.002 |
| MoCa | 38 | -1.70 (-2.86, -0.54) | 0.005 | -1.95 (-3.28, -0.62) | 0.005 |
| MINT | 40 | -1.26 (-1.94, -0.57) | 6.54E-04 | -1.64 (-2.47, -0.81) | 3.16E-04 |
| Category fluency | 37 | -2.21 (-3.69, -0.72) | 0.005 | -2.13 (-4.01, -0.25) | 0.028 |
| Phonemic fluency | 39 | -1.12 (-2.17, -0.08) | 0.036 | -0.59 (-1.91, 0.73) | 0.37 |
| Digit span backward | 38 | -0.29 (-0.90, 0.32) | 0.34 | -0.47 (-1.22, 0.29) | 0.21 |
| β=regression coefficient; CI=confidence interval. CDR®+NACC-FTLDsb = CDR® Dementia Staging + National Alzheimer’s Disease Coordinating Center Frontotemporal Lobar Degeneration sum of boxes; MoCA = Montreal Cognitive Assessment; MINT = Multilingual Naming Test. β values, 95% CIs, and p values result from linear regression models. β values are interpreted as the change in the mean rate of change per year in the given outcome for each doubling of GFAP or NfL concentration. p values < 0.0083 are considered statistically significant after applying a Bonferroni correction for multiple testing. | | | | | |

**Table S26: Associations of baseline GFAP and NfL with survival after symptom onset**

|  |  |  | Unadjusted analysis | | Adjusting for age at symptom onset, sex, and symptom duration | | C-index | |
| --- | --- | --- | --- | --- | --- | --- | --- | --- |
| Marker/Disease group | N | No. (%) of patients who died | HR (95% CI) | P-value | HR (95% CI) | P-value | Multivariable model without marker | Multivariable model with marker |
| GFAP |  |  |  |  |  |  |  |  |
| bvFTD | 301 | 42 (14.0%) | 1.24 (0.91, 1.69) | 0.16 | 2.01 (1.32, 3.06) | 0.001 | 0.899 (0.795-1.002) | 0.928 (0.824-1.032) |
| nfvPPA | 76 | 16 (21.1%) | 0.46 (0.20, 1.08) | 0.075 | N/A | N/A | N/A | N/A |
| svPPA | 82 | 13 (15.9%) | 0.66 (0.31, 1.41) | 0.29 | N/A | N/A | N/A | N/A |
| CBS | 90 | 14 (15.6%) | 1.06 (0.48, 2.34) | 0.89 | N/A | N/A | N/A | N/A |
| PSP-RS | 129 | 20 (15.5%) | 1.57 (0.79, 3.13) | 0.20 | N/A | N/A | N/A | N/A |
| Combined group of bvFTD, nfvPPA, svPPA, CBS, and PSP-RS patients | 678 | 105 (15.5%) | 1.10 (0.88, 1.38) | 0.38 | 1.36 (1.09, 1.70) | 0.006 | 0.888 (0.819-0.958) | 0.896 (0.827-0.966) |
| NfL |  |  |  |  |  |  |  |  |
| bvFTD | 301 | 42 (14.0%) | 2.36 (1.81, 3.07) | 2.12E-10 | 2.97 (2.11, 4.18) | 4.67E-10 | 0.899 (0.795-1.002) | 0.955 (0.851-1.059) |
| nfvPPA | 76 | 16 (21.1%) | 0.64 (0.21, 1.94) | 0.43 | N/A | N/A | N/A | N/A |
| svPPA | 82 | 13 (15.9%) | 1.35 (0.69, 2.66) | 0.38 | N/A | N/A | N/A | N/A |
| CBS | 90 | 14 (15.6%) | 1.40 (0.90, 2.17) | 0.13 | N/A | N/A | N/A | N/A |
| PSP-RS | 129 | 20 (15.5%) | 1.79 (0.89, 3.60) | 0.10 | N/A | N/A | N/A | N/A |
| Combined group of bvFTD, nfvPPA, svPPA, CBS, and PSP-RS patients | 678 | 105 (15.5%) | 1.78 (1.49, 2.13) | 3.02E-10 | 2.39 (1.89, 3.02) | 4.16E-13 | 0.888 (0.819-0.958) | 0.917 (0.847-0.986) |
| HR=hazard ratio; CI=confidence interval. HRs, 95% CIs, and p-values result from Cox proportional hazards regression models. HRs correspond to each doubling in GFAP or NfL concentration. P-values < 0.05 are considered as statistically significant. Multivariable analysis adjusting for age at symptom onset, sex, and symptom duration was not performed for the nfvPPA, svPPA, CBS, and PSP-RS groups owing to the small number of deaths in these groups. | | | | | | | | |

**Table S27: Comparisons of baseline biomarkers or their ratio by mutation status in all FTD syndromes combined**

|  |  |  | Unadjusted analysis | | Adjusting for age, sex and symptom duration | |
| --- | --- | --- | --- | --- | --- | --- |
| Marker/Disease group/Mutation Status | N | Median (minimum, maximum) concentration | β (95% CI) | p value | β (95% CI) | p value |
| GFAP |  |  |  |  |  |  |
| No mutation | 549 | 227.7 (39.5, 2544.5) | 0.00 (reference) | N/A | 0.00 (reference) | N/A |
| *C9orf72* mutation | 60 | 216.6 (68.8, 1114.6) | -0.19 (-0.41, 0.03) | 0.089 | -0.03 (-0.24, 0.19) | 0.82 |
| *GRN* mutation | 34 | 260.4 (116.6, 1180.3) | 0.26 (-0.03, 0.55) | 0.080 | 0.33 (0.06, 0.60) | 0.017 |
| *MAPT* mutation | 35 | 181.3 (48.2, 1840.0) | -0.26 (-0.55, 0.02) | 0.070 | 0.00 (-0.28, 0.29) | 0.98 |
| NfL |  |  |  |  |  |  |
| No mutation | 549 | 26.5 (4.9, 177.6) | 0.00 (reference) | N/A | 0.00 (reference) | N/A |
| *C9orf72* mutation | 60 | 30.6 (5.0, 125.4) | -0.07 (-0.31, 0.16) | 0.54 | 0.02 (-0.21, 0.25) | 0.87 |
| *GRN* mutation | 34 | 63.6 (4.7, 246.4) | 1.09 (0.78, 1.40) | 8.21E-12 | 1.10 (0.80, 1.39) | 1.62E-12 |
| *MAPT* mutation | 35 | 18.7 (4.7, 164.0) | -0.47 (-0.78, -0.17) | 0.002 | -0.31 (-0.62, 0.00) | 0.054 |
| GFAP/NfL ratio |  |  |  |  |  |  |
| No mutation | 549 | 8.8 (0.6, 212.9) | 0.00 (reference) | N/A | 0.00 (reference) | N/A |
| *C9orf72* mutation | 60 | 8.6 (1.9, 23.9) | -0.12 (-0.37, 0.13) | 0.35 | -0.05 (-0.30, 0.21) | 0.73 |
| *GRN* mutation | 34 | 4.8 (2.1, 31.4) | -0.83 (-1.16, -0.51) | 6.81E-07 | -0.76 (-1.09, -0.44) | 4.76E-06 |
| *MAPT* mutation | 35 | 10.3 (1.7, 51.4) | 0.21 (-0.11, 0.53) | 0.20 | 0.31 (-0.03, 0.65) | 0.073 |
| β=regression coefficient; CI=confidence interval. β values, 95% CIs, and p values result from linear regression models. β values are interpreted as the difference in the mean GFAP, NfL, or GFAP/NfL concentration (on the base-2 logarithm scale) for the given mutation group in comparison to participants without a mutation. p values <0.0083 are considered as statistically significant after applying a Bonferroni correction for multiple testing for comparisons in the present table and in Table S28. | | | | | | |

**Table S28: Comparisons of baseline biomarkers or their ratio among mutation groups in all FTD syndromes combined**

|  |  |  | Adjusting for age, sex, and symptom duration | |
| --- | --- | --- | --- | --- |
| Marker/Disease group/Mutation Status | N | Median (minimum, maximum) concentration | p value vs. *GRN* mutation | p value vs. *MAPT* mutation |
| GFAP |  |  |  |  |
| *C9orf72* mutation | 60 | 216.6 (68.8, 1114.6) | 0.024 | 0.70 |
| *GRN* mutation | 34 | 260.4 (116.6, 1180.3) | --- | 0.18 |
| *MAPT* mutation | 35 | 181.3 (48.2, 1840.0) | --- | --- |
| NfL |  |  |  |  |
| *C9orf72* mutation | 60 | 30.6 (5.0, 125.4) | 2.66E-06 | 0.081 |
| *GRN* mutation | 34 | 63.6 (4.7, 246.4) | --- | 9.45E-08 |
| *MAPT* mutation | 35 | 18.7 (4.7, 164.0) | --- | --- |
| GFAP/NfL ratio |  |  |  |  |
| *C9orf72* mutation | 60 | 8.6 (1.9, 23.9) | 1.38E-05 | 0.019 |
| *GRN* mutation | 34 | 4.8 (2.1, 31.4) | --- | 3.73E-05 |
| *MAPT* mutation | 35 | 10.3 (1.7, 51.4) | --- | --- |
| p values result from linear regression models that were adjusted for age, sex, and symptom duration. p values < 0.0083 are considered as statistically significant after applying a Bonferroni correction for multiple testing for comparisons in the present table and in Table S27. | | | | |

**Table S29:** **Differences in baseline biomarkers or their ratio between FTLD-tau or FTLD-TDP pathology in FTD syndromes**

|  |  |  | Unadjusted analysis | | | Adjusting for age at GFAP or NfL measurement, sex, symptom duration, and age at death | | |
| --- | --- | --- | --- | --- | --- | --- | --- | --- |
| Marker/Disease group | N | Median (minimum, maximum) concentration | β (95% CI) | p value | AUC (95% CI) | β (95% CI) | p value | AUC (95% CI) |
| GFAP |  |  |  |  |  |  |  |  |
| Tau-only pathology | 71 | 234.0 (65.0, 850.7) | 0.00 (reference) | N/A | N/A | 0.00 (reference) | N/A | N/A |
| TDP-43-only pathology | 36 | 252.4 (66.4, 1180.3) | 0.08 (-0.24, 0.41) | 0.60 | 0.53 (0.41, 0.66) | 0.12 (-0.21, 0.44) | 0.47 | 0.55 (0.43, 0.67) |
| NfL |  |  |  |  |  |  |  |  |
| Tau-only pathology | 71 | 28.9 (11.2, 75.9) | 0.00 (reference) | N/A | N/A | 0.00 (reference) | N/A | N/A |
| TDP-43-only pathology | 36 | 52.2 (20.0, 246.4) | 0.84 (0.56, 1.13) | 4.53E-08 | 0.78 (0.69, 0.88) | 0.78 (0.50, 1.06) | 1.76E-07 | 0.78 (0.69, 0.87) |
| GFAP/NfL ratio |  |  |  |  |  |  |  |  |
| Tau-only pathology | 71 | 7.5 (3.3, 42.6) | 0.00 (reference) | N/A | N/A | 0.00 (reference) | N/A | N/A |
| TDP-43-only pathology | 36 | 4.8 (0.6, 20.9) | -0.76 (-1.08, -0.44) | 8.70E-06 | 0.75 (0.65, 0.85) | -0.66 (-0.98, -0.34) | 7.64E-05 | 0.73 (0.63, 0.83) |
| β=regression coefficient; CI=confidence interval; AUC=area under the receiver operating curve. β values, 95% CIs, and p values result from linear regression models. β values are interpreted as the difference in the mean GFAP, NfL or GFAP/NfL concentration (on the base-2 logarithm scale) for TDP-43-only pathology compared to tau-only pathology. p values <0.025 are considered as statistically significant after applying a Bonferroni correction for multiple testing. | | | | | | | | |
